# Outcome measures from international older adult care home intervention research: a scoping review

**DOI:** 10.1093/ageing/afad069

**Published:** 2023-05-16

**Authors:** Sarah Kelly, Andy Cowan, Gizdem Akdur, Lisa Irvine, Guy Peryer, Silje Welsh, Stacey Rand, Iain A Lang, Ann-Marie Towers, Karen Spilsbury, Anne Killett, Adam Lee Gordon, Barbara Hanratty, Liz Jones, Julienne Meyer, Claire Goodman, Jennifer Kirsty Burton

**Affiliations:** Cambridge Public Health, University of Cambridge, East Forvie Site, Robinson Way, Cambridge CB2 0SR, UK; THIS Institute, University of Cambridge, Clifford Allbutt Building, Cambridge Biomedical Campus, Cambridge CB2 0AH, UK; Cambridge Public Health, University of Cambridge, East Forvie Site, Robinson Way, Cambridge CB2 0SR, UK; Centre for Research in Public Health and Community Care, University of Hertfordshire, College Lane, Hatfield AL10 9AB, UK; Centre for Research in Public Health and Community Care, University of Hertfordshire, College Lane, Hatfield AL10 9AB, UK; School of Health Sciences, University of East Anglia, Norwich Research Park, Norwich NR4 7TJ, UK; NIHR Applied Research Collaboration, East of England, CB2 8AH, UK; School of Cardiovascular and Metabolic Health, University of Glasgow, New Lister Building, Glasgow Royal Infirmary, Alexandra Parade, Glasgow G31 2ER, UK; Personal Social Services Research Unit, University of Kent, Cornwallis Central, Canterbury CT2 7NF, UK; Department of Health and Community Sciences, University of Exeter, St Luke’s Campus, Exeter EX1 2LU, UK; NIHR Applied Research Collaboration, South West Peninsula, EX1 2LU, UK; Centre for Health Services Studies, Cornwallis Central, University of Kent, Canterbury CT2 7NF, UK; NIHR Applied Research Collaboration, Kent Surrey and Sussex, BN3 7HZ, UK; School of Healthcare, University of Leeds, Baines Wing, Leeds LS2 9JT, UK; NIHR Applied Research Collaboration, Yorkshire and Humber, BD9 6RJ, UK; School of Health Sciences, University of East Anglia, Norwich Research Park, Norwich NR4 7TJ, UK; NIHR Applied Research Collaboration, East of England, CB2 8AH, UK; Unit of Injury, Recovery and Inflammation Sciences (IRIS), School of Medicine, University of Nottingham, Royal Derby Hospital, Uttoxeter Road, Derby DE22 3DT, UK; NIHR Applied Research Collaboration, East Midlands, LE5 4PW, UK; Population Health Sciences Institute, Newcastle University, Campus for Ageing and Vitality, Newcastle-upon-Tyne NE4 5PL, UK; NIHR Applied Research Collaboration, North East and North Cumbria, NE3 3XT, UK; National Care Forum, Friars House, Manor House Drive, Coventry CV1 2TE, UK; National Care Forum, Friars House, Manor House Drive, Coventry CV1 2TE, UK; City, University of London, Northampton Square, London EC1V 0HB, UK; Centre for Research in Public Health and Community Care, University of Hertfordshire, College Lane, Hatfield AL10 9AB, UK; NIHR Applied Research Collaboration, East of England, CB2 8AH, UK; School of Cardiovascular and Metabolic Health, University of Glasgow, New Lister Building, Glasgow Royal Infirmary, Alexandra Parade, Glasgow G31 2ER, UK

**Keywords:** care home, homes for the aged, long-term care, outcome measures, core outcome set, older people

## Abstract

**Background:**

Care homes are increasingly important settings for intervention research to enhance evidence-informed care. For such research to demonstrate effectiveness, it is essential that measures are appropriate for the population, setting and practice contexts.

**Objective:**

To identify care home intervention studies and describe the resident outcome measures used.

**Design:**

Scoping review.

**Methods:**

We reviewed international care home research published from 2015 to August 2022. We searched MEDLINE, EMBASE, CINAHL and ASSIA. We included any intervention study conducted in a care home, reporting resident outcomes. We extracted resident outcome measures, organised these using the domains of an adapted framework and described their use.

**Results:**

From 7,330 records screened, we included 396 datasets reported in 436 publications. These included 12,167 care homes and 836,842 residents, with an average of 80 residents per study. The studies evaluated 859 unique resident outcomes 2,030 times using 732 outcome measures. Outcomes were evaluated between 1 and 112 times, with 75.1% of outcomes evaluated only once. Outcome measures were used 1–120 times, with 68.4% of measures used only once. Only 14 measures were used ≥20 times. Functional status, mood & behaviour and medications were the commonest outcome domains assessed. More than half of outcomes were assessed using scales, with a fifth using existing records or administrative data.

**Conclusions:**

There is significant heterogeneity in the choice and assessment of outcomes for intervention research in care homes. There is an urgent need to develop a consensus on useful and sensitive tools for care homes, working with residents, families and friends and staff.

## Key Points

- Intervention studies in care homes have the potential to shape evidence-informed care.
- Resident outcome measures used in such research must be contextually appropriate.
- We found significant heterogeneity in outcome assessment in international care home research, disproportionate to the diversity of interventions tested.
- This contributes to research waste as evidence is more difficult to synthesise; thus, we welcome the development of core outcome sets for care homes.
- However, there is an urgent need to develop a consensus on useful and sensitive tools for care homes, working with residents, families and friends and staff.

## Background

UK care homes provide a home and range of services for adults with complex care needs. Older adults account for most of the UK care home population and are the focus of this review. Residents typically live with frailty, disability, multimorbidity and/or dementia [1, 2]. The need for care home places is anticipated to grow [3], due to the increased prevalence and complexity of care needs associated with population ageing [4]. The impact of the COVID-19 pandemic on residents [5, 6] has emphasised the need for better data to understand the characteristics and needs of this population [7].

To ensure people can live well in care homes, it is important that approaches to care are evidence-informed. However, producing this evidence requires an understanding of how to describe and measure relevant resident outcomes of care delivery, including health, function and quality of life. Intervention research conducted in care homes has been increasing rapidly [8, 9]. However, care home research is challenging [10] and many findings from well-designed trials conducted in care homes have been equivocal [9].

Using research measures and indices, designed for clinical settings, in care homes is methodologically challenging. The consideration of appropriate outcome measures should reflect the objectives of long-term care to meet residents’ needs and sustain their quality of life, rather than yield measurable improvements in health or functioning. The use of patient-reported outcome measures (PROMS) is challenging due to high levels of cognitive impairment [11], and staff often act as proxies. However, unlike health care settings, the majority of the care home workforce do not have clinical training and the way they interpret and understand such scales is not well understood [11]. Care home research must respect the care home context and minimise burden and intrusion [12]. Specific evidence on how best to collect data and measure outcomes in these settings is needed to inform future study design and maximise the usefulness of care home research for practice and policy.

A previous mapping review identified the interventions and outcomes studied in care home trials [8]. However, to capture both recent increased research activity and broader methodological approaches used in testing interventions provides an opportunity to explore how outcomes are measured and identify measurement tools used in care home research. Our aim in this scoping review was to identify intervention studies in care homes and map the key outcomes and outcome measures used and their frequency of use. It was undertaken to inform the development of a minimum data set for use by UK care homes [13] for which purpose there was involvement from care home staff and family members.

## Methods

### Review question and objectives

We undertook a scoping review of the international literature to

- identify and characterise intervention studies conducted in care homes
- identify and categorise resident outcomes
- describe the frequency of use of measurement tools (‘outcome measures’)
- describe the data sources used to generate outcome measures

We used a scoping review approach because of the value of this method to explore available literature and map the nature and type of available evidence [14, 15].

### Care home setting

We recognise that there is heterogeneity in the terminology used to describe long-term care settings internationally [16], with the term ‘nursing home’ previously recommended [17]. However, there are important structural differences in residential long-term care provision internationally [18], and it is important terminology that reflects the organisation and delivery of care. Therefore, for consistency, we have used the term ‘care home’ inclusively to mean long-term residential care settings where older adults receive 24-hour care and support, with or without on-site registered nursing staff.

### Conduct and reporting

Methods for the conduct and reporting of the scoping review followed the guidelines of the PRISMA-ScR statement [15]. The protocol was registered on PROSPERO (CRD42020155923) nested with a related systematic review [19]. For this scoping review, we focused on studies published after 2015 and undertaken in OECD (Organisation for Economic Cooperation and Development) countries [20]. This reflected our interest in mapping the most recent research practice from countries of similar economic status and health and social care resources to the UK, to inform the development of a minimum data set.

### Information sources and search strategy

We searched the following databases: MEDLINE, EMBASE, CINAHL and ASSIA on December 2019, updated to August 2022. Our search strategy was developed with advice from an experienced information specialist, reported in full in Supplementary Materials Appendix 1. The database search strategy combined a range of keywords and medical subject heading terms related to (1) intervention studies and (2) care home settings, including other terms to identify long-term care facilities internationally [16]. We searched for grey literature, including the Open Grey database and websites of national and professional organisations relevant to care homes and older people (Appendix 1). Where abstracts or theses were identified, we searched for subsequent peer-reviewed publications.

### Eligibility criteria

Any type of intervention study conducted in care homes between 2015 and 2022, including randomised controlled trials (RCTs), non-randomised controlled trials, before and after studies, quality improvement studies and other intervention designs was eligible for inclusion. We excluded reviews and conference abstracts. We only included papers published in English. Studies in which the intervention was delivered to a mixed population including care home residents and older adults living in other community settings were excluded.

Outcomes (and associated outcome measures) eligible for inclusion related to resident health and care outcomes including, but not limited to, measures of function, quality of life, quality of care, resident- and family-reported outcomes and health resource use. Studies that reported staff outcomes that did not also report outcomes for residents were excluded. Studies that were solely qualitative (e.g. collected data using interviews and focus groups) were excluded.

### Screening and data extraction

We managed the database search using Covidence software [21]. Titles and abstracts were screened by two independent reviewers (from SK, AC, GA, LI, GP and JKB) with disagreements resolved by a third experienced reviewer (from SK, LI, GP and JKB). Full texts were screened by single experienced reviewers (from SK, AC and JKB) with uncertainties resolved within the team by discussion.

We undertook data extraction using a structured Excel template with data recorded at the study level and at the outcome level. Data from each study were extracted by a single reviewer (from SK, AC, GA, LI, SW, SR, IL, AMT, KS, AK and JKB), with guidance provided to improve consistency. Following data extraction, the database was subject to review and cleaning to align reporting across all studies. For each study included, data were extracted relating to author, year of publication(s)(based on online publication), country(ies), study design, number of homes, number of resident participants, type of care home (residential, nursing or both), study aim, eligibility for participation (all residents or subset), description of intervention, description of any control, timing of outcome assessment (converted months), outcomes and outcome measures reported including how (electronic/paper/both) and by whom these were reported. We did not a conduct formal quality assessment of the included studies.

### Synthesis

Using the aim and intervention description, we further classified interventions to describe the target of the intervention and type of intervention used. The approach taken was adapted from that used by Gordon *et al.* in their mapping review [8].

To organise the large number of outcomes measures identified, we used the main domains of the International Resident Assessment Instrument for Long-Term Care Facilities (interRAI-LTCF) [22] as the basis of an outline framework, similar to a best-fit framework synthesis approach [23, 24]. This was chosen as it was specifically developed for care homes and has clearly defined domains for a range of outcomes relevant to residents. The interRAI-LTCF domains [22] used to categorise outcomes were

- activities & interests (activity preferences and involvement and daytime sleep);
- cognition (including delirium and dementia);
- communication & vision;
- continence;
- disease & diagnoses;
- functional status (including activities of daily living, locomotion and physical function);
- health conditions (including falls, sleep, fatigue, pain);
- medications (including nutritional supplements);
- mood & behaviour;
- oral & nutritional status (including anthropometry, dehydration and dental/oral cavity issues);
- psychosocial wellbeing (social and unsettled relationships, sense of involvement, loneliness and major life stressors);
- skin condition (including pressure ulcers);
- treatments & procedures (including oral health outcomes, hospital/emergency department admissions/other transfers & restraint use).

**Figure 1:**
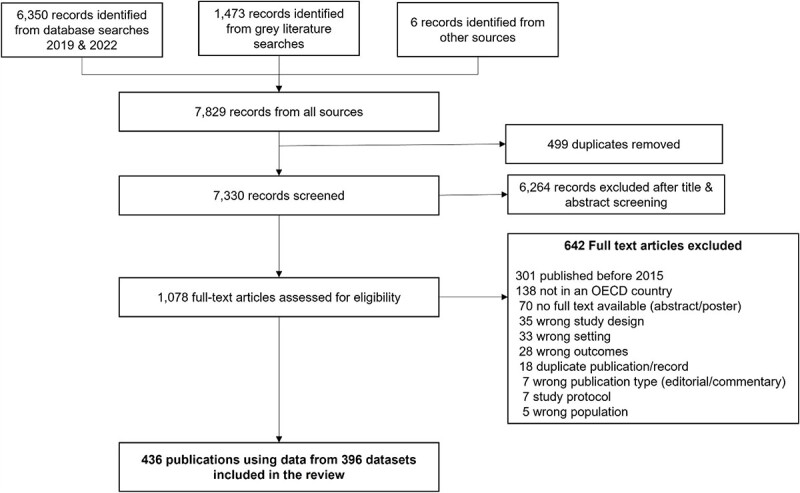
PRISMA Flow diagram summarising study selection.

We note that there are other interRAI instruments for outcomes important to care homes, including palliative care [25]; however, we used the LTCF domains for this review. Some outcomes could potentially fit into more than one domain; for example, oral health–related outcomes may be classified under ‘oral & nutritional status’ or ‘treatments & procedures’, as both have some dental/oral health sub-categories. In such cases, we chose one domain, assigning them to ‘oral & nutritional status’. Some additional important outcomes did not clearly fit into any interRAI-LTCF domain, including blood tests, microbiological & virological specimens, anticipatory care/palliative/end-of-life care, mortality and quality of life. We added separate domains to our classification framework to include these. We also included an ‘other’ domain for outcomes that could not be classified. The frequency of use was calculated by counting the number of times each measure was used. Where similar versions of the same measurement tool were used (e.g. translated versions; adapted versions for use in care homes), these were combined. Finally, we classified how an outcome was measured noting whether it was based on a biological test/measure, new data collection for study reporting, the review of existing records/administrative data, using a scale, or captured by technology.

### Participant and public involvement and engagement

Emerging results of the review were shared with people working in care homes and with family members of care home residents, to gauge feedback about the utility and feasibility of use of these types of measures in care homes and to compare research findings to experiences in practice.

## Results

### Search results

From 7,330 records screened, 1,078 full texts were reviewed and 436 papers reporting data from 396 datasets were included. References for all included studies are listed in Supplementary materials Appendix 2. The PRISMA flow chart is shown in Figure 1. Our findings include published journal articles (*n* = 430) and student theses (*n* = 6).

### Characteristics of included studies

#### Year of publication

A growing numbers of studies were published year-on-year, with 43 in 2015, rising to 66 in 2021.

#### Country

Studies were conducted in 27 out of the 38 OECD countries, and four studies were undertaken in multiple countries. Of studies in a single country, the top five were USA (*n* = 66, 16.7%), Australia (*n* = 50, 12.6%), UK (*n* = 43, 10.9%), Germany (*n* = 30, 7.6%) and Canada (*n* = 26, 6.6%).

#### Study design

Just under half of all the studies used randomised controlled trial designs (*n* = 193, 48.7%). Other designs used were pilot/feasibility studies (*n* = 87, 22.0%), before-and-after studies (*n* = 66, 16.7%), non-randomised studies (*n* = 22, 5.6%), and studies (*n* = 28, 7.1%) of another design.

#### Setting

Most studies (*n* = 318, 80.3%) were undertaken in homes with nursing staff, with *n* = 43 (10.9%) in residential homes and *n* = 35 (8.8%) including both settings.

#### Number of homes per study

A total of 12,167 homes were included in the studies. The number of homes included in each study varied from 1 to 1,238, with a median of six homes per study [inter-quartile range (IQR) 15]. Six studies did not report the number of homes involved in their research.

#### Number of residents per study

A total of 836,842 residents were included in the studies. The number of residents included in each study varied from 2 to 127,497, with a median of 80 residents per study [IQR 196]. Twenty-five studies did not report the number of residents involved; their unit of analysis was usually the care home and not the residents.

Full details of the year of publication, country, study design and distribution of sample sizes are summarised in Appendix 3, Supplementary Tables 1–4.

#### Eligibility

Eligibility to participate in the study was restricted in most of the studies (*n* = 302, 76.3%), with *n* = 94 (23.7%) open to all residents in the home at the time of the study.

#### Timing of outcome assessment

Outcome assessment timing was varied, ranging from 0.2 to 48 months (mean of 6.6 months), taking account of the longest point of outcome assessment reported in the datasets. Eight studies undertook continuous outcome assessment, and three assessed their intervention immediately at the end of sessions. Ten studies did not report when outcome assessment was undertaken.

### Classification of interventions

Interventions targeted a range of areas affecting residents within included homes: medicine management & prescribing (*n* = 56, 14.1%), physical function/performance/activity (*n* = 21, 5.3%), cognition (*n* = 20, 5.1%), hospital transfer/length of stay (*n* = 19, 4.8%) and oral health (*n* = 19, 4.8%) were the five commonest. The type of interventions was also varied with multi-component (*n* = 90, 22.7%), exercise (*n* = 41, 10.4%), education/training (*n* = 38, 9.6%), technology (*n* = 28, 7.1%) and pharmacological (*n* = 24, 6.1) as the five commonest. The characteristics of a study table summarising study design, the number of homes and residents, intervention target and type are included in Appendix 4, Supplementary Table 5. Full reporting of the aim, intervention, and control (where applicable) for the included studies is in Appendix 5, Supplementary Table 6.

### Resident outcomes and categorisation

The 396 included datasets reported a total of 2,030 resident outcomes (range 1–37 outcomes, and median four outcomes [IQR four] reported per dataset). Of the 2,030 resident outcomes assessed, 859 were unique resident outcomes. These outcomes were assessed between 1 and 112 times; 645 (75.1%) were only assessed once.

**Figure 2:**
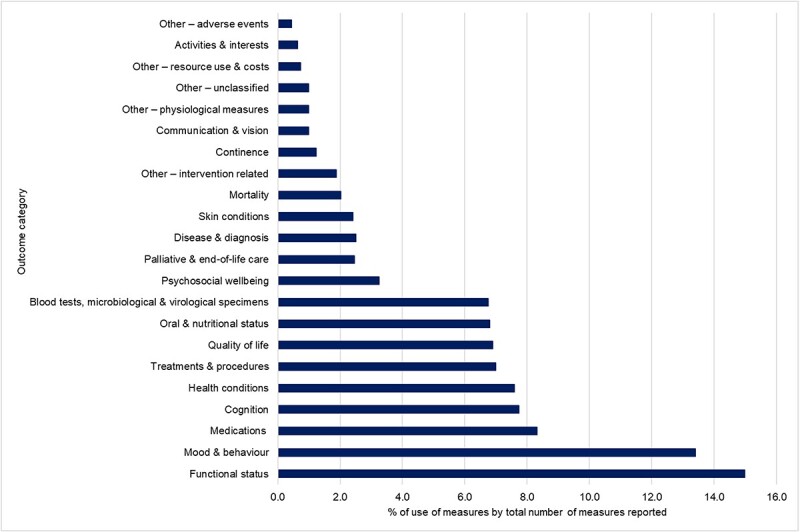
Summary of percentage of measures per adapted inter-RAI LTCF domain reported, of all outcome measures reported.

**Figure 3:**
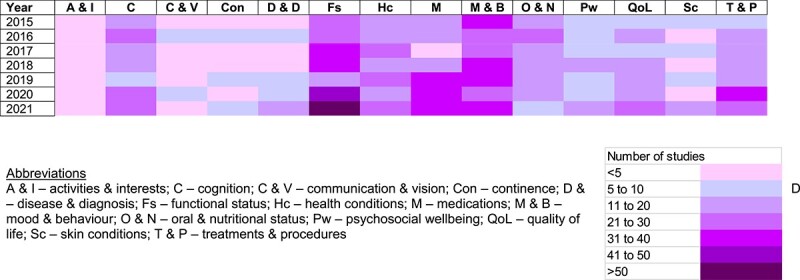
Frequency of reporting of measures by Inter-RAI LTCF Framework domain between 2015 and 2021.

Using the adapted interRAI LTCF-domains, all outcomes were classified. Of the 2,030 outcomes, the most commonly evaluated domain was functional status (*n* = 304, 15.0%) followed by mood & behaviour (*n* = 272, 13.4%), medications (*n* = 169, 8.3%), cognition (*n* = 157, 7.7%), health conditions (*n* = 154, 7.6%), treatments & procedures (*n* = 142, 7.0%), quality of life (*n* = 140, 6.9%) and oral & nutritional status (*n* = 138, 6.8%) (Figure 2). The frequency of outcome use for the top 14 domains between 2015 and 2021 (reporting complete years only) is shown in Figure 3.

### Outcome measurement

A total of 732 outcome measures were used between 1 and 120 times; 501 measures (68.4%) were used only once. Fourteen measures were used ≥20 times, of which only four were used more >50 times (Table 1). A total of 419 scales were used on 1,036 occasions. These scales were used between 1 and 53 times each; 297 scales (70.9%) were used only once. Only 35 scales were used ≥5 times, of which nine scales were used >20 times. These nine scales (and adapted interRAI LTCF-domains) were the Neuropsychiatric Inventory (mood & behaviour), EQ-5D (quality of life), Mini Mental State Examination (cognition), Cohen Mansfield Agitation Inventory (mood & behaviour), Geriatric Depression Scale (mood & behaviour), Cornell Scale for Depression in Dementia (mood & behaviour), Barthel Index (functional status), the Timed Up and Go Test (functional status), and Quality of Life in Late Stage Dementia (quality of life) (Table 2). The full reporting of the frequency of use of scale-based measures is shown in Appendix 6, Supplementary Table 7.

**Table 1:** Frequency of use of all outcome measures used ≥20 times

Outcome measure	Number of uses
Care home/resident records	120
Blood tests	98
Medical records/charts	56
Neuropsychiatric Inventory (NPI)	53
EuroQoL (EQ-5D)	43
Mini Mental State Examination (MMSE)	41
Cohen Mansfield Agitation Inventory (CMAI)	37
Outcome measure method of assessment not reported	*35*
Geriatric Depression Scale (GDS)	30
Cornell Scale for Depression in Dementia (CSDD)	29
Barthel Index (BI)	27
Timed-up-and-go-Test (TUGT)	26
Quality of Life in Late-Stage Dementia (QUALID)	21
Dynamometer	20

**Table 2:** Frequency of use of the scale-based outcome measures used ≥5 times

Outcome measure	Number of uses
Neuropsychiatric Inventory (NPI) [Neuropsychiatric Inventory Nursing Home Version]	53 [20]
EuroQoL (EQ-5D)	43
Mini Mental State Examination	41
Cohen Mansfield Agitation Inventory	37
Geriatric Depression Scale	30
Cornell Scale for Depression in Dementia	29
Barthel Index	27
Timed Up and Go Test	26
QUALID: Quality of Life in Late-Stage Dementia	21
QOL-AD: Quality of Life in Alzheimer’s Disease	19
Short Physical Performance Battery	18
QUALIDEM: Quality of Life for People with Dementia	12
Berg Balance Scale	12
DEMQoL: Dementia Quality of Life	11
Short Form Health Survey score	11
Pittsburgh Sleep Quality Index	10
Palliative care Outcome Scale	9
Tinetti Performance Oriented Mobility Test	9
Dementia Care Mapping	8
Montreal Cognitive Assessment	8
Trail making test	8
Hospital Anxiety and Depression Scale	7
Mini Nutritional Assessment	7
Activities of Daily Living (ADL) score	6
Falls Efficacy Scale	6
Apathy Evaluation Scale	6
Observed Emotion Rating Scale	6
WHO International Classification of Functioning, Disability and Health	6
Brief Pain Inventory	5
Confusion Assessment Method	5
Functional Independence Measure	5
Geriatric Anxiety Inventory	5
Medication Appropriateness Index	5
Mobilisation-Observation-Behaviour-Intensity-Dementia Pain Scale	5
Pain Assessment in Advanced Dementia	5

### Data sources to generate outcome measures

Sources of outcome measures were scales (*n* = 1,036, 51.0%), review of existing records or administrative data (*n* = 430, 21.2%), biological tests or measurements (*n* = 293, 14.4%), new data collection for the study (*n* = 190, 9.4%) and data derived from technology (*n* = 58, 2.9%). For 23 measures (1.1%), the data source used for generating the measure was not reported.

Whether outcomes were collected using electronic or paper methods was poorly reported. For 74.9%, *n* = 1,521 of outcomes, this was not reported or unclear. Where there was a clear statement of recording, 15.9%, *n* = 323 used electronic outcome measurement, 8.8%, *n* = 178 used paper-based methods and 0.4%, *n* = 8 used both.

Outcome data were reported by care home staff (*n* = 491, 24.2%), residents (*n* = 351, 17.3%), researchers (*n* = 289, 14.2%), healthcare professionals (*n* = 232, 11.4%) and others including relatives (*n* = 114, 5.6%). In over a quarter of cases, who reported an outcome was unclear or not reported (*n* = 553, 27.2%).

## Discussion

### Overview

Our scoping review characterises the care home intervention literature from 2015 to 2022, with the evidence of a growing research area. Significant heterogeneity was seen in outcome measurement with a wide range of measures used, but the majority of these were used only once. We found a bias towards the collection of outcomes using scales. Functional status was the most common focus of outcome measurement. There was a poor quality of reporting on whether outcomes were captured using electronic or paper measures and who reported outcome measures. Sample size data were skewed with most studies involving <100 residents. Largest sample sizes were associated with the use of routinely collected administrative data, evaluating changes in policy at a regional or national level.

While care home research is undertaken to address a broad range of problems, challenges and conditions for the individuals living there, the diversity of measures used to measure similar attributes creates challenges learning from research findings and making recommendations for practice and policy.

### Findings in context

There is a continued growth of care home intervention research, first described by Gordon *et al.* in their review of trials published on 2009 [8]. Furthermore, interventions targeting quality of life, valued by care home residents and their families [26], have been more widely studied in recent years. However, outcomes around continence and communication & vision accounted for 1.2% and 1.0%, respectively, despite their prevalence and significance to everyday care [27, 28].

Many of the outcome measures use measurement instruments developed in hospitals or other clinical settings. Most care home residents are living with greater levels of frailty and in worse health than older people in other settings [9, 29], and challenges in implementing tools developed elsewhere have been reported [30]. Of the nine scales used in ≥20 studies, two are for depression, two are for quality of life, one is for neuropsychiatric symptoms, one is for agitation, one is for cognition, one is for activities of daily living and one is a test of functional mobility. Of these, only the Neuropsychiatric Inventory has a specific version for use in nursing home settings and this version was used in 20/53 instances (37.7%). There is some evidence of limited clinical utility in care homes and for residents with dementia of the depression measures [31, 32]. Even outside of the care home setting, limitations in the Barthel Index for measuring changes in treatment effects in terms of responsiveness have been reported, with marked ceiling effects [33, 34].

It was interesting to note the paucity of measures tailored to social care and concepts such as social-care-related quality of life, which have been shown to be acceptable and feasible to capture [35]. The dominance of clinical and health-orientated measures is apparent throughout the international literature summarised in this review. This issue is recognised across aged-care service users, where more recent tools evaluating quality of life are noted to focus on more than health status alone [36].

Fully reviewing the measurement properties of outcome measures for care homes is outside the scope of this review. There is a need for more formal evaluation of the psychometric and measurement properties of tools used for research in care homes. An earlier review of quality-of-life measures for use in care homes found 13 measures used, noting that no data were available about responsiveness for any of these measures [37]. A proposed repository of trials in care homes [38] may provide a mechanism for more in-depth research.

Quality of life is well recognised to be of particular importance to the older adult population living in care homes [26]. However, measurement can be challenging [11], as can sensitivity of measures to the impact of interventions [35, 39, 40]. It is recognised that not all older people living in care homes are able to provide self-reported outcomes, particularly for complex measures such as quality of life. Therefore, it is essential that measures are designed inclusively to enable participation and allowing both self-report and proxy report is ideal [11]. However, proxy-reported measures also need to have validity when another person is reporting quality of life on residents’ behalf, an active topic of ongoing research internationally [41–43].

The heterogeneity of outcome assessment approaches is not unique to the care home research field [44, 45] but is important in this field given the growth of research and interest in learning how to use findings to inform practice. It is essential to avoid research waste, and the use of such a diverse range of measures, often used just once, contributes to this problem. Some work is underway, through the COMET initiative [46], to develop core outcome sets (COSs) for future trials in care homes [47, 48]. A COS is an agreed ‘standardised set of outcomes that should be measured and reported, as a minimum, in all clinical trials in specific areas’ [46].

### Strengths and limitations

Strengths of this review include a structured search of four databases, the inclusion of international studies and the scope: we have collated outcome measurement instruments across a wide range of different interventions and study designs to reflect the breadth of recent research in care homes. We have mapped outcomes to an adapted version of an international framework for long-term care, enabling both the tool/measure analysis and domain analysis of the body of literature.

Limitations include our focus on higher-income countries, omitting research undertaken in China, Hong Kong, Indonesia, India and other non-OECD countries, accounting for 138 studies excluded at full-text review. We cannot comment on the quality of the literature identified as we did not undertake formal quality assessment. We have not described the totality of outcome assessment in the included studies as we focused on resident outcome measures. Those related to staff and relative experiences are not captured, although their views of residents are included. Of note, these findings focus on outcome measures reported in research papers, and so they reflect the types of outcomes which researchers felt it was important to measure in the context of a given intervention, rather than measures that necessarily capture outcomes that are important for day-to-day delivery in care homes or what care home residents and staff would desire to have measured.

## Conclusions

Care home intervention research is growing, but our review highlights the heterogeneity in outcome assessment and inconsistent use of measures. There is an urgent need to apply outcome measures that are appropriate and sensitive to the care home context, working with residents, family and friends and staff to ensure that research studies are measuring what matters most and in the most efficient and least burdensome way. This requires a collaborative approach to research, with key stakeholders involved from the outset in designing contextually appropriate outcome ascertainment [19].

## Supplementary Material

aa-22-2125-File002_afad069

## Data Availability

Full references for all included studies and all results tables are presented in Supplementary Materials.
