# Supplementary material for: Outcome measures from international older adult care home intervention research: a scoping review
Source: Age Ageing. 2023 May 16;52(5):afad069. doi: 10.1093/ageing/afad069 (PMC10187991; doi:10.1093/ageing/afad069)
Supplement: aa-22-2125-File002_afad069 [file aa-22-2125-file002_afad069.docx]

**Outcome measures from international older adult care home intervention research: a scoping review**

**Supplementary Materials**

| **Appendix 1**: Search strategies | 2 |
| --- | --- |
| **Appendix 2**: Reference list for all included studies | 5 |
| **Appendix 3**: Supplementary Tables 1-4: Descriptive tables for included studies | 37 |
| **Appendix 4**: Supplementary Table 5: Characteristics of included studies table | 40 |
| **Appendix 5**: Supplementary Table 6: Study level reporting of aim, intervention & control | 58 |
| **Appendix 6**: Supplementary Table 7: Complete list of scale-based outcome measures by frequency of use | 99 |

**Appendix 1. Search strategies and grey literature**

**Summary of database searching 2019 & 2022**

| **Database** | **Date searched** | **No. of hits** | **Date searched** | **No. of hits** |
| --- | --- | --- | --- | --- |
| **Ovid MEDLINE** | **25/11/2019** | **4,339** | **11/08/2022** | **1,993** |
| **Ovid EMBASE** | **27/11/2019** | **7,170** | **11/08/2022** | **7,535** |
| **EBSCO Host CINAHL (Cumulative Index to Nursing and Allied Health Literature)** | **2/12/2019** | **5,821** | **11/08/2022** | **2,110** |
| **ProQuest ASSIA (Applied Social Sciences Index and Abstracts)** | **2/12/2019** | **942** | **11/08/2022** | **330** |
| **Total** |  | **18,272** |  | **11,968** |
| **Combined and deduplicated using Endnote** |  | **14,889** |  | **9,303** |
| **Limited to care homes terms* in title and imported into Covidence** |  | **4,789** |  | **1,561** |

***Care homes terms in title – search terms in title**

care home or care homes; residential care; nursing home or nursing homes; long term care or long-term care or longterm care; assisted living; senior living; residential aged care; intermediate care; residential facilities; respite care

**Medline search strategy (adapted for other databases)**

| 1. *Homes for the Aged/ |
| --- |
| 2. *Nursing Homes/ |
| 3. *Long-Term Care/ |
| 4. *Residential Facilities/ |
| 5. *Respite Care/ |
| 6. *Intermediate Care/ |
| 7. "care home$".ab,ti. |
| 8. "nursing home$".ab,ti. |
| 9. "residential care".ab,ti. |
| 10. ("long term care" or "long-term care" or "longterm care").ab,ti. |
| 11. "home$ for the aged".ab,ti. |
| 12. "care facilit*".ab,ti. |
| 13. "old$ people$ home$".ti,ab. |
| 14. (retir$ adj2 home$).ab,ti. |
| 15. ("old$ adult$" adj3 (facilit$ or residential or accommodation)).ab,ti. |
| 16. ("old$ people$" adj3 (facilit$ or residential or accommodation)).ab,ti. |
| 17. ("old$ person$" adj3 (facilit$ or residential or accommodation)).ab,ti. |
| 18. ((geriatric$ or elder$ or senior$ or retir$) adj3 (facilit$ or residential or accommodation)).ab,ti. |
| 19. "respite care".ti,ab. |
| 20. "intermediate care".ti,ab. |
| 21. or/1-20 |
| 22. *Randomized Controlled Trials as Topic/ |
| 23. Randomized controlled trial/ |
| 24. Random allocation/ |
| 25. Double blind method/ |
| 26. Single blind method/ |
| 27. Clinical Trial/ |
| 28. Clinical trials as Topic/ |
| 29. "randomi*ed".ab,ti. |
| 30. randomly.ab,ti. |
| 31. controlled clinical trial.pt. |
| 32. Evaluation Study/ |
| 33. Comparative Study/ |
| 34. "before and after study".ti,ab,mp. |
| 35. or/22-34 |
| 36. 21 and 35 |
| 37. limit 36 to yr="2009 -Current" |

**Grey Literature Search**

**Undertaken 14/11/2019 – 30/01/2020**

**Search terms:** Homes for the Aged, Nursing Homes, Long-Term Care, Residential Facilities, Care Homes, Residential Care, Trial, Intervention, Randomized Controlled Trials, Programme

| ACTC | MedEdPortal |
| --- | --- |
| ADASS | MHRA |
| Age UK | MRC Clinical Trails Unit |
| AGDH | MSAC |
| Alzheimer's Disease International | MERLOT |
| Alzheimer's Prevention Registry | My Aged Care |
| AHRQ | My Home Life |
| Bandolier | National Care Forum |
| Better Caring | National Health Service |
| British Geriatrics Society | Nature Proceedings |
| C5R | NDLTD |
| CADTH | NICA |
| Canadian Institutional Repositories | NICE |
| Care England | NIH |
| Care Home Research Network | NIHR School for Social Care Research |
| Care Home Management | OIAster |
| Care Home UK | OpenDOAR |
| CDC | Open Grey |
| Centre for Reviews and Dissemination | OPHLA |
| ClinicalTrials.gov | OECD |
| CMS | OSF Preprints |
| Cochrane Library | PCORI |
| CQC | PHAC |
| European Clinical Trial Registry | PROSPERO |
| ENRICH | ProQuest |
| European Association of Palliative Care | PSSRU |
| ICER | Public Health England |
| Faculty of Public Health | Public Health Europe |
| Figshare | RCGP |
| Georgetown Medical Review | RIAN |
| Grey Literature Report | SCIE (Social Care Online) |
| Health and Social Care Alliance | Scottish Care |
| Healthcare Improvement Scotland | Scottish Social Services Council |
| Health Evidence | Scottish Dementia Clinical Research Network |
| Health Foundation | SIGN |
| Health Information and Quality Authority | Siren Evidence Library |
| HERU | Social Science Research Group |
| Health Research Authority | THETA |
| Housing & Dementia Research Consortium | Trip |
| Housing & Care For Older People Research Network | UKCRN |
| Institute of Health Economics | UK Department of Health |
| INAHTA | UK Government |
| Independent Age | Think Tank Search |
| Information Services Division Scotland | Virtual Health Library |
| International Longevity Centre | WorldCat |
| Irish Health Repository/Lenus | World Health Organization Health Evidence Network |
| ISRCTN | World Health Organization International Clinical Trials Registry Platform |
| Kings Fund | World Health Organization |
| McMaster University | WorldWideScience |
| McMaster University Health Forum |  |

**Appendix 2: Full Alphabetical Reference List of Included Studies**

1. Abraham J, Kupfer R, Behncke A, Berger-Höger B, Icks A, Haastert B, et al. Implementation of a multicomponent intervention to prevent physical restraints in nursing homes (IMPRINT): A pragmatic cluster randomized controlled trial. International Journal of Nursing Studies. 2019;96:27.

2. Acaroz Candan S, Akoglu AS, Bugusan S, Yuksel F. Effects of neuromuscular electrical stimulation of quadriceps on the quadriceps strength and functional performance in nursing home residents: A comparison of short and long stimulation periods. Geriatrics and Gerontology International. 2019;19(5):409-13.

3. Ailabouni N, Mangin D, Nishtala PS. DEFEAT-polypharmacy: deprescribing anticholinergic and sedative medicines feasibility trial in residential aged care facilities. International Journal of Clinical Pharmacy. 2019;41(1):167-78.

4. Almeida OP, Patel H, Velasquez D, Kelly R, Lai R, Ford AH, et al. Behavioral Activation in Nursing Homes to Treat Depression (BAN-Dep): Results From a Clustered, Randomized, Single-Blinded, Controlled Clinical Trial. American Journal of Geriatric Psychiatry. 2022.

5. Alp FY, Yucel SC. The Effect of Therapeutic Touch on the Comfort and Anxiety of Nursing Home Residents. Journal of Religion & Health. 2021;60(3):2037-50.

6. Álvarez Barbosa F, Alfonso R, del Pozo J. What does the vibration therapy add? A quasiexperimental, pilot study on the short term effects of whole-body vibration as mode of exercise for nursing home residents aged 80+. Journal of Human Sport and Exercise. 2018;13(4):810-22.

7. Anderson K, Wickramariyaratne T, Blair A. A feasibility study of group‐based cognitive behaviour therapy for older adults in residential care. Clinical Psychologist. 2018;22(2):192-202.

8. Arendts G, Deans P, O'Brien K, Etherton-Beer C, Howard K, Lewin G, et al. A clinical trial of nurse practitioner care in residential aged care facilities. Archives of Gerontology & Geriatrics. 2018;77:129-32.

9. Arnold SH, Nygaard Jensen J, Bjerrum L, Siersma V, Winther Bang C, Brostrom Kousgaard M, et al. Effectiveness of a tailored intervention to reduce antibiotics for urinary tract infections in nursing home residents: a cluster, randomised controlled trial. The Lancet Infectious Diseases. 2021;21(11):1549-56.

10. Arrieta H, Rezola-Pardo C, Zarrazquin I, Echeverria I, Yanguas JJ, Iturburu M, et al. A multicomponent exercise program improves physical function in long-term nursing home residents: A randomized controlled trial. Experimental Gerontology. 2018;103:94-100.

11. Arrieta H, Hervás G, Rezola-Pardo C, Ruiz-Litago F, Iturburu M, Yanguas José J, et al. Serum Myostatin Levels Are Higher in Fitter, More Active, and Non-Frail Long-Term Nursing Home Residents and Increase after a Physical Exercise Intervention. Gerontology. 2019;65(3):229-39.

12. Arrieta H, Rezola‐Pardo C, Gil SM, Virgala J, Iturburu M, Antón I, et al. Effects of Multicomponent Exercise on Frailty in Long‐Term Nursing Homes: A Randomized Controlled Trial. Journal of the American Geriatrics Society. 2019;67(6):1145-51.

13. Arrieta H, Rezola-Pardo C, Kortajarena M, Hervás G, Gil J, Yanguas JJ, et al. The impact of physical exercise on cognitive and affective functions and serum levels of brain-derived neurotrophic factor in nursing home residents: A randomized controlled trial. Maturitas. 2020;131:72-7.

14. Arrieta H, Rezola-Pardo C, Gil J, Kortajarena M, Zarrazquin I, Echeverria I, et al. Effects of an individualized and progressive multicomponent exercise program on blood pressure, cardiorespiratory fitness, and body composition in long-term care residents: Randomized controlled trial. Geriatric Nursing. 2022;45:77-84.

15. Ashcraft AS, Owen DC. Comparison of standardized and customized SBAR communication tools to prevent nursing home resident transfer. Applied Nursing Research. 2017;38:64-9.

16. Baandrup L, Jennum PJ. Effect of a dynamic lighting intervention on circadian rest-activity disturbances in cognitively impaired, older adults living in a nursing home: A proof-of-concept study. Neurobiology of sleep and circadian rhythms. 2021;11:100067.

17. Bae S, Asojo AO. Ambient Scent as a Positive Distraction in Long-Term Care Units: Theory of Supportive Design. HERD. 2020;13(4):158-72.

18. Bagci H, Cinar Yucel S. Effect of Therapeutic Touch on Sleep Quality in Elders Living at Nursing Homes. Journal of Religion & Health. 2020;59(3):1304-18.

19. Bailey EM, Stevens AB, LaRocca MA, Scogin F. A Randomized Controlled Trial of a Therapeutic Intervention for Nursing Home Residents With Dementia and Depressive Symptoms. Journal of Applied Gerontology. 2017;36(7):895-908.

20. Baker JR, Webster L, Lynn N, Rogers J, Belcher J. Intergenerational Programs May Be Especially Engaging for Aged Care Residents With Cognitive Impairment: Findings From the Avondale Intergenerational Design Challenge. American Journal of Alzheimer's Disease & Other Dementias. 2017;32(4):213-21.

21. Ballard C, Orrell M, Zhong SY, Moniz-Cook E, Stafford J, Whittaker R, et al. Impact of antipsychotic review and nonpharmacological interventionon antipsychotic use, neuropsychiatric symptoms, and mortality in people with dementia living in nursing homes: A factorial cluster-randomized controlled trial by the well-being and health for people with dementia (WHELD) program. American Journal of Psychiatry. 2016;173(3):252-62.

22. Rajkumar AP, Ballard C, Fossey J, Corbett A, Woods B, Orrell M, et al. Apathy and Its Response to Antipsychotic Review and Nonpharmacological Interventions in People With Dementia Living in Nursing Homes: WHELD, a Factorial Cluster Randomized Controlled Trial. Journal of the American Medical Directors Association. 2016;17(8):741-7.

23. Ballard C, Corbett A, Orrell M, Williams G, Moniz-Cook E, Romeo R, et al. Impact of person-centred care training and person-centred activities on quality of life, agitation, and antipsychotic use in people with dementia living in nursing homes: A cluster-randomised controlled trial. PLoS Medicine. 2018;15(2):e1002500.

24. Balsom C, Pittman N, King R, Kelly D. Impact of a pharmacist-administered deprescribing intervention on nursing home residents: a randomized controlled trial. International journal of clinical pharmacy. 2020;42(4):1153-67.

25. Barbe AG, Kottmann HE, Derman SHM, Noack MJ. Efficacy of regular professional brushing by a dental nurse for 3 months in nursing home residents—A randomized, controlled clinical trial. International Journal of Dental Hygiene. 2019;17(4):327-35.

26. Barbe AG, Kupeli LS, Hamacher S, Noack MJ. Impact of regular professional toothbrushing on oral health, related quality of life, and nutritional and cognitive status in nursing home residents. International journal of dental hygiene. 2020;18(3):238-50.

27. Baron K, Hodgson A, Walshe C. Evaluation of an advance care planning education programme for nursing homes: A Longitudinal study. Nurse Education Today. 2015;35(5):689.

28. Barrett E, Burke M, Whelan S, Santorelli A, Oliveira BL, Cavallo F, et al. Evaluation of a Companion Robot for Individuals With Dementia: Quantitative Findings of the MARIO Project in an Irish Residential Care Setting. Journal of Gerontological Nursing. 2019;45(7):36-45.

29. Barthalos I, Dorgo S, Kopkane Plachy J, Szakaly Z, Ihasz F, Raczne Nemeth T, et al. Randomized controlled resistance training based physical activity trial for central European nursing home residing older adults. Journal of Sports Medicine & Physical Fitness. 2016;56(10):1249-57.

30. Bassi M, Rassiga C, Fumagalli N, Senes G. Quality of experience during horticultural activities: an experience sampling pilot study among older adults living in a nursing home. Geriatric Nursing. 2018;39(4):457-64.

31. Batchelor-Murphy M, Amella EJ, Zapka J, Mueller M, Beck C. Feasibility of a web-based dementia feeding skills training program for nursing home staff. Geriatric Nursing. 2015;36(3):212-8.

32. Bauer M, Rayner JA, Tang J, Koch S, While C, O'Keefe F. An evaluation of Snoezelen() compared to 'common best practice' for allaying the symptoms of wandering and restlessness among residents with dementia in aged care facilities. Geriatric Nursing. 2015;36(6):462-6.

33. Bautrant T, Grino M, Peloso C, Schiettecatte F, Planelles M, Oliver C, et al. Impact of Environmental Modifications to Enhance Day-Night Orientation on Behavior of Nursing Home Residents With Dementia. Journal of the American Medical Directors Association. 2019;20(3):377-81.

34. Beaupre LA, Magaziner JS, Jones CA, Jhangri GS, Johnston DWC, Wilson DM, et al. Rehabilitation After Hip Fracture for Nursing Home Residents: A Controlled Feasibility Trial. Journals of Gerontology Series A: Biological Sciences & Medical Sciences. 2019;74(9):1518-25.

35. Beaupre LA, Lier D, Magaziner JS, Jones CA, Johnston DWC, Wilson DM, et al. An Outreach Rehabilitation Program for Nursing Home Residents After Hip Fracture May Be Cost-Saving. Journals of Gerontology Series A: Biological Sciences & Medical Sciences. 2020;75(10):e159-e65.

36. Beeckman D, Serraes B, Anrys C, Van Tiggelen H, Van Hecke A, Verhaeghe S. A multicentre prospective randomised controlled clinical trial comparing the effectiveness and cost of a static air mattress and alternating air pressure mattress to prevent pressure ulcers in nursing home residents. International Journal of Nursing Studies. 2019;97:105-13.

37. Anrys C, Van Tiggelen H, Verhaeghe S, Van Hecke A, Beeckman D. Independent risk factors for pressure ulcer development in a high‐risk nursing home population receiving evidence‐based pressure ulcer prevention: Results from a study in 26 nursing homes in Belgium. International Wound Journal. 2019;16(2):325-33.

38. Bellini C, Petignat C, Masserey E, Bula C, Burnand B, Rousson V, et al. Universal screening and decolonization for control of MRSA in nursing homes: a cluster randomized controlled study. Infection Control & Hospital Epidemiology. 2015;36(4):401-8.

39. Benigni JP, Uhl JF, Balet F, Filori P, Chahim M. Evaluation of three different devices to reduce stasis edema in poorly mobile nursing home patients. International Angiology. 2018;37(4):322-6.

40. Berkheimer SD, Qian C, Malmstrom TK. Snoezelen Therapy as an Intervention to Reduce Agitation in Nursing Home Patients With Dementia: A Pilot Study. Journal of the American Medical Directors Association. 2017;18(12):1089-91.

41. Bertoncello C, Sperotto M, Bellio S, Pistellato I, Fonzo M, Bigolaro C, et al. Effectiveness of individually tailored exercise on functional capacity and mobility in nursing home residents. British Journal of Community Nursing. 2021;26(3):144-9.

42. Bischoff LL, Cordes T, Meixner C, Schoene D, Voelcker-Rehage C, Wollesen B. Can cognitive-motor training improve physical functioning and psychosocial wellbeing in nursing home residents? A randomized controlled feasibility study as part of the PROCARE project. Aging Clinical & Experimental Research. 2021;33(4):943-56.

43. Blaak J, Kaup O, Hoppe W, Baron-Ruppert G, Langheim H, Staib P, et al. A Long-Term Study to Evaluate Acidic Skin Care Treatment in Nursing Home Residents: Impact on Epidermal Barrier Function and Microflora in Aged Skin. Skin Pharmacology and Physiology. 2015;28(5):269-79.

44. Blair AC, Bird MJ. A pilot trial of psychological therapy groups for the very old in residential care: Clinical and logistical issues. Clinical Psychologist. 2016;20(2):68-79.

45. Blytt KMB, Bjorvatn B, Husebø B, Flo E. Effects of pain treatment on sleep in nursing home patients with dementia and depression – a multicentre placebo-controlled randomised clinical trial. Sleep Medicine. 2017;40:e39-e.

46. Boere TM, van Buul LW, Hopstaken RM, van Tulder MW, Twisk JWMR, Verheij TJM, et al. Effect of C reactive protein point-of-care testing on antibiotic prescribing for lower respiratory tract infections in nursing home residents: cluster randomised controlled trial. BMJ. 2021;374:n2198.

47. Bökberg C, Behm L, Ahlstrom G. Quality of life of older persons in nursing homes after the implementation of a knowledge-based palliative care intervention. International Journal of Older People Nursing. 2019;14(4):e12258.

48. Boockvar KS, Teresi JA, Inouye SK. Preliminary Data: An Adapted Hospital Elder Life Program to Prevent Delirium and Reduce Complications of Acute Illness in Long-Term Care Delivered by Certified Nursing Assistants. Journal of the American Geriatrics Society. 2016;64(5):1108-13.

49. Boockvar KS, Judon KM, Eimicke JP, Teresi JA, Inouye SK. Hospital Elder Life Program in Long-Term Care (HELP-LTC): A Cluster Randomized Controlled Trial. Journal of the American Geriatrics Society. 2020;68(10):2329-35.

50. Boogaard JA, de Vet Henrica CW, van Soest-Poortvliet Mirjam C, Anema JR, Achterberg WP, van der Steen Jenny T. Effects of two feedback interventions on end-of-life outcomes in nursing home residents with dementia: A cluster-randomized controlled three-armed trial. Palliative Medicine. 2018;32(3):693-702.

51. Booth J, Aucott L, Cotton S, Davis B, Fenocchi L, Goodman C, et al. Tibial nerve stimulation compared with sham to reduce incontinence in care home residents: ELECTRIC RCT. Health technology assessment (Winchester, England). 2021;25(41):1-110.

52. Boström G, Conradsson M, Hörnsten C, Rosendahl E, Lindelöf N, Holmberg H, et al. Effects of a high-intensity functional exercise program on depressive symptoms among people with dementia in residential care: a randomized controlled trial. International Journal of Geriatric Psychiatry. 2016;31(8):868-78.

53. Sondell A, Rosendahl E, Sommar JN, Littbrand H, Lundin-Olsson L, Lindelof N. Motivation to participate in high-intensity functional exercise compared with a social activity in older people with dementia in nursing homes. PLoS ONE. 2018;13(11):e0206899.

54. Sondell A, Littbrand H, Holmberg H, Lindelof N, Rosendahl E. Is the Effect of a High-Intensity Functional Exercise Program on Functional Balance Influenced by Applicability and Motivation Among Older People with Dementia in Nursing Homes? Journal of Nutrition, Health & Aging. 2019.

55. Sondell A, Rosendahl E, Gustafson Y, Lindelof N, Littbrand H. The Applicability of a High-Intensity Functional Exercise Program Among Older People With Dementia Living in Nursing Homes. Journal of geriatric physical therapy. 2019;42(4):E16-E24.

56. Toots A, Wiklund R, Littbrand H, Nordin E, Nordström P, Lundin-Olsson L, et al. The Effects of Exercise on Falls in Older People With Dementia Living in Nursing Homes: A Randomized Controlled Trial. Journal of the American Medical Directors Association. 2019;20(7):835-.

57. Bourdon E, Belmin J. Enriched gardens improve cognition and independence of nursing home residents with dementia: a pilot controlled trial. Alzheimer's research & therapy. 2021;13(1):116.

58. Brännström M, Furst CJ, Tishelman C, Petzold M, Lindqvist O. Effectiveness of the Liverpool care pathway for the dying in residential care homes: An exploratory, controlled before-and-after study. Palliative Medicine. 2016;30(1):54-63.

59. Bravo-Jose P, Saez-Lleo CI, Peris-Marti JF. Deprescribing antipsychotics in long term care patients with dementia. Farmacia Hospitalaria. 2019;43(4):140-5.

60. Brazil K, Carter G, Cardwell C, Clarke M, Hudson P, Froggatt K, et al. Effectiveness of advance care planning with family carers in dementia nursing homes: A paired cluster randomized controlled trial. Palliative Medicine. 2018;32(3):603-12.

61. Brett L, Traynor V, Meedya S, Stapley P. Impressions of using the Cohen-Mansfield Agitation Inventory as an outcome measure: Lessons learnt for future clinical researchers (innovative practice). Dementia. 2017 2020/02/01;19(2):464-71.

62. Brett L, Stapley P, Meedya S, Traynor V. Effect of physical exercise on physical performance and fall incidents of individuals living with dementia in nursing homes: a randomized controlled trial. Physiotherapy Theory & Practice. 2019:1-14.

63. Brett L, Stapley P, Meedya S, Traynor V. Effect of physical exercise on physical performance and fall incidents of individuals living with dementia in nursing homes: a randomized controlled trial. Physiotherapy Theory & Practice. 2021;37(1):38-51.

64. Brimelow RE, Thangavelu K, Beattie R, Dissanayaka NN. Feasibility of Group-Based Multiple Virtual Reality Sessions to Reduce Behavioral and Psychological Symptoms in Persons Living in Residential Aged Care. Journal of the American Medical Directors Association. 2022;23(5):831-7.e2.

65. Brodaty H, Aerts L, Harrison F, Jessop T, Cations M, Chenoweth L, et al. Antipsychotic Deprescription for Older Adults in Long-term Care: The HALT Study. Journal of the American Medical Directors Association. 2018;19(7):592-600.e7.

66. Brooker DJ, Latham I, Evans SC, Jacobson N, Perry W, Bray J, et al. FITS into practice: translating research into practice in reducing the use of anti-psychotic medication for people with dementia living in care homes. Aging & Mental Health. 2016;20(7):709-18.

67. Brown KA, Chambers A, MacFarlane S, Langford B, Leung V, Quirk J, et al. Reducing unnecessary urine culturing and antibiotic overprescribing in long-term care: a before-and-after analysis. CMAJ open. 2019;7(1):E174-E81.

68. Brustio PR, Magistro D, Ivaldi S, Caglio MM, Rabaglietti E, Liubicich ME. Neuromotor training in older women living in long-term care setting: A pilot study. Geriatric Nursing. 2015;36(5):361-6.

69. Bryant C, Brown L, Polacsek M, Batchelor F, Capon H, Dow B. Volunteer-led behavioural activation to reduce depression in residential care: a feasibility study. Pilot and feasibility studies. 2020;6:95.

70. Butler CC, Lau M, Gillespie D, Owen-Jones E, Lown M, Wootton M, et al. Effect of Probiotic Use on Antibiotic Administration Among Care Home Residents: A Randomized Clinical Trial. JAMA: Journal of the American Medical Association. 2020;324(1):47-56.

71. Castro-Herrera VM, Fisk HL, Wootton M, Lown M, Owen-Jones E, Lau M, et al. Combination of the Probiotics Lacticaseibacillus rhamnosus GG and Bifidobacterium animalis subsp. lactis, BB-12 Has Limited Effect on Biomarkers of Immunity and Inflammation in Older People Resident in Care Homes: Results From the Probiotics to Reduce Infections iN CarE home reSidentS Randomized, Controlled Trial. Frontiers in immunology. 2021;12:643321.

72. Callegari E, Benth JS, Selbaek G, Gronnerod C, Bergh S. The Effect of the NorGeP-NH on Quality of Life and Drug Prescriptions in Norwegian Nursing Homes: A Randomized Controlled Trial. Pharmacy. 2022;10(1).

73. Cantarella A, Borella E, Faggian S, Navuzzi A, De Beni R. Using dolls for therapeutic purposes: A study on nursing home residents with severe dementia. International Journal of Geriatric Psychiatry. 2018;33(7):915-25.

74. Cateau D, Ballabeni P, Niquille A. Effects of an interprofessional deprescribing intervention in Swiss nursing homes: the Individual Deprescribing Intervention (IDeI) randomised controlled trial. BMC Geriatrics. 2021;21(1):655.

75. Cateau D, Ballabeni P, Niquille A. Effects of an interprofessional Quality Circle-Deprescribing Module (QC-DeMo) in Swiss nursing homes: a randomised controlled trial. BMC Geriatrics. 2021;21(1):289.

76. Catiker A, Zincir H, Sevig EU. The effect of foot care protocol applied to older people on foot health problems and foot care behaviours: a randomised controlled trial in a nursing home. Scandinavian journal of caring sciences. 2021;35(4):1278-89.

77. Cavallini E, Bottiroli S, Capotosto E, De Beni R, Pavan G, Vecchi T, et al. Self-help memory training for healthy older adults in a residential care center: specific and transfer effects on performance and beliefs. International Journal of Geriatric Psychiatry. 2015;30(8):870-80.

78. Cavallini E, Ceccato I, Bertoglio S, Francescani A, Vigato F, Ianes AB, et al. Can theory of mind of healthy older adults living in a nursing home be improved? A randomized controlled trial. Aging Clinical & Experimental Research. 2021;33(11):3029-37.

79. Çetinkaya F, Asiret GD, Direk F, Özkanlı NN. The Effect of Ceramic Painting on the Life Satisfaction and Cognitive Status of Older Adults Residing in a Nursing Home. Topics in Geriatric Rehabilitation. 2019;35(2):108-12.

80. Chalhoub E, Emami E, Freije M, Kandelman D, Campese M, St-Georges A, et al. Effectiveness of an alcohol-free essential oil-containing mouthwash in institutionalised elders receiving long-term care: a feasibility study. Gerodontology. 2016;33(1):69-78.

81. Chambers A, Chen C, Brown KA, Daneman N, Langford B, Leung V, et al. Virtual learning collaboratives to improve urine culturing and antibiotic prescribing in long-term care: controlled before-and-after study. BMJ Quality & Safety. 2022;31(2):94-104.

82. Chiesi F, Gori E, Collini F, Palfrader A, Galli R, Guazzini A, et al. Biodanza as a Nonpharmacological Dance Movement-Based Treatment in Older People With Alzheimer's Disease: An Italian Pilot Study in 2 Tuscan Nursing Homes. Holistic nursing practice. 2021;35(5):264-72.

83. Chou W, Waszynski C, Kessler J, Chiang Y-C, Clarkson P. Using positive images to manage resistance-to-care and combative behaviors in nursing home residents with dementia: A pilot study. Geriatric Nursing. 2016;37(3):215-20.

84. Chu CHL. A feasibility study of a multifaceted walking intervention to maintain functional mobility, ADL function, and quality of life among nursing home residents with dementia 2016.

85. Chu CH, Puts M, Brooks D, Parry M, McGilton KS. A Feasibility Study of a Multifaceted Walking Intervention to Maintain the Functional Mobility, Activities of Daily Living, and Quality of Life of Nursing Home Residents With Dementia. Rehabilitation Nursing. 2020;45(4):204-17.

86. Churcher Clarke A, Chan JMY, Stott J, Royan L, Spector A. An adapted mindfulness intervention for people with dementia in care homes: feasibility pilot study. International Journal of Geriatric Psychiatry. 2017;32(12):e123-e31.

87. Cichocki M, Quehenberger V, Zeiler M, Adamcik T, Manousek M, Stamm T, et al. Effectiveness of a low-threshold physical activity intervention in residential aged care - results of a randomized controlled trial. Clinical Interventions in Aging. 2015;10:885-95.

88. Clarke-O'Neill S, Farbrot A, Lagerstedt ML, Cottenden A, Fader M. An Exploratory Study of Skin Problems Experienced by UK Nursing Home Residents Using Different Pad Designs. Journal of Wound, Ostomy, & Continence Nursing. 2015;42(6):621-31; quiz E1.

89. Cohen SMBA, Volandes AEMDMPH, Shaffer MLP, Hanson LCMDMPH, Habtemariam DBA, Mitchell SLMDMPH. Concordance Between Proxy Level of Care Preference and Advance Directives Among Nursing Home Residents With Advanced Dementia: A Cluster Randomized Clinical Trial. Journal of Pain and Symptom Management. 2019;57(1):37.

90. Colón-Emeric CS, Corazzini K, McConnell ES, Pan W, Toles M, Hall R, et al. Effect of Promoting High-Quality Staff Interactions on Fall Prevention in Nursing Homes: A Cluster-Randomized Trial. JAMA Intern Med. 2017 Nov 1;177(11):1634-41.

91. Connolly MJ, Boyd M, Broad JB, Kerse N, Lumley T, Whitehead N, et al. The Aged Residential Care Healthcare Utilization Study (ARCHUS): A Multidisciplinary, Cluster Randomized Controlled Trial Designed to Reduce Acute Avoidable Hospitalizations From Long-Term Care Facilities. Journal of the American Medical Directors Association. 2015 2015/01/01/;16(1):49-55.

92. Connolly MJ, Broad JB, Boyd M, Zhang TX, Kerse N, Foster S, et al. The 'Big Five'. Hypothesis generation: a multidisciplinary intervention package reduces disease-specific hospitalisations from long-term care: a post hoc analysis of the ARCHUS cluster-randomised controlled trial. Age and Ageing. 2016;45(3):415-20.

93. Conway J, Higgins I, Hullick C, Hewitt J, Dilworth S. Nurse-led ED support for Residential Aged Care Facility staff: An evaluation study. International Emergency Nursing. 2015;23(2):190-6.

94. Cool C, Cestac P, McCambridge C, Rouch L, de Souto Barreto P, Rolland Y, et al. Reducing potentially inappropriate drug prescribing in nursing home residents: effectiveness of a geriatric intervention. Br J Clin Pharmacol. 2018 Jul;84(7):1598-610.

95. Cordato NJ, Kearns M, Smerdely P, Seeher KM, Gardiner MD, Brodaty H. Management of Nursing Home Residents Following Acute Hospitalization: Efficacy of the "Regular Early Assessment Post-Discharge (REAP)" Intervention. Journal of the American Medical Directors Association. 2018;19(3):276.e11-.e19.

96. Cordes T, Zwingmann K, Rudisch J, Voelcker-Rehage C, Wollesen B. Multicomponent exercise to improve motor functions, cognition and well-being for nursing home residents who are unable to walk - A randomized controlled trial. Experimental gerontology. 2021;153:111484.

97. Couderc AL, Berbis J, Delalande G, Mugnier B, Courcier A, Bourriquen M, et al. Impact of care pathway for nursing home residents treated for cancer: ONCO-EHPAD study. Supportive Care in Cancer. 2021;29(7):3933-42.

98. Courel-Ibanez J, Buendia-Romero A, Pallares JG, Garcia-Conesa S, Martinez-Cava A, Izquierdo M. Impact of Tailored Multicomponent Exercise for Preventing Weakness and Falls on Nursing Home Residents' Functional Capacity. Journal of the American Medical Directors Association. 2022;23(1):98-104.e3.

99. Crespy SD, Van Haitsma K, Kleban M, Hann CJ. Reducing Depressive Symptoms in Nursing Home Residents: Evaluation of the Pennsylvania Depression Collaborative Quality Improvement Program. Journal for Healthcare Quality: Promoting Excellence in Healthcare. 2016;38(6):e76-e88.

100. Croonquist CG, Dalum J, Skott P, Sjogren P, Wardh I, Moren E. Effects of domiciliary professional oral care for care-dependent elderly in nursing homes - oral hygiene, gingival bleeding, root caries and nursing staff's oral health knowledge and attitudes. Clinical Interventions in Aging. 2020;15:1305-15.

101. Daneman N, Lee SM, Bai H, Bell CM, Bronskill SE, Campitelli MA, et al. Population-Wide Peer Comparison Audit and Feedback to Reduce Antibiotic Initiation and Duration in Long-Term Care Facilities with Embedded Randomized Controlled Trial. Clinical Infectious Diseases. 2021;73(6):e1296-e304.

102. Davidson C, Loganathan S, Bishop L, Imhof L, Bergofsky L, Spector W, et al. Scalability of an IT Intervention to Prevent Pressure Ulcers in Nursing Homes. Journal of the American Medical Directors Association. 2019;20(7):816-21.e2.

103. Davison TE, Eppingstall B, Runci S, O'Connor DW. A pilot trial of acceptance and commitment therapy for symptoms of depression and anxiety in older adults residing in long-term care facilities. Aging & Mental Health. 2017;21(7):766-73.

104. Davison TE, McCabe MP, Busija L, Graham A, Camoes-Costa V, Kelly J, et al. The effectiveness of the Program to Enhance Adjustment to Residential Living (PEARL) in reducing depression in newly admitted nursing home residents. Journal of affective disorders. 2021;282:1067-75.

105. de Bruin ED, Baur H, Brulhart Y, Luijckx E, Hinrichs T, Rogan S. Combining Stochastic Resonance Vibration With Exergaming for Motor-Cognitive Training in Long-Term Care; A Sham-Control Randomized Controlled Pilot Trial. Frontiers in medicine. 2020;7:507155.

106. De Luca R, Bramanti A, De Cola MC, Leonardi S, Torrisi M, Aragona B, et al. Cognitive training for patients with dementia living in a sicilian nursing home: a novel web-based approach. Neurological Sciences. 2016;37(10):1685-91.

107. De Luca R, Bramanti A, De Cola MC, Trifiletti A, Tomasello P, Torrisi M, et al. Tele-health-care in the elderly living in nursing home: the first Sicilian multimodal approach. Aging Clinical & Experimental Research. 2016;28(4):753-9.

108. de Pooter-Stijnman LMM, Vrijkotte S, Smalbrugge M. Effect of caffeine on sleep and behaviour in nursing home residents with dementia. European Geriatric Medicine. 2018;9(6):829-35.

109. de Souto Barreto P, Cesari M, Denormandie P, Armaingaud D, Vellas B, Rolland Y. Exercise or Social Intervention for Nursing Home Residents with Dementia: A Pilot Randomized, Controlled Trial. Journal of the American Geriatrics Society. 2017;65(9):E123-E9.

110. Del Campo Cervantes JM, Macias Cervantes MH, Monroy Torres R. Effect of a Resistance Training Program on Sarcopenia and Functionality of the Older Adults Living in a Nursing Home. Journal of Nutrition, Health & Aging. 2019;23(9):829-36.

111. Dellinger JK, Pitzer S, Schaffler-Schaden D, Schreier MM, Fahrmann LS, Hempel G, et al. Improving medication appropriateness in nursing homes via structured interprofessional medication-review supported by health information technology: a non-randomized controlled study. BMC Geriatrics. 2020;20(1):506.

112. Demir F, Sahin M, Ergezen G. Effectiveness of Proprioceptive Exercise on Balance and Sensory Function in Nursing Home Geriatric Individuals with Diabetes: Randomized Controlled Trial. Turkiye Klinikleri Journal of Medical Sciences. 2022;42(1):27-34.

113. Desborough JA, Clark A, Houghton J, Sach T, Shaw V, Kirthisingha V, et al. Clinical and cost effectiveness of a multi-professional medication reviews in care homes (CAREMED). International journal of pharmacy practice. 2020;28(6):626-34.

114. Devereaux T. Condition-specific SBAR use in long term care and its effect on nurse perception of nurse-physician communication and acute care hospitalizations: A pilot study 2016.

115. Dharmarajan TS, Mahajan D, Zambrano A, Agarwal B, Fischer R, Sheikh Z, et al. Sliding Scale Insulin vs Basal-Bolus Insulin Therapy in Long-Term Care: A 21-Day Randomized Controlled Trial Comparing Efficacy, Safety and Feasibility. Journal of the American Medical Directors Association. 2016;17(3):206-13.

116. Diegelmann M, Jansen C-P, Wahl H-W, Schilling OK, Schnabel E-L, Hauer K. Does a physical activity program in the nursing home impact on depressive symptoms? A generalized linear mixed-model approach. Aging & Mental Health. 2018;22(6):784-93.

117. Dimori S, Leoni G, Fior L, Gasparotto F. Clinical nutrition and physical rehabilitation in a long-term care setting: preliminary observations in sarcopenic older patients. Aging Clinical & Experimental Research. 2018;30(8):951-8.

118. Dolu I, Nahcivan NO. Impact of a nurse‐led sleep programme on the sleep quality and depressive symptomatology among older adults in nursing homes: A non‐randomised controlled study. International Journal of Older People Nursing. 2019;14(1).

119. Dowson L, Marshall C, Buising K, Friedman ND, Kong DCM, Stuart RL. Optimizing treatment of respiratory tract infections in nursing homes: Nurse-initiated polymerase chain reaction testing. American Journal of Infection Control. 2019;47(8):911-5.

120. Dräger D, Budnick A, Kuhnert R, Kalinowski S, Könner F, Kreutz R. Pain management intervention targeting nursing staff and general practitioners: Pain intensity, consequences and clinical relevance for nursing home residents. Geriatr Gerontol Int. 2017 Oct;17(10):1534-43.

121. Drotningsvik A, Oterhals A, Flesland O, Nygard O, Gudbrandsen OA. Fish protein supplementation in older nursing home residents: a randomised, double-blind, pilot study. Pilot and feasibility studies. 2019;5:35.

122. Dugre N, Bell JS, Hopkins RE, Ilomaki J, Chen EYH, Corlis M, et al. Impact of Medication Regimen Simplification on Medication Incidents in Residential Aged Care: SIMPLER Randomized Controlled Trial. Journal of clinical medicine. 2021;10(5).

123. Duru Asiret G. Effect of reminiscence therapy on the sleep quality of the elderly living in nursing homes: A randomized clinical trial. European Journal of Integrative Medicine. 2018;20:1-5.

124. Düzgün G, Durmaz Akyol A. Effect of Natural Sunlight on Sleep Problems and Sleep Quality of the Elderly Staying in the Nursing Home. Holistic Nursing Practice. 2017;31(5):295-302.

125. El Alili M, Smaling HJA, Joling KJ, Achterberg WP, Francke AL, Bosmans JE, et al. Cost-effectiveness of the Namaste care family program for nursing home residents with advanced dementia in comparison with usual care: a cluster-randomized controlled trial. BMC Health Services Research. 2020;20(1):831.

126. El Haddad K, de Souto Barreto P, de Mazieres CL, Rolland Y. Effect of a geriatric intervention aiming to improve polypharmacy in nursing homes. European Geriatric Medicine. 2020;11(5):863-8.

127. Ellis JM, Wells Y, Ong JSM. Non-Pharmacological Approaches to Pain Management in Residential Aged Care: a Pre-Post-Test Study. Clinical Gerontologist. 2019;42(3):286-96.

128. Ercan-Sahin N, Nuran Emiroglu O. Quality of life of nursing home residents before and after reminiscence therapy. Educational Gerontology. 2018;44(2/3):99-107.

129. Erdal A, Flo E, Aarsland D, Ballard C, Slettebo DD, Husebø BS. Efficacy and Safety of Analgesic Treatment for Depression in People with Advanced Dementia: Randomised, Multicentre, Double-Blind, Placebo-Controlled Trial (DEP.PAIN.DEM). Drugs & Aging. 2018 Jun;35(6):545-58.

130. Erdal A, Flo E, Aarsland D, Selbaek G, Ballard C, Slettebo DD, et al. Tolerability of buprenorphine transdermal system in nursing home patients with advanced dementia: a randomized, placebo-controlled trial (DEP.PAIN.DEM). Clinical Interventions in Aging. 2018;13:935-46.

131. Ergin E, Çinar Yücel Ş. The Effect of Music on the Comfort and Anxiety of Older Adults Living in a Nursing Home in Turkey. Journal of Religion & Health. 2019;58(4):1401-14.

132. Eriksson K, Forsgren E, Hartelius L, Saldert C. Communication partner training of enrolled nurses working in nursing homes with people with communication disorders caused by stroke or Parkinson’s disease. Disability & Rehabilitation. 2016;38(12):1187-203.

133. Ersek M, Neradilek MB, Herr K, Jablonski A, Polissar N, Du Pen A. Pain Management Algorithms for Implementing Best Practices in Nursing Homes: Results of a Randomized Controlled Trial. Journal of the American Medical Directors Association. 2016 Apr 1;17(4):348-56.

134. Evrard P, Henrard S, Foulon V, Spinewine A. Benzodiazepine Use and Deprescribing in Belgian Nursing Homes: Results from the COME-ON Study. Journal of the American Geriatrics Society. 2020;68(12):2768-77.

135. Farlow MR, Borson S, Connor SR, Grossberg GT, Mittelman MS. Quality Improvement in Skilled Nursing Facilities for Residents with Alzheimer's Disease. American Journal of Alzheimer's Disease and other Dementias. 2016;31(2):156-62.

136. Fjeld KG, Eide H, Mowe M, Sandvik L, Willumsen T. A 1-year follow-up of a randomized clinical trial with focus on manual and electric toothbrushes' effect on dental hygiene in nursing homes. Acta Odontologica Scandinavica. 2018;76(4):257-61.

137. Folkerts A-K, Dorn ME, Roheger M, Maassen M, Koerts J, Tucha O, et al. Cognitive Stimulation for Individuals with Parkinson's Disease Dementia Living in Long-Term Care: Preliminary Data from a Randomized Crossover Pilot Study. Parkinson's Disease. 2018:1-9.

138. Forbat L, Liu W-M, Koerner J, Lam L, Samara J, Chapman M, et al. Reducing time in acute hospitals: A stepped-wedge randomised control trial of a specialist palliative care intervention in residential care homes. Palliative Medicine. 2020;34(5):571-9.

139. Forster A, Airlie J, Ellwood A, Godfrey M, Green J, Cundill B, et al. intervention to increase physical activity in care home residents: results of a cluster-randomised, controlled feasibility trial (the REACH trial). Age and Ageing. 2021;50(6):2063-78.

140. Frändin K, Grönstedt H, Helbostad JL, Bergland A, Andresen M, Puggaard L, et al. Long-Term Effects of Individually Tailored Physical Training and Activity on Physical Function, Well-Being and Cognition in Scandinavian Nursing Home Residents: A Randomized Controlled Trial. Gerontology. 2016;62(6):571-80.

141. Froggatt K, Best A, Bunn F, Burnside G, Coast J, Dunleavy L, et al. A group intervention to improve quality of life for people with advanced dementia living in care homes: the Namaste feasibility cluster RCT. Health technology assessment (Winchester, England). 2020;24(6):1-140.

142. Gajewska D, Gosa P, Keszycka PK. Dietary Intervention Effectiveness, Clinical Outcomes and Nutrient and Salicylate Intakes in Older Adults Living in Long-Term Care Homes: The Results from the Senior's Plate Project. Nutrients. 2022;14(4).

143. Galik EM, Resnick B, Holmes SD, Vigne E, Lynch K, Ellis J, et al. A Cluster Randomized Controlled Trial Testing the Impact of Function and Behavior Focused Care for Nursing Home Residents With Dementia. Journal of the American Medical Directors Association. 2021;22(7):1421-8.e4.

144. Garland A, Keller H, Quail P, Boscart V, Heyer M, Ramsey C, et al. BABEL (Better tArgeting, Better outcomes for frail ELderly patients) advance care planning: a comprehensive approach to advance care planning in nursing homes: a cluster randomised trial. Age and Ageing. 2022;51(3).

145. Garland CT, Guénette L, Kröger E, Carmichael P-H, Rouleau R, Sirois C. A New Care Model Reduces Polypharmacy and Potentially Inappropriate Medications in Long-Term Care. Journal of the American Medical Directors Association. 2021;22(1):141-7.

146. Gattinger H, Hantikainen V, Ott S, Stark M. Effectiveness of a mobility monitoring system included in the nursing care process in order to enhance the sleep quality of nursing home residents with cognitive impairment. Health and Technology. 2017;7(2-3):161-71.

147. Gemelli MG, Yockel K, Hohmeier KC. Evaluating the Impact of Pharmacists on Reducing Use of Sedative/Hypnotics for Treatment of Insomnia in Long-Term Care Facility Residents. Consultant Pharmacist. 2016;31(11):650-7.

148. Gencbas D, Bebis H, Cicek H. Evaluation of the Efficiency of the Nursing Care Plan Applied Using NANDA, NOC, and NIC Linkages to Elderly Women with Incontinence Living in a Nursing Home: A Randomized Controlled Study. International Journal Of Nursing Knowledge. 2018;29(4):217-26.

149. Gillis K, Tency I, Roelant E, Laureys S, Devriendt H, Lips D. Skin hydration in nursing home residents using disposable bed baths. Geriatric Nursing. 2016;37(3):175.

150. Gillis K, Lahaye H, Dom S, Lips D, Arnouts H, Van Bogaert P. A person‐centred team approach targeting agitated and aggressive behaviour amongst nursing home residents with dementia using the Senses Framework. International Journal of Older People Nursing. 2019;14(4):N.PAG-N.PAG.

151. Gine-Garriga M, Dall PM, Sandlund M, Jerez-Roig J, Chastin SFM, Skelton DA. A Pilot Randomised Clinical Trial of a Novel Approach to Reduce Sedentary Behaviour in Care Home Residents: Feasibility and Preliminary Effects of the GET READY Study. International journal of environmental research and public health. 2020;17(8).

152. Gök Ugur H, Yaman Aktaş Y, Orak OS, Saglambilen O, Aydin Avci İ. The effect of music therapy on depression and physiological parameters in elderly people living in a Turkish nursing home: a randomized-controlled trial. Aging & Mental Health. 2017;21(12):1280-6.

153. Gordon SE, Dufour AB, Monti SM, Mattison MLP, Catic AG, Thomas CP, et al. Impact of a Videoconference Educational Intervention on Physical Restraint and Antipsychotic Use in Nursing Homes: Results From the ECHO-AGE Pilot Study. Journal of the American Medical Directors Association. 2016;17(6):553-6.

154. Goyal AR, Engedal K, Benth JS, Strom BS. Effects of the Sonas Program on Anxiety and Depression in Nursing Home Residents with Dementia: A 6-Month Randomized Controlled Trial. Dementia & Geriatric Cognitive Disorders Extra. 2021;11(2):151-8.

155. Graham L, Ellwood A, Hull K, Fisher J, Cundill B, Holland M, et al. A posture and mobility training package for care home staff: results of a cluster randomised controlled feasibility trial (the PATCH trial). Age and Ageing. 2020;49(5):821-8.

156. Gravenstein S, Davidson HE, Taljaard M, Ogarek J, Gozalo P, Han L, et al. Comparative effectiveness of high-dose versus standard-dose influenza vaccination on numbers of US nursing home residents admitted to hospital: a cluster-randomised trial. The Lancet Respiratory Medicine. 2017;5(9):738-46.

157. Gravenstein S, McConeghy KW, Saade E, Davidson HE, Canaday DH, Han L, et al. Adjuvanted Influenza Vaccine and Influenza Outbreaks in US Nursing Homes: Results From a Pragmatic Cluster-Randomized Clinical Trial. Clinical Infectious Diseases. 2021;73(11):e4229-e36.

158. Guion V, De Souto Barreto P, Sourdet S, Rolland Y. Effect of an Educational and Organizational Intervention on Pain in Nursing Home Residents: A Nonrandomized Controlled Trial. Journal of the American Medical Directors Association. 2018;19(12):1118-.

159. Gulla C, Flo E, Kjome RLS, Husebø BS. Deprescribing antihypertensive treatment in nursing home patients and the effect on blood pressure. Journal of Geriatric Cardiology. 2018;15(4):275-83.

160. Gunst M, De Meyere I, Willems H, Schoenmakers B. Effect of exergaming on wellbeing of residents in a nursing home: a single blinded intervention study. Aging Clinical & Experimental Research. 2022;34(1):151-7.

161. Gustavsson J, Bonander C, Andersson R, Nilson F. Investigating the fall-injury reducing effect of impact absorbing flooring among female nursing home residents: initial results. Injury Prevention. 2015;21(5):320-4.

162. Guzmán A, Freeston M, Rochester L, Hughes JC, James IA. Psychomotor Dance Therapy Intervention (DANCIN) for people with dementia in care homes: A multiple-baseline single-case study. International Psychogeriatrics. 2016;28(10):1695-715.

163. Haddad KE, de Souto Barreto P, Gerard S, Prouff A, Vellas B, Rolland Y. Effect of Relocation on Neuropsychiatric Symptoms in Elderly Adults Living in Long-Term Care. Journal of the American Geriatrics Society. 2018;66(11):2183-7.

164. Hahnel E, Blume-Peytavi U, Trojahn C, Dobos G, Stroux A, Garcia Bartels N, et al. The effectiveness of standardized skin care regimens on skin dryness in nursing home residents: A randomized controlled parallel-group pragmatic trial. International Journal of Nursing Studies. 2017;70:1-10.

165. Haines TP, Palmer AJ, Tierney P, Si L, Robinson AL. A new model of care and in-house general practitioners for residential aged care facilities: a stepped wedge, cluster randomised trial. The Medical journal of Australia. 2020;212(9):409-15.

166. Halek M, Reuther S, Muller-Widmer R, Trutschel D, Holle D. Dealing with the behaviour of residents with dementia that challenges: A stepped-wedge cluster randomized trial of two types of dementia-specific case conferences in nursing homes (FallDem). International journal of nursing studies. 2020;104:103435.

167. Hanlon JT, Perera S, Schweon S, Drinka P, Crnich C, Nace DA. Improvements in Antibiotic Appropriateness for Cystitis in Older Nursing Home Residents: A Quality Improvement Study With Randomized Assignment. Journal of the American Medical Directors Association. 2021;22(1):173-7.

168. Hansen KV, Froiland CT, Testad I. Porcelain for All - a nursing home study. International Journal of Health Care Quality Assurance. 2018;31(7):662-75.

169. Harrison TC, Blozis SA, Schmidt B, Johnson A, Moreno R, Mead S, et al. Music Compared with Auditory Books: A Randomized Controlled Study Among Long-Term Care Residents with Alzheimer's Disease or Related Dementia. Journal of the American Medical Directors Association. 2021;22(7):1415-20.

170. Hartshorn JE, Cowen HJ, Comnick CL. Cluster randomized control trial of nursing home residents' oral hygiene following the Mouth Care Matters education program for certified nursing assistants. Special care in dentistry. 2021;41(3):372-80.

171. Hashimoto M, Kato S, Tanabe Y, Katakura M, Mamun AA, Ohno M, et al. Beneficial effects of dietary docosahexaenoic acid intervention on cognitive function and mental health of the oldest elderly in Japanese care facilities and nursing homes. Geriatrics & gerontology international. 2017;17(2):330-7.

172. Hassan BH, Hewitt J, Keogh JW, Bermeo S, Duque G, Henwood TR. Impact of resistance training on sarcopenia in nursing care facilities: A pilot study. Geriatric Nursing. 2016 Mar-Apr;37(2):116-21.

173. Henskens M, Nauta IM, Drost KT, Scherder EJA. The effects of movement stimulation on activities of daily living performance and quality of life in nursing home residents with dementia: A randomized controlled trial. Clinical Interventions in Aging. 2018;13:805-17.

174. Henskens M, Nauta Ilse M, van Eekeren Marieke CA, Scherder Erik JA. Effects of Physical Activity in Nursing Home Residents with Dementia: A Randomized Controlled Trial. Dementia & Geriatric Cognitive Disorders. 2018;46(1/2):60-80.

175. Henwood T, Neville C, Baguley C, Clifton K, Beattie E. Physical and functional implications of aquatic exercise for nursing home residents with dementia. Geriatric Nursing. 2015;36(1):35-9.

176. Hermans K, De Almeida Mello J, Spruytte N, Cohen J, Van Audenhove C, Declercq A. Does using the interRAI Palliative Care instrument reduce the needs and symptoms of nursing home residents receiving palliative care? Palliative & Supportive Care. 2018;16(1):32-40.

177. Hewitt J, Goodall S, Clemson L, Henwood T, Refshauge K. Progressive Resistance and Balance Training for Falls Prevention in Long-Term Residential Aged Care: A Cluster Randomized Trial of the Sunbeam Program. Journal of the American Medical Directors Association. 2018;19(4):361-9.

178. Hirdes JP, Major J, Didic S, Quinn C, Mitchell L, Chen J, et al. A Canadian Cohort Study to Evaluate the Outcomes Associated with a Multicenter Initiative to Reduce Antipsychotic Use in Long-Term Care Homes. Journal of the American Medical Directors Association. 2020;21(6):817-22.

179. Hjetland GJ, Kolberg E, Pallesen S, Thun E, Nordhus IH, Bjorvatn B, et al. Ambient bright light treatment improved proxy-rated sleep but not sleep measured by actigraphy in nursing home patients with dementia: a placebo-controlled randomised trial. BMC Geriatrics. 2021;21(1):312.

180. Hmwe NTT, Browne G, Mollart L, Allanson V, Chan SWC. The Effect of Acupressure on Sleep Quality of Older People in Australian Residential Aged Care Facilities: A Pilot Randomized Controlled Trial. Holistic nursing practice. 2022;36(4):232-46.

181. Hockley J. A practice development initiative supporting care home staff deliver high quality end-of-life care. International Journal of Palliative Nursing. 2016;22(10):474-81.

182. Hodl M, Halfens RJG, Lohrmann C. Effectiveness of conservative urinary incontinence management among female nursing home residents-A cluster RCT. Archives of Gerontology & Geriatrics. 2019;81:245-51.

183. Hollaar VRY, van der Putten GJ, van der Maarel-Wierink CD, Bronkhorst EM, de Swart BJM, Creugers NHJ. The effect of a daily application of a 0.05% chlorhexidine oral rinse solution on the incidence of aspiration pneumonia in nursing home residents: a multicenter study. BMC Geriatrics. 2017;17(1):128.

184. Hopkins S, Morgan PL, Schlangen LJM, Williams P, Skene DJ, Middleton B. Blue-Enriched Lighting for Older People Living in Care Homes: Effect on Activity, Actigraphic Sleep, Mood and Alertness. Current Alzheimer Research. 2017;14(10):1053-62.

185. Hopper T, Slaughter SE, Hodgetts B, Ostevik A, Ickert C. Hearing Loss and Cognitive-Communication Test Performance of Long-Term Care Residents With Dementia: Effects of Amplification. Journal of speech, language, and hearing research. 2016;59(6):1533-42.

186. Huey-Ming T, Jansen LS, Okpalauwaekwe U, Khasnabish S, Andreas B, Dykes PC. Adopting the Fall Tailoring Interventions for Patient Safety (TIPS) Program to Engage Older Adults in Fall Prevention in a Nursing Home. Journal of Nursing Care Quality. 2021;36(4):327-32.

187. Hullick C, Conway J, Higgins I, Hewitt J, Dilworth S, Holliday E, et al. Emergency department transfers and hospital admissions from residential aged care facilities: a controlled pre-post design study. BMC Geriatrics. 2016;16:102.

188. Hullick C, Conway J, Hall A, Murdoch W, Cole J, Hewitt J, et al. Video-telehealth to support clinical assessment and management of acutely unwell older people in Residential Aged Care: a pre-post intervention study. BMC Geriatrics. 2022;22(1):1-9.

189. Hurley MV, Wood J, Smith R, Grant R, Jordan J, Gage H, et al. The feasibility of increasing physical activity in care home residents: Active Residents in Care Homes (ARCH) programme. Physiotherapy. 2020;107:50-7.

190. Husebø BS, Ballard C, Aarsland D, Selbaek G, Slettebo DD, Gulla C, et al. The Effect of a Multicomponent Intervention on Quality of Life in Residents of Nursing Homes: A Randomized Controlled Trial (COSMOS). Journal of the American Medical Directors Association. 2019;20(3):330-9.

191. Gedde MH, Husebø BS, Mannseth J, Kjome RLS, Naik M, Berge LI. Less Is More: The Impact of Deprescribing Psychotropic Drugs on Behavioral and Psychological Symptoms and Daily Functioning in Nursing Home Patients. Results From the Cluster-Randomized Controlled COSMOS Trial. American Journal of Geriatric Psychiatry. 2021;29(3):304-15.

192. Habiger TF, Achterberg WP, Flo-Groeneboom E, Mannseth J, Husebø BS. Managing Pain and Psychosis Symptoms in Nursing Home Patients: Results from a Cluster-Randomized Controlled Trial (COSMOS). Journal of the American Medical Directors Association. 2021;22(8):1692-8.

193. Inch J, Notman F, Bond CM, Alldred DP, Arthur A, Blyth A, et al. The Care Home Independent Prescribing Pharmacist Study (CHIPPS)-a nonrandomised feasibility study of independent pharmacist prescribing in care homes. Pilot and feasibility studies. 2019;5(1):89.

194. Isaac V, Kuot A, Hamiduzzaman M, Strivens E, Greenhill J. The outcomes of a person-centered, non-pharmacological intervention in reducing agitation in residents with dementia in Australian rural nursing homes. BMC Geriatrics. 2021;21(1):193.

195. Parajuli DR, Kuot A, Hamiduzzaman M, Gladman J, Isaac V. Person-centered, non-pharmacological intervention in reducing psychotropic medications use among residents with dementia in Australian rural aged care homes. BMC Psychiatry. 2021;21(1):36.

196. Iuliano S, Poon S, Robbins J, Bui M, Wang X, De Groot L, et al. Effect of dietary sources of calcium and protein on hip fractures and falls in older adults in residential care: cluster randomised controlled trial. BMJ. 2021;375:n2364.

197. Jablonski RA, Kolanowski AM, Azuero A, Winstead V, Jones-Townsend C, Geisinger ML. Randomised clinical trial: Efficacy of strategies to provide oral hygiene activities to nursing home residents with dementia who resist mouth care. Gerodontology. 2018 Dec;35(4):365-75.

198. Jahanpeyma P, Kayhan Kocak FO, Yildirim Y, Sahin S, Senuzun Aykar F. Effects of the Otago exercise program on falls, balance, and physical performance in older nursing home residents with high fall risk: a randomized controlled trial. European Geriatric Medicine. 2021;12(1):107-15.

199. Jansen CP, Diegelmann M, Schilling OK, Werner C, Schnabel EL, Wahl HW, et al. Pushing the Boundaries: A Physical Activity Intervention Extends Sensor-Assessed Life-Space in Nursing Home Residents. Gerontologist. 2018;58(5):979-88.

200. Jarboe DE. The Effect of Evaluating a Quality Improvement Initiative on Reducing Hospital Transfers of Nursing Home Residents: Walden University; 2015.

201. Jeong E, Chang SO. Can multifaceted educational intervention improve clinical practice and patient outcomes regarding delirium in nursing homes? Japan Journal of Nursing Science. 2022;19(1):1-10.

202. Johansson I, Torgé CJ, Lindmark U. Is an oral health coaching programme a way to sustain oral health for elderly people in nursing homes? A feasibility study. International journal of dental hygiene. 2020;18(1):107-15.

203. Johnen B, Schott N. Feasibility of a machine vs free weight strength training program and its effects on physical performance in nursing home residents: a pilot study. Aging Clinical & Experimental Research. 2018;30(7):819-28.

204. Jøranson N, Olsen C, Calogiuri G, Ihlebaek C, Pedersen I. Effects on sleep from group activity with a robotic seal for nursing home residents with dementia: a cluster randomized controlled trial. International Psychogeriatrics. 2021;33(10):1045-56.

205. Jordan S, Gabe-Walters ME, Watkins A, Humphreys I, Newson L, Snelgrove S, et al. Nurse-Led Medicines' Monitoring for Patients with Dementia in Care Homes: A Pragmatic Cohort Stepped Wedge Cluster Randomised Trial. PLoS ONE. 2015;10(10):e0140203.

206. Jordan KJ, Pao-Feng T, Seongkum H, Shasha B, Dailey D, Beck CK, et al. Pilot Testing a Coaching Intervention to Improve Certified Nursing Assistants' Dressing of Nursing Home Residents. Research in Gerontological Nursing. 2017;10(6):267-76.

207. Jung JY, Park SY, Kim JK. The effects of a client-centered leisure activity program on satisfaction, self-esteem, and depression in elderly residents of a long-term care facility. J Phys Ther Sci. 2018 Jan;30(1):73-6.

208. Junius-Walker U, Krause O, Thurmann P, Bernhard S, Fuchs A, Sparenberg L, et al. Drug Safety for Nursing-Home Residents-Findings of a Pragmatic, Cluster-Randomized, Controlled Intervention Trialin 44 Nursing Homes. Deutsches Arzteblatt international. 2021;118(42):705-12.

209. Jupiter T. Does Hearing Assistive Technology Provide Benefit to Nursing Home Residents with Dementia? A Pilot Study. Journal of the Academy of Rehabilitative Audiology. 2016;49:34-9.

210. Juthani-Mehta M, Van Ness PH, Bianco L, Rink A, Rubeck S, Ginter S, et al. Effect of Cranberry Capsules on Bacteriuria Plus Pyuria Among Older Women in Nursing Homes: A Randomized Clinical Trial. JAMA. 2016;316(18):1879-87.

211. Kalinowski S, Drager D, Kuhnert R, Kreutz R, Budnick A. Pain, Fear of Falling, and Functional Performance Among Nursing Home Residents: A Longitudinal Study. Western Journal of Nursing Research. 2019;41(2):191-216.

212. Kalinowski S, Budnick A, Kuhnert R, Könner F, Kissel-Kröll A, Kreutz R, et al. Nonpharmacologic Pain Management Interventions in German Nursing Homes: A Cluster Randomized Trial. Pain Management Nursing. 2015;16(4):464-74.

213. Kalu ME, Dal Bello-Haas V, Hadjistavropoulos T, Thorpe L, Griffin M, Ploeg J, et al. The Effects of a Walking Intervention on Gait Parameters in Older Adults Residing in Long-Term Care: A Randomized Controlled Trial. Journal of Nutrition, Health & Aging. 2021;25(9):1099-105.

214. Kane RL, Huckfeldt P, Tappen R, Engstrom G, Rojido C, Newman D, et al. Effects of an Intervention to Reduce Hospitalizations From Nursing Homes: A Randomized Implementation Trial of the INTERACT Program. JAMA Internal Medicine. 2017;177(9):1257-64.

215. Kårefjärd A, Nordgren L. Effects of dog-assisted intervention on quality of life in nursing home residents with dementia. Scandinavian Journal of Occupational Therapy. 2019;26(6):433-40.

216. Keating F, Cole L, Grant R. An evaluation of group reminiscence arts sessions for people with dementia living in care homes. Dementia. 2020;19(3):805-21.

217. Kennedy C, Ioannidis G, Thabane L, Adachi JD, Marr S, Giangregorio LM, et al. Successful knowledge translation intervention in long-term care: Final results from the vitamin D and osteoporosis study (ViDOS) pilot cluster randomized controlled trial. Trials. 2015;16(1).

218. Kim HJ, Lee Y, Sohng KY. The effects of footbath on sleep among the older adults in nursing home: A quasi-experimental study. Complementary Therapies in Medicine. 2016;26:40-6.

219. Kim H, Jung Y-I, Kim G-S, Choi H, Park Y-H. Effectiveness of a Technology-Enhanced Integrated Care Model for Frail Older People: A Stepped-Wedge Cluster Randomized Trial in Nursing Homes. The Gerontologist. 2021;61(3):460-9.

220. Kim H-S, Kang J-S. Effect of a group music intervention on cognitive function and mental health outcomes among nursing home residents: A randomized controlled pilot study. Geriatric Nursing. 2021;42(3):650-6.

221. Kirkham J, Maxwell C, Velkers C, Leung R, Moffat K, Seitz D. Optimizing Prescribing of Antipsychotics in Long-Term Care (OPAL): A Stepped-Wedge Trial. Journal of the American Medical Directors Association. 2020;21(3):381-7.e3.

222. Klotz AL, Hassel AJ, Schroder J, Rammelsberg P, Zenthofer A. Is compromised oral health associated with a greater risk of mortality among nursing home residents? A controlled clinical study. Aging Clinical & Experimental Research. 2018;30(6):581-8.

223. Kohler M, Schwarz J, Burgstaller M, Saxer S. Incontinence in nursing home residents with dementia : Influence of an educational program and nursing case conferences. Zeitschrift fur Gerontologie und Geriatrie. 2018;51(1):48-53.

224. Kok JS, Nielen MMA, Scherder EJA. Quality of life in small-scaled homelike nursing homes: an 8-month controlled trial. Health & Quality of Life Outcomes. 2018;16:1-.

225. Könner F, Budnick A, Kuhnert R, Wulff I, Kalinowski S, Martus P, et al. Interventions to address deficits of pharmacological pain management in nursing home residents--A cluster-randomized trial. European Journal of Pain. 2015;19(9):1331-41.

226. Kovach CR, Putz M, Guslek B, McInnes R. Do Warmed Blankets Change Pain, Agitation, Mood or Analgesic Use Among Nursing Home Residents? Pain Management Nursing. 2019;20(6):526-31.

227. Kurt D, Alpar SE. The Effect of Music Therapy on The Sense of Loneliness of Elderly Living in Nursing Home. International Journal of Caring Sciences. 2021;14(2):930-6.

228. Kuru N, Kublay G. The effect of laughter therapy on the quality of life of nursing home residents. Journal of clinical nursing. 2017;26(21-22):3354-62.

229. Kuru Alıcı N, Zorba Bahceli P, Emiroğlu ON. The preliminary effects of laughter therapy on loneliness and death anxiety among older adults living in nursing homes: A nonrandomised pilot study. International Journal of Older People Nursing. 2018;13(4):N.PAG-N.PAG.

230. Kushiro A, Shimizu K, Takada T, Kusunoki I, Aiba N. Decreased number of days of fever detection and duration of fever with continuous intake of a fermented milk drink: a randomized, double-blind, placebo-controlled study of elderly nursing home residents. Bioscience of microbiota, food and health. 2019;38(4):151-7.

231. Kütmeç Yilmaz C. Effect of progressive muscle relaxation on adaptation to old age and quality of life among older people in a nursing home: a randomized controlled trial. Psychogeriatrics. 2021;21(4):560-70.

232. Kutschar P, Berger S, Brandauer A, Freywald N, Osterbrink J, Seidenspinner D, et al. Nursing Education Intervention Effects on Pain Intensity of Nursing Home Residents with Different Levels of Cognitive Impairment: A Cluster-Randomized Controlled Trial. Journal of Pain Research. 2020;13:633-48.

233. Lamppu PJ, Finne-Soveri H, Kautiainen H, Laakkonen M-L, Laurila JV, Pitkala KH. Effects of Staff Training on Nursing Home Residents' End-Of-Life Care: A Randomized Controlled Trial. Journal of the American Medical Directors Association. 2021;22(8):1699-705.e1.

234. Laurence B, Rogers I, Grant-Mills D, Smith D, Tefera E, Barbarinde A, et al. Reducing Dental Plaque Scores in Long-term Care Facilities Using a Checklist and Random Inspections: A Pilot Study. The Bulletin of Tokyo Dental College. 2019;60(3):177-84.

235. Lavallée JF, Gray TA, Dumville J, Cullum N. Preventing pressure ulcers in nursing homes using a care bundle: A feasibility study. Health & Social Care in the Community. 2019;27(4):e417-e27.

236. Lavigne SE, Doupe MB, Iacopino AM, Mahmud S, Elliott L. The effects of power toothbrushing on periodontal inflammation in a Canadian nursing home population: A randomized controlled trial. Int J Dent Hyg. 2017 Nov;15(4):328-34.

237. Lavigne SE, Doupe MB, Iacopino AM, Mahmud SM. The effects of power toothbrushing on C-reactive protein levels in nursing home residents: A randomized controlled trial. Canadian Journal of Dental Hygiene. 2018;52(1):20-7.

238. Lee SM, Kim S, Lim CG. The effects of milk intake and whole-body vibration exercise on bone mineral density in elderly women in nursing homes. J Phys Ther Sci. 2017 Jul;29(7):1125-8.

239. Lee WJ, Park H. Effects of auricular acupressure on sleep and pain in elderly people who have osteoarthritis and live in nursing homes: A randomized, single-blind, placebo-controlled trial. Explore. 2022.

240. Leguelinel-Blache G, Castelli C, Rolain J, Bouvet S, Chkair S, Kabani S, et al. Impact of pharmacist-led multidisciplinary medication review on the safety and medication cost of the elderly people living in a nursing home: a before-after study. Expert Review of Pharmacoeconomics and Outcomes Research. 2020;20(5):481-90.

241. Lexow M, Wernecke K, Sultzer R, Bertsche T, Schiek S. Determine the impact of a structured pharmacist-led medication review - a controlled intervention study to optimise medication safety for residents in long-term care facilities. BMC Geriatrics. 2022;22(1):307.

242. Liao CY, Nace DA, Crnich CJ, Bahrainian M, Ford JH. Effect of antibiotic time-outs on modification of antibiotic prescriptions in nursing homes. Infection control and hospital epidemiology. 2020;41(6):635-40.

243. Linander CB, Kallemose T, Joergensen LM, Andersen O, Nehlin JO, Jawad BN. The effect of circadian-adjusted LED-based lighting on sleep, daytime sleepiness and biomarkers of inflammation in a randomized controlled cross-over trial by pragmatic design in elderly care home dwellers. Archives of Gerontology & Geriatrics. 2020;91:104223.

244. Little S, Rodgers G, Fitzpatrick JM. Managing deterioration in older adults in care homes: a quality improvement project to introduce an early warning tool. British Journal of Community Nursing. 2019;24(2):58-66.

245. Liu WM, Koerner J, Lam L, Johnston N, Samara J, Chapman M, et al. Improved Quality of Death and Dying in Care Homes: A Palliative Care Stepped Wedge Randomized Control Trial in Australia. Journal of the American Geriatrics Society. 2019.

246. Livingston G, Barber J, Marston L, Stringer A, Panca M, Hunter R, et al. Clinical and cost-effectiveness of the Managing Agitation and Raising Quality of Life (MARQUE) intervention for agitation in people with dementia in care homes: a single-blind, cluster-randomised controlled trial. The Lancet Psychiatry. 2019;6(4):293-304.

247. Logan PA, Horne JC, Gladman JRF, Gordon AL, Sach T, Clark A, et al. Multifactorial falls prevention programme compared with usual care in UK care homes for older people: multicentre cluster randomised controlled trial with economic evaluation. BMJ. 2021;375:e066991.

248. Lok N, Lok S, Canbaz M. The effect of physical activity on depressive symptoms and quality of life among elderly nursing home residents: Randomized controlled trial. Archives of Gerontology & Geriatrics. 2017;70:92-8.

249. Low L-F, Russell F, McDonald T, Kauffman A. Grandfriends, an Intergenerational Program for Nursing-Home Residents and Preschoolers: A Randomized Trial. Journal of Intergenerational Relationships. 2015;13(3):227-40.

250. Low LF, Carroll S, Merom D, Baker JR, Kochan N, Moran F, et al. We think you can dance! A pilot randomised controlled trial of dance for nursing home residents with moderate to severe dementia. Complementary Therapies in Medicine. 2016;29:42-4.

251. Machacova K, Vankova H, Volicer L, Veleta P, Holmerova I. Dance as Prevention of Late Life Functional Decline Among Nursing Home Residents. Journal of Applied Gerontology. 2017;36(12):1453-70.

252. Mackey DC, Lachance CC, Wang PT, Feldman F, Laing AC, Leung PM, et al. The flooring for injury prevention (FLIP) study of compliant flooring for the prevention of fall-related injuries in long-term care: A randomized trial. PLoS Medicine. 2019;16(6).

253. Madden GR, Argraves SM, Van Ness PH, Juthani-Mehta M. Antibiotic susceptibility of urinary isolates in nursing home residents consuming cranberry capsules versus placebo. Infection Control & Hospital Epidemiology. 2015;36(3):356-7.

254. Mahlknecht A, Krisch L, Nestler N, Bauer U, Letz N, Zenz D, et al. Impact of training and structured medication review on medication appropriateness and patient-related outcomes in nursing homes: results from the interventional study InTherAKT. BMC Geriatrics. 2019;19(1):N.PAG-N.PAG.

255. Maidment ID, Damery S, Campbell N, Seare N, Fox C, Iliffe S, et al. Medication review plus person-centred care: a feasibility study of a pharmacy-health psychology dual intervention to improve care for people living with dementia. BMC Psychiatry. 2018 Oct 19;18(1):340.

256. Mak A, Delbaere K, Refshauge K, Henwood T, Goodall S, Clemson L, et al. Sunbeam Program Reduces Rate of Falls in Long-Term Care Residents With Mild to Moderate Cognitive Impairment or Dementia: Subgroup Analysis of a Cluster Randomized Controlled Trial. Journal of the American Medical Directors Association. 2022;23(5):743-9.e1.

257. Maltais M, Rolland Y, Haÿ PE, Armaingaud D, Cestac P, Rouch L, et al. The Effect of Exercise and Social Activity Interventions on Nutritional Status in Older Adults with Dementia Living in Nursing Homes: A Randomised Controlled Trial. Journal of Nutrition, Health & Aging. 2018;22(7):824-8.

258. Maltais M, Rolland Y, Hay P-E, Armaingaud D, Vellas B, de Souto Barreto P. Six-month observational follow-up on activities of daily living in people with dementia living in nursing homes after a 6-month group based on either exercise or social activities. Aging Clinical & Experimental Research. 2019;31(3):361-6.

259. Maltais M, Rolland Y, Vellas B, Hay PE, Armaingaud D, Cestac P, et al. Effect of Exercise on Behavioral Symptoms and Pain in Patients With Dementia Living in Nursing Homes. American Journal of Alzheimer's Disease and other Dementias. 2019;34(2):89-94.

260. Man REK, Gan ATL, Constantinou M, Fenwick EK, Holloway E, Finkelstein EA, et al. Effectiveness of an innovative and comprehensive eye care model for individuals in residential care facilities: results of the residential ocular care (ROC) multicentred randomised controlled trial. British Journal of Ophthalmology. 2020;104(11):1585-90.

261. Marmeleira J, Galhardas L, Raimundo A. Exercise merging physical and cognitive stimulation improves physical fitness and cognitive functioning in older nursing home residents: a pilot study. Geriatric Nursing. 2018;39(3):303-9.

262. Martin RS, Hayes BJ, Hutchinson A, Tacey M, Yates P, Lim WK. Introducing Goals of Patient Care in Residential Aged Care Facilities to Decrease Hospitalization: A Cluster Randomized Controlled Trial. Journal of the American Medical Directors Association. 2019;20(10):1318-24.e2.

263. Mbakile-Mahlanza L, van der Ploeg ES, Busija L, Camp C, Walker H, O'Connor DW. A cluster-randomized crossover trial of Montessori activities delivered by family carers to nursing home residents with behavioral and psychological symptoms of dementia. International psychogeriatrics. 2020;32(3):347-58.

264. McCabe M, Beattie E, Karantzas G, Busija L, Mellor D, von Treuer K, et al. An evaluation of a consumer directed care training program for nursing home staff. Geriatric Nursing. 2022;43:227-34.

265. McCabe MP, Beattie E, Karantzas G, Mellor D, Sanders K, Busija L, et al. Consumer directed care in residential aged care: an evaluation of a staff training program. Aging & Mental Health. 2020;24(4):673-8.

266. McConeghy KW, Davidson HE, Canaday DH, Han L, Saade E, Mor V, et al. Cluster-randomized Trial of Adjuvanted Versus Nonadjuvanted Trivalent Influenza Vaccine in 823 US Nursing Homes. Clinical Infectious Diseases. 2021;73(11):e4237-e43.

267. McCord A, Cocks B, Barreiros AR, Bizo LA. Short video game play improves executive function in the oldest old living in residential care. Computers in Human Behavior. 2020;108:N.PAG-N.PAG.

268. McCreedy EM, Sisti A, Gutman R, Dionne L, Rudolph JL, Baier R, et al. Pragmatic Trial of Personalized Music for Agitation and Antipsychotic Use in Nursing Home Residents With Dementia. Journal of the American Medical Directors Association. 2022;23(7):1171-7.

269. McDerby N, Kosari S, Bail K, Shield A, Peterson G, Naunton M. The effect of a residential care pharmacist on medication administration practices in aged care: A controlled trial. Journal of Clinical Pharmacy and Therapeutics. 2019;44(4):595-602.

270. McDermid J, Da Silva MV, Williams G, Khan Z, Corbett A, Ballard C. A Randomized Controlled Trial of a Digital Adaptation of the WHELD Person-Centered Nursing Home Training Program. Journal of the American Medical Directors Association. 2022;23(7):1166-70.

271. McGilton KS, Rochon E, Sidani S, Shaw A, Ben-David BM, Saragosa M, et al. Can We Help Care Providers Communicate More Effectively With Persons Having Dementia Living in Long-Term Care Homes? American Journal of Alzheimer's Disease & Other Dementias. 2017;32(1):41-50.

272. McMaughan DK, Nwaiwu O, Hongwei Z, Frentzel E, Mehr D, Imanpour S, et al. Impact of a decision-making aid for suspected urinary tract infections on antibiotic overuse in nursing homes. BMC Geriatrics. 2016;16:1-9.

273. Meeks S, Van Haitsma K, Schoenbachler B, Looney SW. BE-ACTIV for depression in nursing homes: primary outcomes of a randomized clinical trial. Journals of Gerontology Series B-Psychological Sciences & Social Sciences. 2015;70(1):13-23.

274. Mestres C, Hernandez M, Llagostera B, Espier M, Chandre M. Improvement of pharmacological treatments in nursing homes: Medication review by consultant pharmacists. European Journal of Hospital Pharmacy. 2015;22(4):207-11.

275. Middelstadt J, Folkerts AK, Blawath S, Kalbe E. Cognitive Stimulation for People with Dementia in Long-Term Care Facilities: Baseline Cognitive Level Predicts Cognitive Gains, Moderated by Depression. Journal of Alzheimer's Disease. 2016;54(1):253-68.

276. Millett G, Fiocco AJ. A pilot study implementing the JAVA Music Club in residential care: impact on cognition and psychosocial health. Aging & Mental Health. 2021;25(10):1848-56.

277. Mitchell SL, Shaffer ML, Cohen S, Hanson LC, Habtemariam D, Volandes AE. An Advance Care Planning Video Decision Support Tool for Nursing Home Residents With Advanced Dementia: A Cluster Randomized Clinical Trial. JAMA Internal Medicine. 2018;178(7):961-9.

278. Mitchell SL, D'Agata EMC, Hanson LC, Loizeau AJ, Habtemariam DA, Tsai T, et al. The Trial to Reduce Antimicrobial Use in Nursing Home Residents With Alzheimer Disease and Other Dementias (TRAIN-AD): A Cluster Randomized Clinical Trial. JAMA internal medicine. 2021;181(9):1174-82.

279. Mitchell SL, Volandes AE, Gutman R, Gozalo PL, Ogarek JA, Loomer L, et al. Advance Care Planning Video Intervention Among Long-Stay Nursing Home Residents: A Pragmatic Cluster Randomized Clinical Trial. JAMA internal medicine. 2020;180(8):1070-8.

280. Loomer L, Ogarek JA, Mitchell SL, Volandes AE, Gutman R, Gozalo PL, et al. Impact of an Advance Care Planning Video Intervention on Care of Short-Stay Nursing Home Patients. Journal of the American Geriatrics Society. 2021;69(3):735-43.

281. Moyo P, Loomer L, Teno JM, Gutman R, McCreedy EM, Belanger E, et al. Effect of a Video-Assisted Advance Care Planning Intervention on End-of-Life Health Care Transitions Among Long-Stay Nursing Home Residents. Journal of the American Medical Directors Association. 2022;23(3):394-8.

282. Mitolo M, Borella E, Meneghetti C, Carbone E, Pazzaglia F. How to enhance route learning and visuo-spatial working memory in aging: a training for residential care home residents. Aging & Mental Health. 2017;21(5):562-70.

283. Miyazaki A, Okuyama T, Mori H, Sato K, Ichiki M, Nouchi R. Drum Communication Program Intervention in Older Adults With Cognitive Impairment and Dementia at Nursing Home: Preliminary Evidence From Pilot Randomized Controlled Trial. Frontiers in aging neuroscience. 2020;12:142.

284. Mody L, Krein SL, Saint SK, Min LC, Montoya A, Lansing B, et al. A targeted infection prevention intervention in nursing home residents with indwelling devices: a randomized clinical trial. JAMA Internal Medicine. 2015;175(5):714-23.

285. Mody L, Gontjes KJ, Cassone M, Gibson KE, Lansing BJ, Mantey J, et al. Effectiveness of a Multicomponent Intervention to Reduce Multidrug-Resistant Organisms in Nursing Homes: A Cluster Randomized Clinical Trial. JAMA network open. 2021;4(7):e2116555.

286. Moniz-Cook E, Hart C, Woods B, Whitaker C, James I, Russell I, et al. Programme Grants for Applied Research. Challenge Demcare: management of challenging behaviour in dementia at home and in care homes – development, evaluation and implementation of an online individualised intervention for care homes; and a cohort study of specialist community mental health care for families. Southampton (UK): NIHR Journals Library; 2017.

287. Morales KA. Testing the Effect of a Resident-Focused Hand Hygiene Intervention in a Long-Term Care Facility: A Mixed Methods Feasibility Study 2017.

288. Morgan DJ, Zhan M, Goto M, Franciscus C, Alexander B, Vaughan-Sarrazin M, et al. The effectiveness of contact precautions on methicillin- resistant staphylococcus aureus in long-term care across the united states. Clinical Infectious Diseases. 2020;71(7):1676-83.

289. Mouton A, Gillet N, Mouton F, Van Kann D, Bruyere O, Cloes M, et al. Effects of a giant exercising board game intervention on ambulatory physical activity among nursing home residents: a preliminary study. Clinical Interventions in Aging. 2017;12:847-58.

290. Moyle W, Murfield J, Jones C, Beattie E, Draper B, Ownsworth T. Can lifelike baby dolls reduce symptoms of anxiety, agitation, or aggression for people with dementia in long-term care? Findings from a pilot randomised controlled trial. Aging & Mental Health. 2019;23(10):1442-50.

291. Mulasso A, Roppolo M, Liubicich ME, Settanni M, Rabaglietti E. A Multicomponent Exercise Program for Older Adults Living in Residential Care Facilities: Direct and Indirect Effects on Physical Functioning. Journal of Aging and Physical Activity. 2015;23(3):409-16.

292. Nace DA, Hanlon JT, Crnich CJ, Drinka PJ, Schweon SJ, Anderson G, et al. A Multifaceted Antimicrobial Stewardship Program for the Treatment of Uncomplicated Cystitis in Nursing Home Residents. JAMA internal medicine. 2020;180(7):944-51.

293. Naczk M, Marszalek S, Naczk A. Inertial Training Improves Strength, Balance, and Gait Speed in Elderly Nursing Home Residents. Clinical Interventions in Aging. 2020;15:177-84.

294. Nagata S, Asahara T, Wang C, Suyama Y, Chonan O, Takano K, et al. The Effectiveness of Lactobacillus Beverages in Controlling Infections among the Residents of an Aged Care Facility: A Randomized Placebo-Controlled Double-Blind Trial. Annals of Nutrition & Metabolism. 2016;68(1):51-9.

295. Nagayama H, Tomori K, Ohno K, Takahashi K, Ogahara K, Sawada T, et al. Effectiveness and Cost-Effectiveness of Occupation-Based Occupational Therapy Using the Aid for Decision Making in Occupation Choice (ADOC) for Older Residents: Pilot Cluster Randomized Controlled Trial. PLoS ONE. 2016;11(3):e0150374.

296. Nam M, Uhm D. A comparative study of the effects of intra and extra circumoral exercise for older people on oral health at nursing homes: a non-equivalent trial. Journal of Advanced Nursing. 2016;72(9):2114-23.

297. Namasivayam-MacDonald AM, Burnett L, Nagy A, Waito AA, Steele CM. Effects of Tongue Strength Training on Mealtime Function in Long-Term Care. American Journal of Speech-Language Pathology. 2017;26(4):1213-24.

298. Nawrat-Szoltysik AJ, Polak A, Malecki A, Piejko L, Grzybowska-Ganszczyk D, Krecichwost M, et al. Effect of physical activity on the sequelae of osteoporosis in female residents of residential care facilities. Advances in Clinical & Experimental Medicine. 2018;27(5):633-42.

299. Nawrat-Szoltysik A, Miodonska Z, Opara J, Polak A, Matyja B, Malecki A. Effect of Physical Activity on the Quality of Life in Osteoporotic Females Living in Residential Facilities: A Randomized Controlled Trial. Journal of geriatric physical therapy. 2019;42(2):98-104.

300. Nishiura Y, Hoshiyama M, Konagaya Y. Use of parametric speaker for older people with dementia in a residential care setting: A preliminary study of two cases. Hong Kong Journal of Occupational Therapy. 2018;31(1):30-5.

301. Obayashi K, Kodate N, Masuyama S. Measuring the impact of age, gender and dementia on communication‐robot interventions in residential care homes. Geriatrics & gerontology international. 2020;20(4):373-8.

302. Okan F, Zincir H, Deveci K. The Effect of Sun Light Exposure to the Level of Vitamin D in Elderly People Living in Nursing Home. Journal of clinical densitometry. 2022;25(2):261-71.

303. Okkels SL, Dybdal DR, Pedersen RJ, Klausen TW, Olsen A, Beck AM, et al. A culinary twist of a two-course meals-on-wheels menu in a cluster-randomized controlled trial influencing health-related quality of life in nursing home residents. Clinical Nutrition. 2021;43:137-47.

304. Olsen C, Pedersen I, Bergland A, Enders‐Slegers MJ, Patil G, Ihlebæk C. Effect of animal-assisted interventions on depression, agitation and quality of life in nursing home residents suffering from cognitive impairment or dementia: a cluster randomized controlled trial. International Journal of Geriatric Psychiatry. 2016;31(12):1312-21.

305. Onieva-Zafra MD, Hernández-Garcia L, Gonzalez-del-Valle MT, Parra-Fernández ML, Fernandez-Martinez E. Music Intervention With Reminiscence Therapy and Reality Orientation for Elderly People With Alzheimer Disease Living in a Nursing Home: A Pilot Study. Holistic Nursing Practice. 2018;32(1):43-50.

306. O'Sullivan JL, Lech S, Gellert P, Grittner U, Voigt-Antons J-N, Moller S, et al. A tablet-based intervention for activating nursing home residents with dementia: results from a cluster-randomized controlled trial. International psychogeriatrics. 2022;34(2):129-41.

307. Overgaard C, Boggild H, Hede B, Bagger M, Hartmann LG, Aagaard K. Improving oral health in nursing home residents: A cluster randomized trial of a shared oral care intervention. Community dentistry and oral epidemiology. 2022;50(2):115-23.

308. Papadopoulos C, Castro N, Nigath A, Davidson R, Faulkes N, Menicatti R, et al. The CARESSES Randomised Controlled Trial: Exploring the Health-Related Impact of Culturally Competent Artificial Intelligence Embedded Into Socially Assistive Robots and Tested in Older Adult Care Homes. International journal of social robotics. 2022;14(1):245-56.

309. Park Y, Oh S, Chang H, Bang H. Effects of the Evidence-Based Nursing Care Algorithm of Dysphagia for Nursing Home Residents. Journal of Gerontological Nursing. 2015;41(11):30-9.

310. Park M, Park H. Is Hand Massage with the Preferred Aroma Oil better than Lavender on Stress and Sleep for Long-term Care Facility Residents? Korean Journal of Adult Nursing. 2019;31(2):156-64.

311. Parsons EL, Stratton RJ, Cawood AL, Smith TR, Elia M. Oral nutritional supplements in a randomised trial are more effective than dietary advice at improving quality of life in malnourished care home residents. Clinical Nutrition. 2017;36(1):134-42.

312. Elia M, Parsons EL, Cawood AL, Smith TR, Stratton RJ. Cost-effectiveness of oral nutritional supplements in older malnourished care home residents. Clinical Nutrition. 2018;37(2):651-8.

313. Pasay DK, Guirguis MS, Shkrobot RC, Slobodan JP, Wagg AS, Sadowski CA, et al. Antimicrobial stewardship in rural nursing homes: Impact of interprofessional education and clinical decision tool implementation on urinary tract infection treatment in a cluster randomized trial. Infection control and hospital epidemiology. 2019;40(4):432-7.

314. Pasina L, Marengoni A, Ghibelli S, Suardi F, Djade C, Nobili A, et al. A Multicomponent Intervention to Optimize Psychotropic Drug Prescription in Elderly Nursing Home Residents: An Italian Multicenter, Prospective, Pilot Study. Drugs & Aging. 2016;33(2):143-9.

315. Pasquel FJ, Powell W, Peng L, Johnson TM, Sadeghi-Yarandi S, Newton C, et al. A randomized controlled trial comparing treatment with oral agents and basal insulin in elderly patients with type 2 diabetes in long-term care facilities. BMJ Open Diabetes Research and Care. 2015;3(1).

316. Passmore T, Dongwook C, Lindenmeier D, Dao B. Effects of resistance band exercise and reported life-satisfaction with older adults residing in a long-term care facility. American Journal of Recreation Therapy. 2018;17(4):19-26.

317. Pedersen LH, Gregersen M, Barat I, Damsgaard EM. Early geriatric follow-up visits to nursing home residents reduce the number of readmissions: a quasi-randomised controlled trial. European Geriatric Medicine. 2018;9(3):329-37.

318. Pepera G, Christina M, Katerina K, Argirios P, Varsamo A. Effects of multicomponent exercise training intervention on hemodynamic and physical function in older residents of long-term care facilities: A multicenter randomized clinical controlled trial. Journal of bodywork and movement therapies. 2021;28:231-7.

319. Pérez-Ros P, Cubero-Plazas L, Mejías-Serrano T, Cunha C, Martínez-Arnau FM. Preferred Music Listening Intervention in Nursing Home Residents with Cognitive Impairment: A Randomized Intervention Study. Journal of Alzheimer's Disease. 2019;70(2):431-40.

320. Peterson LR, Boehm S, Beaumont JL, Patel PA, Schora DM, Peterson KE, et al. Reduction of methicillin-resistant Staphylococcus aureus infection in long-term care is possible while maintaining patient socialization: A prospective randomized clinical trial. American Journal of Infection Control. 2016;44(12):1622-7.

321. Pinazo-Clapes C, Pinazo-Hernandis S, Sales A. Effects of an Educational Program for Professional Caregivers on Behavioral Alterations in Nursing Home Residents: Pilot Study. International journal of environmental research and public health. 2020;17(23).

322. Rantz M, Petroski GF, Popejoy LL, Vogelsmeier AA, Canada KE, Galambos C, et al. Longitudinal Impact of APRNs on Nursing Home Quality Measures in the Missouri Quality Initiative. Journal of Nutrition, Health & Aging. 2021;25(9):1124-30.

323. Ray KD, Mittelman MS. Music therapy: A nonpharmacological approach to the care of agitation and depressive symptoms for nursing home residents with dementia. Dementia. 2017;16(6):689-710.

324. Rezola-Pardo C, Arrieta H, Gil SM, Zarrazquin I, Yanguas JJ, Lopez MA, et al. Comparison between multicomponent and simultaneous dual-task exercise interventions in long-term nursing home residents: the Ageing-ONDUAL-TASK randomized controlled study. Age and Ageing. 2019;48(6):817-23.

325. Rezola-Pardo C, Rodriguez-Larrad A, Gomez-Diaz J, Lozano-Real G, Mugica-Errazquin I, Patino MJ, et al. Comparison Between Multicomponent Exercise and Walking Interventions in Long-Term Nursing Homes: A Randomized Controlled Trial. The Gerontologist. 2020;60(7):1364-73.

326. Rezola-Pardo C, Hervas G, Arrieta H, Hernandez-de Diego A, Ruiz-Litago F, Gil SM, et al. Physical exercise interventions have no effect on serum BDNF concentration in older adults living in long-term nursing homes. Experimental gerontology. 2020;139:111024.

327. Rezola-Pardo C, Irazusta J, Mugica-Errazquin I, Gamio I, Sarquis-Adamson Y, Gil SM, et al. Effects of multicomponent and dual-task exercise on falls in nursing homes: The AgeingOn Dual-Task study. Maturitas. 2022;164:15-22.

328. Richter C, Berg A, Langner H, Meyer G, Köpke S, Balzer K, et al. Effect of person-centred care on antipsychotic drug use in nursing homes (EPCentCare): a cluster-randomised controlled trial. Age and Ageing. 2019;48(3):419-25.

329. Riggs S, Blue C, Golden J. The oral health quality of life for seniors in residential facilities who have direct access to care as compared to those without access. Geriatric Nursing. 2020;41(4):406-10.

330. Roets-Merken LM, Zuidema SU, Vernooij-Dassen MJFJ, Teerenstra S, Hermsen PGJM, Kempen GIJM, et al. Effectiveness of a nurse-supported self-management programme for dual sensory impaired older adults in long-term care: A cluster randomised controlled trial. BMJ Open. 2018;8(1).

331. Rolland Y, Mathieu C, Piau C, Cayla F, Bouget C, Vellas B, et al. Improving the Quality of Care of Long-Stay Nursing Home Residents in France. Journal of the American Geriatrics Society. 2016;64(1):193-9.

332. Rolland Y, Tavassoli N, de Souto Barreto P, Perrin A, Laffon de Mazieres C, Rapp T, et al. Systematic Dementia Screening by Multidisciplinary Team Meetings in Nursing Homes for Reducing Emergency Department Transfers: The IDEM Cluster Randomized Clinical Trial. JAMA network open. 2020;3(2):e200049.

333. Romøren M, Gjelstad S, Lindbaek M. A structured training program for health workers in intravenous treatment with fluids and antibiotics in nursing homes: A modified stepped-wedge cluster-randomised trial to reduce hospital admissions. PLoS ONE. 2017;12(9):e0182619.

334. Roos C, Silen M, Skytt B, Engstrom M. An intervention targeting fundamental values among caregivers at residential facilities: effects of a cluster-randomized controlled trial on residents' self-reported empowerment, person-centered climate and life satisfaction. BMC Geriatrics. 2016;16:130.

335. Rostad HM, Utne I, Grov EK, Småstuen MC, Puts M, Halvorsrud L. The impact of a pain assessment intervention on pain score and analgesic use in older nursing home residents with severe dementia: A cluster randomised controlled trial. International Journal of Nursing Studies. 2018;84:52.

336. Roughead EE, Pratt NL, Parfitt G, Rowett D, Kalisch-Ellett LM, Bereznicki L, et al. Effect of an ongoing pharmacist service to reduce medicine-induced deterioration and adverse reactions in aged-care facilities (nursing homes): a multicentre, randomised controlled trial (the ReMInDAR trial). Age and Ageing. 2022;51(4).

337. Rutten JJS, van Buul LW, Smalbrugge M, Geerlings SE, Gerritsen DL, Natsch S, et al. An Electronic Health Record Integrated Decision Tool and Supportive Interventions to Improve Antibiotic Prescribing for Urinary Tract Infections in Nursing Homes: A Cluster Randomized Controlled Trial. Journal of the American Medical Directors Association. 2022;23(3):387-93.

338. Ryuichi O, Yoshinori R. Improvement in palliative care quality in rural nursing homes through information and communication technology-driven interprofessional collaboration. Rural & Remote Health. 2021;21(2):104-8.

339. Saal S, Klingshirn H, Beutner K, Strobl R, Grill E, Muller M, et al. Improved participation of older people with joint contractures living in nursing homes: feasibility of study procedures in a cluster-randomised pilot trial. Trials. 2019;20(1):411.

340. Sackley CM, Walker MF, Burton CR, Watkins CL, Mant J, Roalfe AK, et al. An occupational therapy intervention for residents with stroke related disabilities in UK care homes (OTCH): cluster randomised controlled trial. BMJ. 2015;350:h468-h.

341. Sackley CM, Walker MF, Burton CR, Watkins CL, Mant J, Roalfe AK, et al. An Occupational Therapy intervention for residents with stroke-related disabilities in UK Care Homes (OTCH): cluster randomised controlled trial with economic evaluation. Health Technology Assessment (Winchester, England). 2016;20(15):1-138.

342. Sado M, Funaki K, Ninomiya A, Knapp M, Mimura M. Does the Combination of the Cognitive Interventions Improve the Function of Daily Living and Save the Long-Term Care Cost? A Pilot Study of Effectiveness and Cost Saving Analysis of "Learning Therapy" for People with Dementia. Journal of Alzheimer's disease. 2020;74(3):775-84.

343. Saevareid TJL, Thoresen L, Gjerberg E, Lillemoen L, Pedersen R. Improved patient participation through advance care planning in nursing homes-A cluster randomized clinical trial. Patient education and counseling. 2019;102(12):2183-91.

344. Sahin S, Aykar FS, Yildirim Y, Jahanpeyma P. The Impact of the Otago Exercise Program on Frailty and Empowerment in Older Nursing Home Residents: A Randomized Controlled Trial. Annals of Geriatric Medicine and Research. 2022;26(1):25-32.

345. Saint-Bryant CA, Murrill J, Hayward JK, Nunez K-M, Spector A. SettleIN: Using a Manualised Intervention to Facilitate the Adjustment of Older Adults with Dementia Following Placement into Residential Care. International journal of environmental research and public health. 2020;17(7).

346. Samefors M, Tengblad A, Ostgren CJ. Sunlight Exposure and Vitamin D Levels in Older People- An Intervention Study in Swedish Nursing Homes. Journal of Nutrition, Health & Aging. 2020;24(10):1047-52.

347. Sampson EL, Barber J, Gillam J, La Frenais F, Lambe K, Laybourne A, et al. Development, feasibility, and acceptability of an intervention to improve care for agitation in people living in nursing homes with dementia nearing the end-of-life. International psychogeriatrics. 2021;33(10):1069-81.

348. Sampson EL, Feast A, Blighe A, Froggatt K, Hunter R, Marston L, et al. Pilot cluster randomised trial of an evidence-based intervention to reduce avoidable hospital admissions in nursing home residents (Better Health in Residents of Care Homes with Nursing-BHiRCH-NH Study). BMJ Open. 2020;10(12):e040732.

349. Sanchez S, Payet C, Herr M, Ecarnot F, Blochet C, Armaingaud D, et al. No impact of a prescription booklet on medication consumption in nursing home residents from 2011 to 2014: a controlled before-after study. Aging Clinical & Experimental Research. 2021;33(6):1599-607.

350. Santagata F, Massaia M, D'Amelio P. The doll therapy as a first line treatment for behavioral and psychologic symptoms of dementia in nursing homes residents: a randomized, controlled study. BMC Geriatrics. 2021;21(1):545.

351. Santamaria N, Gerdtz M, Kapp S, Wilson L, Gefen A. A randomised controlled trial of the clinical effectiveness of multi‐layer silicone foam dressings for the prevention of pressure injuries in high‐risk aged care residents: The Border III Trial. International Wound Journal. 2018;15(3):482-90.

352. Saredakis D, Hannah ADK, Corlis M, Ghezzi ES, Loffler H, Loetscher T. The Effect of Reminiscence Therapy Using Virtual Reality on Apathy in Residential Aged Care: Multisite Nonrandomized Controlled Trial. Journal of Medical Internet Research. 2021.

353. Saredakis D, Keage HAD, Corlis M, Loetscher T. Using Virtual Reality to Improve Apathy in Residential Aged Care: Mixed Methods Study. Journal of Medical Internet Research. 2020;22(6):N.PAG-N.PAG.

354. Seemer J, Volkert D, Fleckenstein-Susmann D, Bader-Mittermaier S, Sieber CC, Kiesswetter E. Usual Protein Intake Amount and Sources of Nursing Home Residents with (Risk of) Malnutrition and Effects of an Individualized Nutritional Intervention: An enable Study. Nutrients. 2021;13(7).

355. Seemer J, Kiesswetter E, Fleckenstein-Susmann D, Gloning M, Bader-Mittermaier S, Sieber CC, et al. Effects of an individualised nutritional intervention to tackle malnutrition in nursing homes: a pre-post study. European Geriatric Medicine. 2022;13(3):741-52.

356. Seleskog B, Lindqvist L, Wårdh I, Engström A, Bültzingslöwen I. Theoretical and hands‐on guidance from dental hygienists promotes good oral health in elderly people living in nursing homes, a pilot study. International Journal of Dental Hygiene. 2018;16(4):476-83.

357. Shaw C, Williams KN, Perkhounkova Y. Educating Nursing Home Staff in Dementia Sensitive Communication: Impact on Antipsychotic Medication Use. Journal of the American Medical Directors Association. 2018;19(12):1129-32.

358. Shin JH. Doll therapy: an intervention for nursing home residents with dementia. Journal of Psychosocial Nursing & Mental Health Services. 2015;53(1):13-8.

359. Sjögren K, Bergland Å, Kirkevold M, Lindkvist M, Lood Q, Sandman PO, et al. Effects of a person‐centred and thriving‐promoting intervention on nursing home residents' experiences of thriving and person‐centredness of the environment. Nursing open. 2022;9(4):2117-29.

360. Sloane PD, Zimmerman S, Ward K, Kistler CE, Paone D, Weber DJ, et al. A 2-Year Pragmatic Trial of Antibiotic Stewardship in 27 Community Nursing Homes. Journal of the American Geriatrics Society. 2020;68(1):46-54.

361. Sluggett JK, Chen EYH, Ilomaki J, Corlis M, Van Emden J, Hogan M, et al. Reducing the Burden of Complex Medication Regimens: SImplification of Medications Prescribed to Long-tErm care Residents (SIMPLER) Cluster Randomized Controlled Trial. Journal of the American Medical Directors Association. 2020;21(8):1114-20.e4.

362. Sluggett JK, Hopkins RE, Chen EY, Ilomaki J, Corlis M, Van Emden J, et al. Impact of Medication Regimen Simplification on Medication Administration Times and Health Outcomes in Residential Aged Care: 12 Month Follow Up of the SIMPLER Randomized Controlled Trial. Journal of clinical medicine. 2020;9(4).

363. Smeets CHW, Smalbrugge M, Koopmans RTCM, Nelissen-Vrancken MHJMG, van der Spek K, Teerenstra S, et al. Can the PROPER intervention reduce psychotropic drug prescription in nursing home residents with dementia? Results of a cluster-randomized controlled trial. International psychogeriatrics. 2021;33(6):577-86.

364. Sprangers S, Dijkstra K, Romijn-Luijten A. Communication skills training in a nursing home: effects of a brief intervention on residents and nursing aides. Clinical Interventions in Aging. 2015;10:311-9.

365. Stacpoole M, Hockley J, Thompsell A, Simard J, Volicer L. The Namaste Care programme can reduce behavioural symptoms in care home residents with advanced dementia. International Journal of Geriatric Psychiatry. 2015;30(7):702-9.

366. Stensvik G-T, Helvik A-S, Haugan G, Steinsbekk A, Salvesen O, Nakrem S. The short-term effect of a modified comprehensive geriatric assessment and regularly case conferencing on neuropsychiatric symptoms in nursing homes: a cluster randomized trial. BMC Geriatrics. 2022;22(1):316.

367. Stephens M, Bartley C. Use of night-time positioning equipment in care home residents with postural asymmetry: a pilot study. Nursing older people. 2020;32(3):17-24.

368. Stow R, Ives N, Smith C, Rick C, Rushton A. A cluster randomised feasibility trial evaluating nutritional interventions in the treatment of malnutrition in care home adult residents. Trials. 2015;16:433.

369. Strauven G, Anrys P, Vandael E, Henrard S, De Lepeleire J, Spinewine A, et al. Cluster-Controlled Trial of an Intervention to Improve Prescribing in Nursing Homes Study. Journal of the American Medical Directors Association. 2019.

370. Sullivan GJ. Effects of a Story-Sharing Intervention on Depression and Well-Being in Older Adults Transitioning to Long-Term Care 2017.

371. Sultana M, Campbell K, Jennings M, Montero-Odasso M, Orange JB, Knowlton J, et al. Virtual Reality Experience Intervention May Reduce Responsive Behaviors in Nursing Home Residents with Dementia: A Case Series. Journal of Alzheimer's disease. 2021;84(2):883-93.

372. Surr CA, Holloway I, Walwyn RE, Griffiths AW, Meads D, Kelley R, et al. Dementia Care Mapping TM to reduce agitation in care home residents with dementia: the EPIC cluster RCT. Health technology assessment (Winchester, England). 2020;24(16):1-172.

373. Surr CA, Holloway I, Walwyn REA, Griffiths AW, Meads D, Martin A, et al. Effectiveness of Dementia Care Mapping™ to reduce agitation in care home residents with dementia: an open-cohort cluster randomised controlled trial. Aging & Mental Health. 2021;25(8):1410-23.

374. Suzuki M, Miyazaki H, Kamei J, Yoshida M, Taniguchi T, Nishimura K, et al. Ultrasound-assisted prompted voiding care for managing urinary incontinence in nursing homes: A randomized clinical trial. Neurourology and Urodynamics. 2019;38(2):757-63.

375. Swales B, Ryde GC, Whittaker AC. A Randomized Controlled Feasibility Trial Evaluating a Resistance Training Intervention With Frail Older Adults in Residential Care: The Keeping Active in Residential Elderly Trial. Journal of Aging and Physical Activity. 2022;30(3):364-88.

376. Tadrous M, Fung K, Desveaux L, Gomes T, Taljaard M, Grimshaw JM, et al. Effect of Academic Detailing on Promoting Appropriate Prescribing of Antipsychotic Medication in Nursing Homes: A Cluster Randomized Clinical Trial. JAMA network open. 2020;3(5):e205724.

377. Tappen RM, Worch SM, Newman DO, Hain D. Evaluation of a Novel Decision Guide "Go to the Hospital or Stay Here?" for Nursing Home Residents and Families: A Randomized Trial. Research in Gerontological Nursing. 2020;13(6):309-19.

378. Taylor L, Kerse N, Klenk J, Borotkanics R, Maddison R. Exergames to Improve the Mobility of Long-Term Care Residents: A Cluster Randomized Controlled Trial. Games for Health Journal. 2018;7(1):37-42.

379. Taylor JPMB, Barker ABMP, Hill HPMBA, Haines TPPGB. Improving person-centered mobility care in nursing homes: A feasibility study. Geriatric Nursing. 2015;36(2):98.

380. Teesing GR, Richardus JH, Nieboer D, Petrignani M, Erasmus V, Verduijn-Leenman A, et al. The effect of a hand hygiene intervention on infections in residents of nursing homes: a cluster randomized controlled trial. Antimicrobial Resistance and Infection Control. 2021;10(1):80.

381. Telenius EW, Engedal K, Bergland A. Effect of a high-intensity exercise program on physical function and mental health in nursing home residents with dementia: an assessor blinded randomized controlled trial. PLoS ONE. 2015;10(5):e0126102.

382. Telenius EW, Engedal K, Bergland A. Long-term effects of a 12 weeks high-intensity functional exercise program on physical function and mental health in nursing home residents with dementia: a single blinded randomized controlled trial. BMC Geriatrics. 2015;15:158.

383. Temkin-Greener H, Ladwig S, Ye Z, Norton SA, Mukamel DB. Improving palliative care trough teamwork (IMPACTT) in nursing homes: Study design and baseline findings. Contemporary Clinical Trials. 2017;56:1-8.

384. Temkin-Greener H, Mukamel DB, Ladd H, Ladwig S, Caprio TV, Norton SA, et al. Impact of Nursing Home Palliative Care Teams on End-of-Life Outcomes: A Randomized Controlled Trial. Medical Care. 2018;56(1):11-8.

385. Ter Wee P, Kuhn M, van der Woude H, van de Looverbosch D, Heyman H, Mikušová L, et al. Gastro-intestinal tolerance and renal safety of protein oral nutritional supplements in nursing home residents: A randomized controlled trial. Journal of Nutrition, Health & Aging. 2016;20(9):944-51.

386. Testad I, Mekki TE, Førland O, Øye C, Tveit EM, Jacobsen F, et al. Modeling and evaluating evidence-based continuing education program in nursing home dementia care (MEDCED)--training of care home staff to reduce use of restraint in care home residents with dementia. A cluster randomized controlled trial. Int J Geriatr Psychiatry. 2016 Jan;31(1):24-32.

387. Thodberg K, Sorensen LU, Christensen JW, Poulsen PH, Houbak B, Damgaard V, et al. Therapeutic effects of dog visits in nursing homes for the elderly. Psychogeriatrics. 2016;16(5):289-97.

388. Thodberg K, Sørensen LU, Videbech PB, Poulsen PH, Houbak B, Damgaard V, et al. Behavioral Responses of Nursing Home Residents to Visits From a Person with a Dog,a Robot Seal or aToy Cat. Anthrozoos. 2016;29(1):107-21.

389. Travers C. Increasing enjoyable activities to treat depression in nursing home residents with dementia: A pilot study. Dementia. 2017 Feb;16(2):204-18.

390. Treusch Y, Majic T, Page J, Gutzmann H, Heinz A, Rapp MA. Apathy in nursing home residents with dementia: results from a cluster-randomized controlled trial. European Psychiatry: the Journal of the Association of European Psychiatrists. 2015;30(2):251-7.

391. Tropea J, Nestel D, Johnson C, Hayes BJ, Hutchinson AF, Brand C, et al. Evaluation of IMproving Palliative care Education and Training Using Simulation in Dementia (IMPETUS-D) a staff simulation training intervention to improve palliative care of people with advanced dementia living in nursing homes: a cluster randomised controlled trial. BMC Geriatrics. 2022;22(1):127.

392. Tsai P-F, Kitch S, Beck C, Jakobs T, Rettiganti M, Jordan K, et al. Using an Interactive Video Simulator to Improve Certified Nursing Assistants' Dressing Assistance and Nursing Home Residents' Dressing Performance: A Pilot Study. CIN: Computers, Informatics, Nursing. 2018;36(4):183-92.

393. Tsugawa A, Shimizu S, Hirose D, Sato T, Hatanaka H, Takenoshita N, et al. Effects of 12‐month exercise intervention on physical and cognitive functions of nursing home residents requiring long‐term care: a non‐randomised pilot study. Psychogeriatrics. 2020;20(4):419-26.

394. Tylner S, Cederholm T, Faxen-Irving G. Effects on Weight, Blood Lipids, Serum Fatty Acid Profile and Coagulation by an Energy-Dense Formula to Older Care Residents: A Randomized Controlled Crossover Trial. Journal of the American Medical Directors Association. 2016;17(3):275.e5-11.

395. Valiani V, Lauzé M, Martel D, Pahor M, Manini T, Anton S, et al. A new adaptive home-based exercise technology among older adults living in nursing home: A pilot study on feasibility, acceptability and physical performance. Journal of Nutrition, Health & Aging. 2017;21(7):819-24.

396. Van Bogaert P, Tolson D, Eerlingen R, Carvers D, Wouters K, Paque K, et al. SolCos model-based individual reminiscence for older adults with mild to moderate dementia in nursing homes: a randomized controlled intervention study. Journal of Psychiatric & Mental Health Nursing. 2016;23(9-10):568-75.

397. van Dam PH, Achterberg WP, Husebø BS, Caljouw MAA. Does paracetamol improve quality of life, discomfort, pain and neuropsychiatric symptoms in persons with advanced dementia living in long-term care facilities? A randomised double-blind placebo-controlled crossover (Q-PID) trial. BMC Medicine. 2020;18(1):407.

398. Van den Block L, Honinx E, Pivodic L, Miranda R, Onwuteaka-Philipsen BD, van Hout H, et al. Evaluation of a Palliative Care Program for Nursing Homes in 7 Countries: The PACE Cluster-Randomized Clinical Trial. JAMA internal medicine. 2019:1-10.

399. Miranda R, Smets T, Van Den Noortgate N, van der Steen JT, Deliens L, Payne S, et al. No difference in effects of 'PACE steps to success' palliative care program for nursing home residents with and without dementia: a pre-planned subgroup analysis of the seven-country PACE trial. BMC Palliative Care. 2021;20(1):39.

400. Van Haitsma KS, Curyto K, Abbott KM, Towsley GL, Spector A, Kleban M. A randomized controlled trial for an individualized positive psychosocial intervention for the affective and behavioral symptoms of dementia in nursing home residents. Journals of Gerontology Series B-Psychological Sciences & Social Sciences. 2015;70(1):35-45.

401. van Leen M, Halfens R, Schols J. Preventive Effect of a Microclimate-Regulating System on Pressure Ulcer Development: A Prospective, Randomized Controlled Trial in Dutch Nursing Homes. Advances in Skin & Wound Care. 2018;31(1):1-5.

402. van Welie S, Wijma L, Beerden T, van Doormaal J, Taxis K. Effect of warning symbols in combination with education on the frequency of erroneously crushing medication in nursing homes: an uncontrolled before and after study. BMJ Open. 2016;6(8):e012286.

403. van Wietmarschen HA, Busch M, van Oostveen A, Pot G, Jong MC. Probiotics use for antibiotic-associated diarrhea: a pragmatic participatory evaluation in nursing homes. BMC gastroenterology. 2020;20(1):151.

404. Van Wymelbeke V, Brondel L, Bon F, Martin-Pfitzenmeyer I, Manckoundia P. An innovative brioche enriched in protein and energy improves the nutritional status of malnourished nursing home residents compared to oral nutritional supplement and usual breakfast: FARINE+ project. Clinical Nutrition. 2016;15:93-100.

405. Van Wymelbeke V, Sulmont-Rossé C, Feyen V, Issanchou S, Manckoundia P, Maître I. Optimizing sensory quality and variety: An effective strategy for increasing meal enjoyment and food intake in older nursing home residents. Appetite. 2020;153:N.PAG-N.PAG.

406. Veleva BI, Caljouw MAA, van der Steen JT, Mertens BJA, Chel VGM, Numans ME. The Effect of Ultraviolet B Irradiation Compared with Oral Vitamin D Supplementation on the Well-being of Nursing Home Residents with Dementia: A Randomized Controlled Trial. International journal of environmental research and public health. 2020;17(5).

407. Verreault R, Arcand M, Misson L, Durand PJ, Kroger E, Aubin M, et al. Quasi-experimental evaluation of a multifaceted intervention to improve quality of end-of-life care and quality of dying for patients with advanced dementia in long-term care institutions. Palliative Medicine. 2018;32(3):613-21.

408. Viscogliosi G, Chiriac IM, Ettorre E. Efficacy and Safety of Citalopram Compared to Atypical Antipsychotics on Agitation in Nursing Home Residents With Alzheimer Dementia. Journal of the American Medical Directors Association. 2017;18(9):799-802.

409. Visscher A, Battjes-Fries MCE, van de Rest O, Patijn ON, van der Lee M, Wijma-Idsinga N, et al. Fingerfoods: a feasibility study to enhance fruit and vegetable consumption in Dutch patients with dementia in a nursing home. BMC Geriatrics. 2020;20(1):N.PAG-N.PAG.

410. Visser AGR, Schols JMGA, Prevoo MALM, Janknegt R, Winkens B. Deprescribing Statins and Proton Pump Inhibitors in Nursing Home Residents; a Pragmatic Exploratory Study. Gerontology and Geriatric Medicine. 2021;7.

411. Walker GM, Armstrong S, Gordon AL, Gladman J, Robertson K, Ward M, et al. The Falls In Care Home study: a feasibility randomized controlled trial of the use of a risk assessment and decision support tool to prevent falls in care homes. Clinical Rehabilitation. 2016;30(10):972-83.

412. Walker P, Kifley A, Kurrle S, Cameron ID. Increasing the uptake of vitamin D supplement use in Australian residential aged care facilities: results from the vitamin D implementation (ViDAus) study. BMC Geriatrics. 2020;20(1):383.

413. Wallace JP, Mohammadi J, Wallace LG, Taylor JA. Senior Smiles: preliminary results for a new model of oral health care utilizing the dental hygienist in residential aged care facilities. International Journal of Dental Hygiene. 2016;14(4):284-8.

414. Wang B, Hylwka T, Smieja M, Surrette M, Bowdish DME, Loeb M. Probiotics to Prevent Respiratory Infections in Nursing Homes: A Pilot Randomized Controlled Trial. Journal of the American Geriatrics Society. 2018;66(7):1346-52.

415. Watson K, Hatcher D, Good A. A randomised controlled trial of Lavender (Lavandula Angustifolia) and Lemon Balm (Melissa Officinalis) essential oils for the treatment of agitated behaviour in older people with and without dementia. Complementary Therapies in Medicine. 2019 Feb;42:366-73.

416. Wauters M, Elseviers M, Vander Stichele R, Dilles T, Thienpont G, Christiaens T. Efficacy, feasibility and acceptability of the OptiMEDs tool for multidisciplinary medication review in nursing homes. Archives of Gerontology & Geriatrics. 2021;95:104391.

417. Weatherall CD, Hansen AT, Nicholson S. The effect of assigning dedicated general practitioners to nursing homes. Health Services Research. 2019;54(3):547-54.

418. Weintraub JA, Zimmerman S, Ward K, Wretman CJ, Sloane PD, Stearns SC, et al. Improving Nursing Home Residents' Oral Hygiene: Results of a Cluster Randomized Intervention Trial. Journal of the American Medical Directors Association. 2018;19(12):1086-91.

419. Werner J, Wosch T, Gold C. Effectiveness of group music therapy versus recreational group singing for depressive symptoms of elderly nursing home residents: pragmatic trial. Aging & Mental Health. 2017;21(2):147-55.

420. Wesenberg S, Mueller C, Nestmann F, Holthoff‐Detto V. Effects of an animal‐assisted intervention on social behaviour, emotions, and behavioural and psychological symptoms in nursing home residents with dementia. Psychogeriatrics. 2019;19(3):219-27.

421. Westerhof GJ, Korte J, Eshuis S, Bohlmeijer ET. Precious memories: a randomized controlled trial on the effects of an autobiographical memory intervention delivered by trained volunteers in residential care homes. Aging & Mental Health. 2018;22(11):1494-501.

422. Whitney J, Jackson SHD, Martin FC. Feasibility and efficacy of a multi-factorial intervention to prevent falls in older adults with cognitive impairment living in residential care (ProF-Cog). A feasibility and pilot cluster randomised controlled trial. BMC Geriatrics. 2017;17(1):115.

423. Wijnen H, Salemink D, Roovers L, Taekema D, de Boer H. Vitamin D supplementation in nursing home patients: randomized controlled trial of standard daily dose versus individualized loading dose regimen. Drugs & Aging. 2015;32(5):371-8.

424. Wilchesky M, Mueller G, Marcotte M, Voyer P, Carmichael P-H, Champoux N, et al. The OptimaMed intervention to reduce inappropriate medications in nursing home residents with severe dementia: results from a quasi-experimental feasibility pilot study. BMC Geriatrics. 2018;18(1):1-10.

425. Williams TM. The effect of video gaming on physical activity among nursing home residents 2016.

426. Wilson J, Bak A, Tingle A, Greene C, Tsiami A, Canning D, et al. Improving hydration of care home residents by increasing choice and opportunity to drink: A quality improvement study. Clinical Nutrition. 2019;38(4):1820-7.

427. Wogamon CL. Exploring the Effect of Educating Certified Nursing Assistants on Pressure Ulcer Knowledge and Incidence in a Nursing Home Setting. Ostomy Wound Management. 2016;62(9):42-50.

428. Woloszyn N, Wisniowska-Szurlej A, Grzegorczyk J, Kwolek A. The impact of physical exercises with elements of dance movement therapy on the upper limb grip strength and functional performance of elderly wheelchair users living in nursing homes - a randomized control trial. BMC Geriatrics. 2021;21(1):423.

429. Wren R. Effect of Life Review on Quality of Life For Older Adults Living in Nursing Homes. Physical & Occupational Therapy in Geriatrics. 2016;34(4):186-204.

430. Wylie G, Menz HB, McFarlane S, Ogston S, Sullivan F, Williams B, et al. Podiatry intervention versus usual care to prevent falls in care homes: pilot randomised controlled trial (the PIRFECT study). BMC Geriatrics. 2017 Jul 12;17(1):143.

431. Yamamoto Y, Saruta J, Takahashi T, To M, Shimizu T, Hayashi T, et al. Effect of ingesting yogurt fermented with Lactobacillus delbrueckii ssp. bulgaricus OLL1073R-1 on influenza virus-bound salivary IgA in elderly residents of nursing homes: a randomized controlled trial. Acta Odontologica Scandinavica. 2019;77(7):517-24.

432. Yap TL, Horn SD, Sharkey PD, Zheng T, Bergstrom N, Colon-Emeric C, et al. Effect of Varying Repositioning Frequency on Pressure Injury Prevention in Nursing Home Residents: TEAM-UP Trial Results. Advances in Skin & Wound Care. 2022;35(6):315-25.

433. Yasuda M, Sakakibara H. Care staff training based on person-centered care and dementia care mapping, and its effects on the quality of life of nursing home residents with dementia. Aging & Mental Health. 2017;21(9):991-6.

434. Yesilyaprak SS, Yildirim MS, Tomruk M, Ertekin O, Algun ZC. Comparison of the effects of virtual reality-based balance exercises and conventional exercises on balance and fall risk in older adults living in nursing homes in Turkey. Physiotherapy Theory & Practice. 2016;32(3):191-201.

435. Yucel SC, Arslan GG, Bagci H. Effects of Hand Massage and Therapeutic Touch on Comfort and Anxiety Living in a Nursing Home in Turkey: A Randomized Controlled Trial. Journal of Religion & Health. 2019;13.

436. Zimmerman S, Sloane PD, Ward K, Wretman CJ, Stearns SC, Poole P, et al. Effectiveness of a Mouth Care Program Provided by Nursing Home Staff vs Standard Care on Reducing Pneumonia Incidence: A Cluster Randomized Trial. JAMA network open. 2020;3(6):e204321.

**Appendix 3: Supplementary descriptive tables for included studies**

**Supplementary Table 1: Summary of distribution of year of publication based on included studies**

| **Year** | **Number of studies (%)** |
| --- | --- |
| 2015 | 43 (9.9) |
| 2016 | 55 (12.6) |
| 2017 | 48 (11.0) |
| 2018 | 61 (14.0) |
| 2019 | 64 (14.7) |
| 2020 | 66 (15.1) |
| 2021 | 66 (15.1) |
| 2022 (Year to August) | 33 (7.6) |
| **Total** | **436 studies** |

**Supplementary Table 2: Summary of country of origin based on total number of included datasets**

| **Country** | **Number of datasets (%)** | **Country** | **Number of datasets (%)** |
| --- | --- | --- | --- |
| Australia | 49 (12.4) | Mexico | 1 (0.3) |
| Austria | 3 (0.8) | Multiple countries | 4 (1.0) |
| Belgium | 10 (2.5) | New Zealand | 3 (0.8) |
| Canada | 26 (6.6) | Norway | 17 (4.3) |
| Czech Republic | 1 (0.3) | Poland | 4 (1.0) |
| Denmark | 8 (2.0) | Portugal | 1 (0.3) |
| Finland | 1 (0.3) | South Korea | 11 (2.8) |
| France | 16 (4.0) | Spain | 9 (2.3) |
| Germany | 30 (7.6) | Sweden | 12 (3.0) |
| Greece | 1 (0.3) | Switzerland | 5 (1.3) |
| Hungary | 1 (0.3) | The Netherlands | 22 (5.6) |
| Ireland | 1 (0.3) | Turkey | 23 (5.8) |
| Italy | 15 (3.8) | United Kingdom | 43 (10.9) |
| Japan | 13 (3.3) | United States of America | 66 (16.7) |
| **Total** | | **396 datasets** | |

**Supplementary Table 3: Summary of study designs based on included datasets**

| Study design | N (%) |
| --- | --- |
| **Before and after studies**  Before and after (one arm)  Before and after (two arms) | 36  30 |
| **Non-randomised controlled trials**  Non-randomised controlled trial  Non-randomised controlled trial (cluster design) | 20  2 |
| **Other designs**  2x2 full factorial within-subject design  Action research study  Cluster-randomised crossover design  Evaluating effectiveness of intervention using generalised linear mixed models  Experimental crossover design of complex intervention  Longitudinal evaluation of policy intervention  Mixed methods evaluation  Participatory evaluation  Quality improvement study  Quasi-experimental repeated-measures crossover design  Quasi-experimental study design with non-equivalent control group  Quasi-experimental study using generalised linear modelling  Randomised crossover trial  Randomised trial, 3 interventions, no control  Research evaluation of practice development and sustainability initiatives  Single subject design with multiple baselines across individuals  Within-subject design with two conditions | 1  1  3  1  1  2  1  1  7  1  1  1  1  1  1  3  1 |
| **Pilot/feasibility studies**  Pilot/feasibility study (non-trial design)  Pilot/feasibility trial | 47  40 |
| **Randomised controlled trials**  Cluster randomised controlled trial  Randomised controlled trial  Randomised controlled trial post-hoc analysis  Stepped-wedge randomised controlled trial | 85  94  6  8 |

**Supplementary Table 4: Summary distribution of study sample size – homes and residents**

| **Number of residents** | **Number of care homes** | | | | | **Total** |
| --- | --- | --- | --- | --- | --- | --- |
|  | **1 home** | **2-5 homes** | **6-50 homes** | **>51 homes** | **Not reported** |  |
| **1-50** | 73 | 49 | 13 | 0 | 3 | 138 |
| **51-100** | 17 | 22 | 33 | 1 | 2 | 75 |
| **101-500** | 7 | 19 | 74 | 6 | 0 | 106 |
| **501-5000** | 0 | 1 | 23 | 14 | 0 | 38 |
| **>5001** | 0 | 0 | 3 | 10 | 1 | 14 |
| **Not reported** | 3 | 3 | 13 | 6 | 0 | 25 |
| **Total** | 100 | 94 | 159 | 37 | 6 | 396 |

**Appendix 4**

**Supplementary Table 5: Characteristics of included studies**

|  | **Study ID**  **(Author, Year(s))** | **Study design** | **Number of homes** | **Number of residents** | **Intervention target** | **Intervention type** |
| --- | --- | --- | --- | --- | --- | --- |
| 1 | Abraham 2019 | Cluster RCT | 120 | 12,245 | Physical restraint | Multicomponent (training, education, clinical guidance & resources for practice) |
| 2 | Acaroz Candan 2019 | RCT | 1 | 53 | Physical function | Technology |
| 3 | Ailabouni 2019 | Pilot/feasibility study | 3 | 46 | Prescribing - anticholinergic & sedatives | Deprescribing guidance/protocol |
| 4 | Almeida 2022 | Cluster RCT | 54 | 188 | Depression | Behaviour training |
| 5 | Alp 2021 | Non-randomised CT | 1 | 60 | Comfort & anxiety | Therapeutic touch |
| 6 | Álvarez Barbosa 2018 | Pilot/feasibility study | 2 | 44 | Physical function & quality of life | Exercise using technology |
| 7 | Anderson 2018 | Pilot/feasibility trial | NR | 18 | Mood & behaviour | Psychological therapy |
| 8 | Arendts 2018 | Non-randomised CT | 6 | 200 | Hospital transfer & quality of life | Dedicated professional support |
| 9 | Arnold 2021 | Cluster RCT | 22 | 1,625 | Prescribing - antimicrobials | Education/training |
| 10 | Arrieta 2018, 2019a, 2019b, 2020, 2022 | RCT | 10 | 112 | Physical function & frailty | Multicomponent (exercise & physical activity) |
| 11 | Ashcraft 2017 | Non-randomised CT | 2 | 56 | Hospital transfer/length of stay | Communication tool |
| 12 | Baandrup 2021 | RCT | 1 | 24 | Activity | Environmental modification |
| 13 | Bae 2020 | RCT | 2 | 58 | Wellbeing | Aromatherapy |
| 14 | Bagci 2020 | RCT | 1 | 25 | Sleep | Therapeutic touch |
| 15 | Bailey 2017 | RCT | 5 | 51 | Depression | Multicomponent (psychological therapy, environmental change & activity programme) |
| 16 | Baker 2017 | Before and after (one arm) | 2 | 24 | Mood | Intergenerational |
| 17 | Ballard 2016, Rajkumar 2016 | Pilot/feasibility trial | 16 | 277 | Agitation, depression & antipsychotic medication use | Multicomponent (training, medication review, exercise & social interaction) |
| 18 | Ballard 2018 | Cluster RCT | 69 | 837 | Quality of life, agitation & antipsychotic medication use | Multicomponent (training, education, medication review) |
| 19 | Balsom 2020 | RCT | 1 | 45 | Deprescribing | Medication review |
| 20 | Barbe 2019 | RCT | 1 | 50 | Oral health | Dedicated professional support |
| 21 | Barbe 2020 | Before and after (one arm) | 1 | 40 | Oral health | Dedicated professional support |
| 22 | Baron 2015 | Before and after | 20 | NR | Palliative/end of life care | Education/training |
| 23 | Barrett 2019 | Pilot/feasibility study | 1 | 10 | Quality of life & depression | Technology |
| 24 | Barthalos 2016 | Before and after | 1 | 45 | Quality of life, attitude to aging, assertiveness, physical fitness & body composition | Exercise |
| 25 | Bassi 2018 | Pilot/feasibility study | 1 | 11 | Quality of experience | Horticulture |
| 26 | Batchelor-Murphy 2015 | Pilot/feasibility study | 2 | 7 | Nutrition | Education/training using technology |
| 27 | Bauer 2015 | Before and after (one arm) | 2 | 16 | Behavioural & Psychological Symptoms of Dementia | Snozelen |
| 28 | Bautrant 2019 | Before and after (one arm) | 1 | 19 | Behavioural & Psychological Symptoms of Dementia | Environmental modification |
| 29 | Beaupre 2019 & 2020 | Pilot/feasibility trial | NR | 77 | Physical function | Rehabilitation |
| 30 | Beeckman 2019 & Anrys 2019 | RCT | 26 | 308 | Skincare/integrity | Equipment/devices |
| 31 | Bellini 2015 | Cluster RCT | 104 | 4,750 | Infection | Screening |
| 32 | Benigni 2018 | Pilot/feasibility trial | 1 | 38 | Skincare/integrity | Technology |
| 33 | Berkheimer 2017 | Pilot/feasibility study | 1 | 8 | Mood & behaviour | Multicomponent (Snoezelen & Exercise) |
| 34 | Bertoncello 2021 | Before and after (one arm) | 1 | 221 | Mobility & function | Exercise |
| 35 | Bischoff 2021 | Pilot/feasibility trial | 1 | 24 | Physical function & psychosocial wellbeing | Multicomponent (cognitive tasks & physical exercise) |
| 36 | Blaak 2015 | Before and after | 1 | 20 | Skincare/integrity | Pharmacological |
| 37 | Blair 2016 | Pilot/feasibility trial | 1 | 6 | Anxiety & depression | Psychological therapy |
| 38 | Blytt 2017 | RCT | 47 | 106 | Sleep | Pharmacological |
| 39 | Boere 2021 | Cluster RCT | 11 | 241 | Prescribing - antimicrobials | Technology |
| 40 | Bökberg 2019 | Other: Experimental crossover design of complex intervention | 20 | 52 | Quality of life | Education/training |
| 41 | Boockvar 2016 | Pilot/feasibility study | 1 | 143 | Delirium | Multicomponent (education, training, support including reminiscence, hydration, exercise & massage) |
| 42 | Boockvar 2020 | Cluster RCT | 1 | 219 | Delirium | Multicomponent (education & risk reduction) |
| 43 | Boogaard 2018 | Cluster RCT | 18 | 668 | Palliative/end of life care | Feedback |
| 44 | Booth 2021 | RCT | 37 | 408 | Incontinence | Technology |
| 45 | Boström 2016, Sondell 2018, 2019a, 2019b, Toots 2019 | Cluster RCT | 16 | 186 | Depression | Exercise |
| 46 | Bourdon 2021 | Non-randomised CT (cluster) | 4 | 120 | Function | Environmental modification |
| 47 | Brännström 2016 | Before and after | 19 | 260 | Palliative/end of life care | Care bundle/pathway |
| 48 | Bravo-Jose 2019 | Before and after (one arm) | 1 | 35 | Prescribing - antipsychotics | Deprescribing guidance/protocol |
| 49 | Brazil 2018 | Cluster RCT | 24 | 657 | Palliative/end of life care | Multicomponent (education, communication & visiting professional support) |
| 50 | Brett 2017, 2019 & 2021 | RCT | 2 | 60 | Physical function & falls | Exercise |
| 51 | Brimelow 2022 | Pilot/feasibility study | 1 | 25 | Mood & behaviour | Technology |
| 52 | Brodaty 2018 | Before and after (one arm) | 23 | 139 | Prescribing - antipsychotics | Deprescribing guidance/protocol |
| 53 | Brooker 2016 | Other: Mixed methods evaluation | 106 | NR | Prescribing - antipsychotics | Multicomponent (training, education & professional support) |
| 54 | Brown 2019 | Before and after (one arm) | 10 | NR | Prescribing - antimicrobials | Multicomponent (champions, coaching, education, reviewing protocols, improving documentation & signposting to resources) |
| 55 | Brustio 2015 | Pilot/feasibility trial | 1 | 35 | Mobility & function | Exercise |
| 56 | Bryant 2020 | Pilot/feasibility study | 2 | 18 | Depression | Behavioural activation |
| 57 | Butler 2020 & Castro-Herrera 2021 | RCT | 23 | 310 | Prescribing - antimicrobials | Pharmacological |
| 58 | Callegari 2022 | Cluster RCT | 14 | 217 | Medication management | Education/training |
| 59 | Cantarella 2018 | Non-randomised CT | 1 | 29 | Behavioural & Psychological Symptoms of Dementia | Doll therapy |
| 60 | Cateau 2021a | RCT | 7 | 58 | Medication management | Education/training |
| 61 | Cateau 2021b | Cluster RCT | 55 | NR | Deprescribing | Multicomponent (education & deprescribing protocols) |
| 62 | Catiker 2021 | RCT | 1 | 57 | Foot health | Education/training |
| 63 | Cavallini 2015 | RCT | 1 | 34 | Cognition | Education/training |
| 64 | Cavallini 2021 | RCT | 5 | 31 | Cognition | Education/training |
| 65 | Çetinkaya 2019 | RCT | 1 | 30 | Cognition & satisfaction with life | Group ceramic painting |
| 66 | Chalhoub 2016 | Pilot/feasibility study | 1 | 18 | Oral health | Pharmacological |
| 67 | Chambers 2022 | Non-randomised CT | 170 | NR | Prescribing - antimicrobials | Education/training using technology |
| 68 | Chiesi 2021 | Pilot/feasibility study | 2 | 16 | Agitation, depression & behaviour | Dance therapy |
| 69 | Chou 2016 | Pilot/feasibility study | 1 | 4 | Resistance to care | Images to elicit positive emotion |
| 70 | Chu 2016 & 2020 | Pilot/feasibility study | 2 | 26 | Functional mobility, activities of daily living & quality of life | Multicomponent (exercise & communication planning) |
| 71 | Churcher Clarke 2017 | Pilot/feasibility trial | 4 | 31 | Intervention feasibility | Mindfulness intervention |
| 72 | Cichocki 2015 | RCT | 3 | 222 | Physical activity | Exercise |
| 73 | Clarke-O'Neill 2015 | Pilot/feasibility study | 10 | 78 | Skincare/integrity | Equipment/devices |
| 74 | Cohen 2019 | Cluster RCT | 64 | 328 | Palliative/end of life care | Education/training using technology |
| 75 | Colón-Emeric 2017 | Cluster RCT | 24 | NR | Falls | Quality improvement |
| 76 | Connolly 2015 & 2016 | Cluster RCT | 36 | 1,998 | Hospital transfer/length of stay | Multicomponent (education, review & multidisciplinary discussions) |
| 77 | Conway 2015 | Before and after | 12 | NR | Hospital transfer/length of stay | Dedicated professional support |
| 78 | Cool 2018 | Pilot/feasibility study | 159 | 629 | Medication management | Education/training |
| 79 | Cordato 2018 | RCT | 21 | 43 | Hospital transfer/length of stay | Dedicated professional support |
| 80 | Cordes 2021 | RCT | 4 | 38 | Cognition & motor function | Exercise |
| 81 | Couderc 2021 | Before and after (one arm) | 38 | 48 | Access to care, toxicity of treatment & quality of life | Care bundle/pathway |
| 82 | Courel-Ibanez 2022 | Cluster RCT | 2 | 22 | Function & strength | Exercise |
| 83 | Crespy 2016 | Other: Quality improvement study | 40 | NR | Depression | Multicomponent (training, clinical tools & technical support) |
| 84 | Croonquist 2020 | Cluster RCT | 9 | 146 | Oral health | Dedicated professional support |
| 85 | Daneman 2021 | RCT | NR | 36,194 | Prescribing - antimicrobials | Audit & feedback |
| 86 | Davidson 2019 | Before and after | 47 | 53,126 | Skincare/integrity | Technology |
| 87 | Davison 2017 | Pilot/feasibility trial | 5 | 41 | Anxiety & depression | Psychological therapy |
| 88 | Davison 2021 | Cluster RCT | 42 | 219 | Depression | Psychological therapy |
| 89 | de Bruin 2020 | RCT | 1 | 17 | Cognition & physical function | Exercise using technology |
| 90 | De Luca 2016a | RCT | 1 | 20 | Cognition | Cognitive training |
| 91 | De Luca 2016b | RCT | 2 | 59 | Behavioural symptoms & quality of life | Telehealth |
| 92 | de Pooter-Stijnman 2018 | Pilot/feasibility study | 1 | 21 | Behavioural & Psychological Symptoms of Dementia | Caffeine reduction |
| 93 | de Souto Barreto 2017 | Pilot/feasibility trial | 7 | 97 | Cognition, physical & activities of daily living | Exercise |
| 94 | del Campo Cervantes 2019 | Before and after (one arm) | 1 | 19 | Sarcopenia & function | Exercise |
| 95 | Dellinger 2020 | Non-randomised CT | 6 | 240 | Medication management | Technology |
| 96 | Demir 2022 | RCT | NR | 40 | Balance & sensory function | Multicomponent (exercise & education) |
| 97 | Desborough 2020 | Cluster RCT | 30 | 826 | Falls | Medication review (multi-professional) |
| 98 | Devereaux 2016 | Pilot/feasibility study | 1 | NR | Hospital transfer/length of stay | Education and communication tool |
| 99 | Dharmarajan 2016 | RCT | 14 | 64 | Diabetic control | Pharmacological |
| 100 | Diegelmann 2018 | Other: Evaluating effectiveness of intervention using generalised linear mixed models | 2 | 163 | Depression | Exercise |
| 101 | Dimori 2018 | Before and after | 1 | 39 | Physical function | Multicomponent (exercise & nutritional supplement) |
| 102 | Dolu 2019 | Non-randomised CT | 3 | 52 | Mood & sleep | Multicomponent (assessment, recommendations & clinical guidance) |
| 103 | Dowson 2019 | Pilot/feasibility study | 3 | NR | Prescribing - antimicrobials | Testing |
| 104 | Dräger 2017 | Cluster RCT | 12 | 747 | Pain | Multicomponent (training & guidance tool) |
| 105 | Drotningsvik 2019 | Pilot/feasibility study | 6 | 21 | Nutrition | Nutritional supplements/dietary modification |
| 106 | Dugre 2021 | Cluster RCT | 8 | 162 | Deprescribing | Education/training |
| 107 | Duru Aşiret 2018 | RCT | 1 | 46 | Sleep | Reminiscence |
| 108 | Düzgün 2017 | Non-randomised CT | 1 | 61 | Sleep | Environmental modification |
| 109 | El Alili 2020 | Cluster RCT | 19 | 231 | Cost-effectiveness | Namaste Care |
| 110 | El Haddad 2020 | Non-randomised CT | 175 | 3,709 | Medication management | Audit & feedback |
| 111 | Ellis 2019 | Before and after (one arm) | 5 | 95 | Pain | Dedicated professional support |
| 112 | Ercan-Sahin 2018 | Before and after (one arm) | 1 | 32 | Quality of life | Reminiscence |
| 113 | Erdal 2018 | RCT | 47 | 89 | Depression | Pharmacological |
| 114 | Ergin 2019 | Before and after | 1 | 56 | Psycho-spirituality | Music therapy |
| 115 | Eriksson 2016 | Other: Single subject design with multiple baselines across individuals | 5 | 5 | Communication | Education/training using technology |
| 116 | Ersek 2016 | Cluster RCT | 27 | 485 | Pain | Multicomponent (training & guidance tool) |
| 117 | Evrard 2020 | RCT post-hoc analysis | 54 | 797 | Prescribing - benzodiazepines | Multicomponent (education, interdisciplinary meetings and case discussion) |
| 118 | Farlow 2016 | Other: Quality improvement study | 4 | 67 | Dementia | Education/training |
| 119 | Fjeld 2018 | RCT post-hoc analysis | 9 | 100 | Oral health | Technology |
| 120 | Folkerts 2018 | Pilot/feasibility study | 1 | 12 | Cognition | Cognitive stimulation |
| 121 | Forbat 2020 | Stepped wedge RCT | 12 | 1,700 | Hospital transfer/length of stay | Multicomponent (education, clinical guidance and team meetings) |
| 122 | Forster 2021 | Pilot/feasibility trial | 12 | 153 | Wellbeing | Multicomponent complex whole home intervention |
| 123 | Frändin 2016 | RCT | 24 | 332 | Activities of daily living, balance function, physical activity, falls related self-efficacy, wellbeing & cognition | Multicomponent (physical training, activity & professional support) |
| 124 | Froggatt 2020 | Pilot/feasibility trial | 8 | 32 | Intervention feasibility | Namaste Care |
| 125 | Gajewska 2022 | Before and after (one arm) | 1 | 29 | Nutrition | Nutritional supplements/dietary modification |
| 126 | Galik 2021 | Cluster RCT | 12 | 336 | Function, physical activity & BPSD | Multicomponent (education, training, goal setting, assessment of policies, assessment of environment & motivation of staff) |
| 127 | Garland 2021 | Cluster RCT | 29 | 713 | Palliative/end of life care | Multicomponent (training & practice resources) |
| 128 | Garland 2022 | Before and after | 4 | 691 | Medication management | Multicomponent (professional support, collective prescriptions, training & medication reviews) |
| 129 | Gattinger 2017 | RCT | 3 | 44 | Sleep | Multicomponent (technology & case conferencing) |
| 130 | Gemelli 2016 | Before and after (one arm) | 11 | 36 | Prescribing - sedatives & hypnotics | Dedicated professional support |
| 131 | Gencbas 2018 | Cluster RCT | 5 | 62 | Incontinence | Care bundle/pathway |
| 132 | Gillis 2016 | Cluster RCT | 2 | 168 | Skincare/integrity | Equipment/devices |
| 133 | Gillis 2019 | Before and after (one arm) | 3 | 65 | Agitation & behaviour | Multicomponent (therapeutic touch; music; meaningful activities) |
| 134 | Gine-Garriga 2020 | Pilot/feasibility trial | 4 | 31 | Sedentary behaviour | Multicomponent (training, familiarisation, goal setting, sessions with residents & families) |
| 135 | Gök Ugur 2017 | RCT | 1 | 64 | Depression | Music therapy |
| 136 | Gordon 2016 | Pilot/feasibility study | 11 | 251 | Quality of care | Multicomponent (education & technology assisted communication/case discussion) |
| 137 | Goyal 2021 | RCT | 6 | 120 | Anxiety & depression | Music therapy |
| 138 | Graham 2020 | Pilot/feasibility trial | 10 | 146 | Posture & mobility | Education/training |
| 139 | Gravenstein 2017 | Cluster RCT | 823 | 53,008 | Hospital transfer/length of stay | Pharmacological |
| 140 | Gravenstein 2021 | Cluster RCT | 737 | NR | Infection | Pharmacological |
| 141 | Guion 2018 | Non-randomised CT | 159 | 3,722 | Pain | Multicomponent (audit, feedback & professional support) |
| 142 | Gulla 2018 | RCT post-hoc analysis | 32 | 295 | Cognition, blood pressure & prescribing | Medication review |
| 143 | Gunst 2022 | RCT | 1 | 35 | Wellbeing | Technology |
| 144 | Gustavsson 2015 | Other: Quasi-experimental study using generalised linear modelling | 1 | 57 | Falls | Environmental modification |
| 145 | Guzmán 2016 | Other: Single subject design with multiple baselines across individuals | 3 | 10 | Mood & behaviour | Dance therapy |
| 146 | Haddad 2018 | Before and after (one arm) | 1 | 116 | Behavioural & Psychological Symptoms of Dementia | Environmental modification |
| 147 | Hahnel 2017 | RCT | 10 | 133 | Skincare/integrity | Pharmacological |
| 148 | Haines 2020 | Stepped wedge RCT | 15 | NR | Health outcomes | New model of care |
| 149 | Halek 2020 | Stepped wedge RCT | 12 | 465 | Behaviour that challenges | Multicomponent (training & case conferences) |
| 150 | Hanlon 2021 | Other: Quality improvement study | 25 | 167 | Prescribing - antimicrobials | Education/training |
| 151 | Hansen 2018 | Other: Single subject design with multiple baselines across individuals | 1 | 12 | Nutrition | Coloured plates |
| 152 | Harrison 2021 | RCT | 13 | 162 | Agitation | Music therapy |
| 153 | Hartshorn 2021 | Cluster RCT | 3 | 24 | Oral health | Education/training |
| 154 | Hashimoto 2017 | RCT | 5 | 75 | Cognition | Pharmacological |
| 155 | Hassan 2016 | Pilot/feasibility trial | 4 | 42 | Sarcopenia & function | Exercise |
| 156 | Henskens 2018a & 2018b | RCT | 11 | 87 | Quality of life & activities of daily living | Exercise |
| 157 | Henwood 2015 | Pilot/feasibility trial | 2 | 24 | Physical function | Exercise |
| 158 | Hermans 2018 | Before and after | 30 | 429 | Palliative/end of life care | Multicomponent (training & care planning) |
| 159 | Hewitt 2018 | Cluster RCT | 16 | 221 | Falls | Exercise |
| 160 | Hirdes 2020 | Other: Longitudinal evaluation of policy intervention | 1238 | 127,497 | Prescribing - antipsychotics | Quality improvement |
| 161 | Hjetland 2021 | Cluster RCT | 8 | 69 | Sleep | Environmental modification |
| 162 | Hmwe 2022 | Pilot/feasibility trial | 3 | 23 | Sleep & wellbeing | Acupressure |
| 163 | Hockley 2016 | Other: Research evaluation of practice development and sustainability initiatives | 42 | NR | Palliative/end of life care | Multicomponent (education, care planning, clinical guidance & professional support) |
| 164 | Hodl 2019 | Cluster RCT | 12 | 381 | Continence | Multicomponent (education & clinical guidance) |
| 165 | Hollaar 2017 | Non-randomised CT | 17 | 103 | Infection | Pharmacological |
| 166 | Hopkins 2017 | Other: Cluster-randomised crossover | 7 | 80 | Mood & sleep | Environmental modification |
| 167 | Hopper 2016 | Other: Quasi-experimental repeated-measures crossover design | 5 | 31 | Communication | Technology |
| 168 | Huey-Ming 2021 | Before and after (one arm) | 1 | 15 | Falls | Multicomponent (risk assessment, prevention, education & clinical guidance) |
| 169 | Hullick 2016 & 2022 | Before and after | 13 | NR | Hospital transfer/length of stay | Dedicated professional support |
| 170 | Hurley 2020 | Pilot/feasibility study | 3 | 35 | Physical activity & wellbeing | Multicomponent (individual care plans, review of physical environment & training) |
| 171 | Husebø 2019 & Gedde 2021 & Habiger 2021 | Cluster RCT | 33 | 428 | Quality of life | Multicomponent (communication, activities, clinical guidance & medication review) |
| 172 | Inch 2019 | Pilot/feasibility study | 6 | 40 | Falls, medications, quality of life, activities of daily living, mental state & adverse events | Dedicated professional support |
| 173 | Isaac & Parajuli 2021 | Before and after (one arm) | 5 | 74 | Agitation & behaviour | Music therapy |
| 174 | Iuliano 2021 | Cluster RCT | 60 | 7,195 | Fracture reduction & safety | Nutritional supplements/dietary modification |
| 175 | Jablonski 2018 | RCT | 9 | 101 | Oral health | Behavioural intervention with communication strategies |
| 176 | Jahanpeyma 2021 | RCT | 1 | 72 | Falls, balance & physical performance | Exercise |
| 177 | Jansen 2018 | Non-randomised CT | 2 | 143 | Life space utilisation | Exercise |
| 178 | Jarboe 2015 | Other: Quality improvement study | 1 | NR | Hospital transfer/length of stay | Communication tool |
| 179 | Jeong 2022 | Before and after | 2 | 188 | Delirium | Education/training |
| 180 | Johansson 2020 | Pilot/feasibility study | 1 | 46 | Oral health | Dedicated professional support |
| 181 | Johnen 2018 | Pilot/feasibility trial | 1 | 45 | Physical performance | Exercise |
| 182 | Jøranson 2021 | Cluster RCT | 10 | 60 | Sleep | Technology |
| 183 | Jordan 2015 | Stepped wedge RCT | 5 | 41 | Medication management | Medication review |
| 184 | Jordan 2017 | Pilot/feasibility trial | 3 | 17 | Functional independence | Multicomponent (coaching, education & feedback) |
| 185 | Jung 2018 | Non-randomised CT | 1 | 12 | Function, self-esteem & depression | Client-centred leisure activity programme |
| 186 | Junius-Walker 2021 | Cluster RCT | 44 | 773 | Deprescribing | Multicomponent (education & medication review) |
| 187 | Jupiter 2016 | Pilot/feasibility study | 1 | 10 | Cognition & quality of life | Technology |
| 188 | Juthani-Mehta 2016 | RCT | 21 | 185 | Bacteriuria | Pharmacological |
| 189 | Kalinowski 2015 | Cluster RCT | 12 | 747 | Functional performance | Multicomponent (training & guidance) |
| 190 | Kalinowski 2019 | Cluster RCT | 12 | 195 | Pain | Education/training |
| 191 | Kalu 2021 | RCT | 12 | 168 | Physical function | Exercise |
| 192 | Kane 2017 | Before and after | 85 | 36,717 | Hospital transfer/length of stay | Multicomponent (training, clinical support, care pathways & clinical guidance) |
| 193 | Kårefjärd 2019 | Before and after (one arm) | 3 | 59 | Quality of life | Pet therapy |
| 194 | Keating 2020 | Pilot/feasibility study | 12 | 75 | Behaviour & quality of life | Reminiscence |
| 195 | Kennedy 2015 | Pilot/feasibility trial | 40 | 5,478 | Prescribing - bone protection | Multicomponent (education, audit, feedback & QI) |
| 196 | Kim 2016 | Other: Quasi-experimental study design with non-equivalent control group | 1 | 30 | Sleep | Footbaths |
| 197 | Kim 2021a | Cluster RCT | 10 | 482 | Quality of care | Technology |
| 198 | Kim 2021b | Pilot/feasibility study | 3 | 40 | Cognition & mental health | Multicomponent (music, exercise & activities) |
| 199 | Kirkham 2019 | Stepped wedge RCT | 10 | NR | Prescribing - antipsychotics | Multicomponent (education & interdisciplinary meetings) |
| 200 | Klotz 2018 | RCT | 14 | 219 | Oral health | Multicomponent (education & technology) |
| 201 | Kohler 2018 | Stepped wedge RCT | 7 | 140 | Continence | Multicomponent (education & interdisciplinary meetings) |
| 202 | Kok 2018 | Before and after | 2 | 145 | Quality of life | Environmental modification |
| 203 | Könner 2015 | Cluster RCT | 12 | 747 | Pain | Education/training |
| 204 | Kovach 2019 | Before and after (one arm) | 1 | 34 | Pain, agitation & thermal discomfort | Environmental modification |
| 205 | Kurt 2021 | Before and after | 1 | 38 | Loneliness | Music therapy |
| 206 | Kuru 2017 | Before and after | 2 | 65 | Quality of life | Laughter therapy |
| 207 | Kuru Alıcı 2018 | Pilot/feasibility study | 2 | 50 | Loneliness & death anxiety | Laughter therapy |
| 208 | Kushiro 2019 | RCT | 2 | 88 | Fever, constipation, inflammatory parameters & faecal microbiota | Pharmacological |
| 209 | Kütmeç Yilmaz 2021 | RCT | 1 | 45 | Quality of life | Muscle relaxation sessions |
| 210 | Kutschar 2020 | Cluster RCT | 15 | 509 | Pain | Education/training |
| 211 | Lamppu 2021 | Cluster RCT | 20 | 324 | Quality of life & resource use | Education/training |
| 212 | Laurence 2019 | Pilot/feasibility study | 2 | 32 | Oral health | Guidance |
| 213 | Lavallée 2019 | Pilot/feasibility study | 1 | 29 | Skincare/integrity | Care bundle/pathway |
| 214 | Lavigne 2017 & 2018 | RCT | 1 | 54 | Inflammation | Technology |
| 215 | Lee 2017 | Non-randomised CT | 3 | 27 | Bone density | Multicomponent (exercise & nutritional supplement) |
| 216 | Lee 2022 | RCT | 1 | 52 | Sleep & pain | Acupressure |
| 217 | Leguelinel-Blache 2020 | Before and after (one arm) | 1 | 41 | Medication management | Medication review |
| 218 | Lexow 2022 | RCT | 3 | 353 | Medication management | Dedicated professional support |
| 219 | Liao 2020 | Before and after | 11 | NR | Prescribing - antimicrobials | Technology |
| 220 | Linander 2020 | Other: Cluster-randomised crossover | 1 | 34 | Sleep | Environmental modification |
| 221 | Little 2019 | Other: Quality improvement study | 1 | 22 | Recognition of deteriorating resident | Multicomponent (early warning tool & clinical guidance) |
| 222 | Liu 2020 | Stepped wedge RCT | 12 | 1,700 | Palliative/end of life care | Multicomponent (interdisciplinary meetings, professional support & clinical guidance) |
| 223 | Livingston 2019 | RCT | 20 | 404 | Agitation | Education/training |
| 224 | Logan 2021 | Cluster RCT | 84 | 84 | Falls | Multicomponent (education, risk assessment, decision support & guidance) |
| 225 | Lok 2017 | Before and after | 1 | 80 | Depression & quality of life | Exercise |
| 226 | Low 2015 | RCT | 1 | 40 | Quality of life, agitation & sense of community | Intergenerational |
| 227 | Low 2016 | Pilot/feasibility trial | 1 | 18 | Intervention feasibility | Dance therapy |
| 228 | Machacova 2017 | RCT | 7 | 189 | Physical function | Exercise |
| 229 | Mackey 2019 | RCT | 1 | 357 | Falls with harm | Environmental modification |
| 230 | Madden 2015 | Pilot/feasibility trial | 11 | 80 | Antimicrobial resistance | Pharmacological |
| 231 | Mahlknecht 2019 | Before and after (one arm) | 9 | 120 | Medication management | Multicomponent (education & medication review) |
| 232 | Maidment 2018 | Pilot/feasibility study | 5 | 34 | Behavioural & Psychological Symptoms of Dementia | Multicomponent (education, professional support & training) |
| 233 | Mak 2022 | RCT post-hoc analysis | 16 | 231 | Falls | Exercise |
| 234 | Maltais 2018 & 2019a & 2019b | Pilot/feasibility trial | 7 | 91 | Nutrition & body composition | Exercise |
| 235 | Man 2020 | RCT | 38 | 178 | Sensory function | Dedicated professional support |
| 236 | Marmeleira 2018 | Pilot/feasibility study | 2 | 21 | Physical & cognitive function | Exercise |
| 237 | Martin 2019 | Cluster RCT | 6 | 326 | Hospital transfer/length of stay | Multicomponent (specialist review, shared decision-making & documentation) |
| 238 | Mbakile-Mahlanza 2020 | Cluster RCT | 9 | 40 | Resident experience of visits | Montessorri activities |
| 239 | McCabe 2019 | Cluster RCT | 33 | 684 | Consumer directed care | Multicomponent (training & professional support) |
| 240 | McCabe 2022 | Pilot/feasibility trial | 9 | 92 | Consumer directed care | Multicomponent (training & professional support) |
| 241 | McConeghy 2021 | Cluster RCT | 823 | 820 | Infection | Pharmacological |
| 242 | McCord 2020 | RCT | 2 | 24 | Cognition | Technology |
| 243 | McCreedy 2022 | RCT | 54 | 976 | Agitation | Music therapy |
| 244 | McDerby 2019 | Pilot/feasibility trial | 2 | 117 | Medication management | Dedicated professional support |
| 245 | McDermid 2022 | RCT | 16 | 103 | Person-centred care | Education/training using technology |
| 246 | McGilton 2017 | Pilot/feasibility study | 1 | 12 | Quality of life & quality of care | Multicomponent (communication plan, training & provider support) |
| 247 | McMaughan 2016 | Before and after | 12 | 547 | Prescribing - antimicrobials | Multicomponent (training & decision-aid) |
| 248 | Meeks 2015 | Cluster RCT | 23 | 82 | Depression | Psychological therapy |
| 249 | Mestres 2015 | Before and after (one arm) | 70 | 127 | Medication management | Medication review |
| 250 | Middelstädt 2016 | RCT | 9 | 71 | Cognition, quality of life, behavioural symptoms & activities of daily living | Cognitive stimulation |
| 251 | Millett 2021 | Pilot/feasibility trial | 1 | 24 | Cognition & psychosocial health | Music therapy |
| 252 | Mitchell 2018 | Cluster RCT | 64 | 402 | Advance care planning | Education/training using technology |
| 253 | Mitchell 2020, Loomer 2021, Moyo 2022 | Cluster RCT | 28 | 426 | Prescribing - antimicrobials | Multicomponent (education, feedback & management algorithms) |
| 254 | Mitchell 2021 | Cluster RCT | 360 | 111,961 | Palliative/end of life care | Education/training using technology |
| 255 | Mitolo 2017 | Non-randomised CT | NR | 30 | Cognition | Route learning |
| 256 | Miyazaki 2020 | RCT | 1 | 46 | Cognition & physical function | Drumming communication programme |
| 257 | Mody 2015 | RCT | 12 | 418 | Infection | Multicomponent (education, equipment & infection surveillance) |
| 258 | Mody 2021 | Cluster RCT | 6 | 245 | Infection | Multicomponent (education, equipment, feedback & infection surveillance) |
| 259 | Moniz-Cook 2017 | Cluster RCT | 63 | 832 | Behaviour that challenges | Multicomponent (education, clinical guidance & decision-support delivered by technology) |
| 260 | Morales 2017 | Before and after (one arm) | 1 | 12 | Hand hygiene | Multicomponent (education & equipment) |
| 261 | Morgan 2020 | Before and after | 74 | 75,414 | Infection | Policy intervention |
| 262 | Mouton 2017 | Pilot/feasibility study | 2 | 21 | Physical activity | Exercise |
| 263 | Moyle 2019 | Pilot/feasibility trial | 5 | 35 | Anxiety, agitation & aggression | Doll therapy |
| 264 | Mulasso 2015 | RCT | 6 | 104 | Mobility & balance | Exercise |
| 265 | Nace 2020 | Other: Quality improvement study | 25 | NR | Prescribing - antimicrobials | Education/training |
| 266 | Naczk 2020 | RCT | 1 | 20 | Strength | Technology |
| 267 | Nagata 2016 | RCT | 1 | 88 | Bowel management & infection control | Pharmacological |
| 268 | Nagayama 2016 | Pilot/feasibility trial | 12 | 54 | Quality of life & activities of daily living | Occupational therapy focused on goal setting & meaningful occupation |
| 269 | Nam 2016 | Before and after | 4 | 41 | Oral health | Exercise |
| 270 | Namasivayam-MacDonald 2017 | Before and after (one arm) | 1 | 7 | Oral health | Exercise |
| 271 | Nawrat-Szoltysik 2018 & 2019 | RCT | 4 | 83 | Physical activity & quality of life | Exercise |
| 272 | Nishiura 2018 | Before and after (one arm) | 1 | 2 | Behavioural & Psychological Symptoms of Dementia | Technology |
| 273 | Obayashi 2020 | Non-randomised CT | 6 | 78 | Social participation | Technology |
| 274 | Okan 2022 | RCT | 1 | 40 | Vitamin D levels | Environmental modification |
| 275 | Okkels 2021 | Cluster RCT | 1 | 52 | Quality of life | Nutritional supplements/dietary modification |
| 276 | Olsen 2016 | Cluster RCT | 10 | 58 | Mood & behaviour | Pet therapy |
| 277 | Onieva-Zafra 2018 | Pilot/feasibility study | 1 | 19 | Anxiety & depression | Music therapy & reminiscence |
| 278 | O'Sullivan 2022 | Cluster RCT | 10 | 162 | Apathy | Technology |
| 279 | Overgaard 2022 | Cluster RCT | 14 | 243 | Oral health | Multicomponent (education, assessment, prevention, care planning & documentation) |
| 280 | Papadopoulos 2022 | RCT | 9 | 33 | Wellbeing | Technology |
| 281 | Park 2015 | Before and after (one arm) | 1 | 40 | Oral health | Evidence-based algorithm |
| 282 | Park 2019 | RCT | 1 | 37 | Sleep & stress | Hand massage |
| 283 | Parsons 2017 & Elia 2018 | RCT | 53 | 104 | Nutrition | Nutritional supplements/dietary modification |
| 284 | Pasay 2019 | Cluster RCT | 88 | 1,258 | Prescribing - antimicrobials | Multicomponent (education & clinical guidance) |
| 285 | Pasina 2016 | Pilot/feasibility study | 10 | 272 | Prescribing - psychotropic medicines | Education/training |
| 286 | Pasquel 2015 | RCT | 2 | 150 | Medication management | Pharmacological |
| 287 | Passmore 2018 | RCT | 1 | 22 | Life satisfaction | Exercise |
| 288 | Pedersen 2018 | RCT | 52 | 648 | Hospital transfer/length of stay | Dedicated professional support |
| 289 | Pepera 2021 | RCT | 3 | 40 | Haemodynamic & physical function | Exercise |
| 290 | Pérez-Ros 2019 | RCT | 1 | 119 | Cognition, emotion & function | Music therapy |
| 291 | Peterson 2016 | Cluster RCT | 3 | NR | Infection | Pharmacological |
| 292 | Pinazo-Clapes 2020 | Pilot/feasibility trial | 2 | 145 | Prescribing - psychotropic medicines | Education/training |
| 293 | Rantz 2021 | Other: Longitudinal evaluation of policy intervention | 473 | NR | Quality of care | Dedicated professional support |
| 294 | Ray 2017 | Before and after (one arm) | 3 | 132 | Agitation & depression | Music therapy |
| 295 | Rezola-Pardo 2019, 2020a, 2020b, 2022 | RCT | 9 | 85 | Physical & cognitive performance, mood, quality of life & frailty | Exercise |
| 296 | Richter 2019 | Cluster RCT | 37 | 1,153 | Prescribing - antipsychotics | Multicomponent (education & medication review) |
| 297 | Riggs 2020 | Before and after | 2 | 30 | Oral health | Multicomponent (education & professional support) |
| 298 | Roets-Merken 2018 | Cluster RCT | 30 | 89 | Social participation | Self-management programme |
| 299 | Rolland 2016 | Non-randomised CT | 175 | 6,275 | Quality indicators, functional decline & resource use | Multicomponent (audit, feedback & professional support) |
| 300 | Rolland 2020 | Cluster RCT | 64 | 1,428 | Hospital transfer/length of stay | Multicomponent (screening & multidisciplinary team meetings) |
| 301 | Romøren 2017 | Stepped wedge RCT | 30 | 296 | Hospital transfer/length of stay | Education/training |
| 302 | Roos 2016 | Cluster RCT | 12 | 80 | Empowerment | Education/training |
| 303 | Rostad 2018 | Cluster RCT | 16 | 121 | Pain | Structured assessment tool |
| 304 | Roughead 2022 | RCT | 39 | 248 | Medication management | Dedicated professional support |
| 305 | Rutten 2022 | Cluster RCT | 16 | 693 | Prescribing - antimicrobials | Technology |
| 306 | Ryuichi 2021 | Before and after (one arm) | 1 | 96 | Hospital transfer/length of stay | Technology |
| 307 | Saal 2019 | Pilot/feasibility trial | 7 | 129 | Social participation | Multicomponent (facilitation, education, peer mentoring & practice guidance/resources) |
| 308 | Sackley 2015 & 2016 | Cluster RCT | 228 | 1,042 | Functional activity | Multicomponent (education, adaptations to environment plus professional input) |
| 309 | Sado 2020 | Pilot/feasibility trial | 23 | 57 | Cognition | Psychological therapy |
| 310 | Saevareid 2019 | Cluster RCT | 8 | 154 | Palliative/end of life care | Multicomponent (education, training & care planning) |
| 311 | Sahin 2022 | RCT | 1 | 72 | Empowerment | Exercise |
| 312 | Saint-Bryant 2020 | Pilot/feasibility trial | 12 | 19 | Adjustment to care | Activities to engage with new residents |
| 313 | Samefors 2020 | Cluster RCT | 3 | 42 | Vitamin D levels | Environmental modification |
| 314 | Sampson 2020 | Pilot/feasibility study | 3 | 28 | Agitation | Education/training |
| 315 | Sampson 2021 | Pilot/feasibility trial | 14 | 237 | Early detection of illness | Multicomponent (early warning tool, care pathways, communication tool & professional support) |
| 316 | Sanchez 2021 | Before and after | 519 | 96,216 | Medication management | Guidance |
| 317 | Santagata 2021 | RCT | 2 | 52 | Behavioural & psychological symptoms of dementia, caregiver burden & delirium | Doll therapy |
| 318 | Santamaria 2018 | RCT | 40 | 288 | Skincare/integrity | Equipment/devices |
| 319 | Saredakis 2020 | Non-randomised CT | 3 | 46 | Intervention feasibility | Technology |
| 320 | Saredakis 2021 | Pilot/feasibility study | 1 | 17 | Intervention feasibility | Technology |
| 321 | Seemer 2021 & 2022 | Before and after (one arm) | 2 | 50 | Nutrition | Nutritional supplements/dietary modification |
| 322 | Seleskog 2018 | Pilot/feasibility study | 2 | 37 | Oral health | Multicomponent (education & specialist input) |
| 323 | Shaw 2018 | RCT post-hoc analysis | 10 | NR | Prescribing - antipsychotics | Education/training |
| 324 | Shin 2015 | Before and after (one arm) | 1 | 51 | Mood, behaviour & social interactions | Doll therapy |
| 325 | Sjögren 2022 | Before and after | 6 | 292 | Resident experience & person-centeredness of the environment | Education/training |
| 326 | Sloane 2020 | Cluster RCT | 27 | 2,754 | Prescribing - antimicrobials | Multicomponent (training, guidelines & information for residents & families) |
| 327 | Sluggett 2020a & 2020b | Cluster RCT | 8 | 242 | Medication management, falls & hospital transfer | Medication review |
| 328 | Smeets 2021 | Cluster RCT | 13 | 380 | Prescribing - psychotropic medicines | Multicomponent (education & medication review) |
| 329 | Sprangers 2015 | Before and after | 1 | 26 | Communication | Education/training |
| 330 | Stacpoole 2015 | Other: Action research study | 6 | 37 | Behaviour & pain | Namaste Care |
| 331 | Stensvik 2022 | Cluster RCT | 17 | 309 | Neuropsychiatric symptoms | Multicomponent (assessment & multidisciplinary case discussion) |
| 332 | Stephens 2020 | Pilot/feasibility study | 1 | 12 | Activities of daily living, posture & quality of life | Equipment/devices |
| 333 | Stow 2015 | Pilot/feasibility trial | 6 | 93 | Nutrition | Nutritional supplements/dietary modification |
| 334 | Strauven 2019 | Cluster RCT | 54 | 1,804 | Medication management | Dedicated professional support |
| 335 | Sullivan 2017 | RCT | 11 | 93 | Depression & wellbeing | Story sharing |
| 336 | Sultana 2021 | Before and after (one arm) | 1 | 24 | Intervention feasibility | Technology |
| 337 | Surr 2020 & 2021 | Cluster RCT | 50 | 726 | Behaviour & care outcomes | Multicomponent (training, observations of care practice, feedback & action planning) |
| 338 | Suzuki 2019 | RCT | 13 | 80 | Incontinence | Technology |
| 339 | Swales 2022 | Pilot/feasibility trial | 1 | 11 | Intervention feasibility | Exercise |
| 340 | Tadrous 2020 | Cluster RCT | 40 | 5,363 | Prescribing - antipsychotics | Dedicated professional support |
| 341 | Tappen 2020 | RCT | 15 | 192 | Hospital transfer/length of stay | Decision aid |
| 342 | Taylor 2015 | Cluster RCT | 9 | 65 | Mobility | Exercise using technology |
| 343 | Taylor 2018 | Pilot/feasibility study | 1 | 12 | Mobility | Education/training |
| 344 | Teesing 2021 | Cluster RCT | 33 | NR | Hand hygeine | Multicomponent (education & training) |
| 345 | Telenius 2015a & 2015b | RCT | 18 | 170 | Balance | Exercise |
| 346 | Temkin-Greener 2017a & 2017b | RCT | 634 | 125,316 | Palliative/end of life care | Multicomponent (training & professional support) |
| 347 | Ter Wee 2016 | RCT | NR | 67 | Intervention safety | Nutritional supplements/dietary modification |
| 348 | Testad 2016 | Cluster RCT | 24 | 274 | Restraint use | Education/training |
| 349 | Thodberg 2016a & 2016b | RCT | 4 | 100 | Sleep & wellbeing | Pet therapy |
| 350 | Travers 2017 | Pilot/feasibility trial | 4 | 19 | Mood & quality of life | Multicomponent (individualised assessment, training & activities intervention) |
| 351 | Treusch 2015 | RCT post-hoc analysis | 18 | 117 | Apathy | Biographical mobilisation intervention |
| 352 | Tropea 2022 | Cluster RCT | 24 | 1,304 | Hospital transfer/length of stay | Education/training |
| 353 | Tsai 2018 | Pilot/feasibility study | 2 | 12 | Functional independence | Education/training |
| 354 | Tsugawa 2020 | Pilot/feasibility study | 1 | 37 | Cognition & physical function | Exercise |
| 355 | Tylner 2016 | Other: Cluster-randomised crossover | 5 | 28 | Nutrition | Nutritional supplements/dietary modification |
| 356 | Valiani 2017 | Pilot/feasibility study | 1 | 12 | Physical performance | Exercise using technology |
| 357 | Van Bogaert 2016 | RCT | 2 | 60 | Depression, cognition & behaviour | Reminiscence |
| 358 | van Dam 2020 | RCT | 17 | 95 | Pain, quality of life & behaviour | Pharmacological |
| 359 | Van den Block 2019, 2020 & Miranda 2021 | Cluster RCT | 78 | 488 | Palliative/end of life care | Multicomponent (education, training, care planning, multidisciplinary meetings & clinical guidance) |
| 360 | Van Haitsma 2015 | RCT | 1 | 130 | Mood & behaviour | Multicomponent (activities & psychosocial) |
| 361 | van Leen 2018 | RCT | 21 | 206 | Skincare/integrity | Equipment/devices |
| 362 | van Welie 2016 | Before and after (one arm) | 3 | 197 | Medication management | Education/training |
| 363 | van Wietmarschen 2020 | Other: Participatory evaluation | 3 | 93 | Side effects | Pharmacological |
| 364 | Van Wymelbeke 2016 | Non-randomised CT | 8 | 87 | Nutrition | Nutritional supplements/dietary modification |
| 365 | Van Wymelbeke 2020 | Other: 2x2 full factorial within-subject design | 6 | 89 | Nutritional intake/enjoyment | Nutritional supplements/dietary modification |
| 366 | Veleva 2020 | RCT | 3 | 78 | Wellbeing | Environmental modification |
| 367 | Verreault 2018 | Non-randomised CT | 4 | 193 | Palliative/end of life care | Multicomponent (education, communication & facilitation) |
| 368 | Viscogliosi 2017 | Other: Randomised trial, 3 interventions, no control | 1 | 75 | Agitation | Pharmacological |
| 369 | Visscher 2020 | Pilot/feasibility study | 2 | 15 | Nutrition | Nutritional supplements/dietary modification |
| 370 | Visser 2021 | Before and after (one arm) | 13 | 67 | Medication management | Deprescribing guidance/protocol |
| 371 | Walker 2016 | Pilot/feasibility trial | 6 | 52 | Falls | Multicomponent (education, training & clinical guidance) |
| 372 | Walker 2020 | Non-randomised CT (cluster) | 41 | 3,400 | Prescribing - supplements | Multicomponent (education & quality improvement) |
| 373 | Wallace 2016 | Before and after (one arm) | 5 | 208 | Oral health | Dedicated professional support |
| 374 | Wang 2018 | Pilot/feasibility trial | 14 | 209 | Infection | Pharmacological |
| 375 | Watson 2019 | Other: Randomised crossover trial | 6 | 49 | Agitation | Aromatherapy |
| 376 | Wauters 2021 | Pilot/feasibility study | 3 | 148 | Medication management | Multidisciplinary review |
| 377 | Weatherall 2019 | Before and after | 790 | NR | Hospital transfer/length of stay | Dedicated professional support |
| 378 | Weintraub 2018 | Cluster RCT | 13 | 219 | Oral health | Education/training |
| 379 | Werner 2017 | Cluster RCT | 2 | 117 | Depression | Music therapy |
| 380 | Wesenberg 2019 | Other: Within-subject design with two conditions | 2 | 19 | Social interaction, emotional expression & behavioural & psychological symptoms | Pet therapy |
| 381 | Westerhof 2018 | RCT | 2 | 81 | Depression | Autobiographical intervention |
| 382 | Whitney 2017 | Pilot/feasibility trial | 9 | 18 | Falls | Multicomponent (dementia care mapping, geriatric assessment, occupational therapy & exercise) |
| 383 | Wijnen 2015 | RCT | 1 | 30 | Vitamin D levels | Pharmacological |
| 384 | Wilchesky 2018 | Pilot/feasibility study | 3 | 44 | Medication management | Knowledge exchange |
| 385 | Williams 2016 | Before and after (one arm) | 4 | 24 | Physical activity | Multicomponent (education & technology) |
| 386 | Wilson 2019 | Other: Quality improvement study | 2 | NR | Hydration | Quality improvement |
| 387 | Wogamon 2016 | Before and after (one arm) | 1 | NR | Skincare/integrity | Education/training |
| 388 | Woloszyn 2021 | RCT | 5 | 165 | Physical activity | Dance therapy |
| 389 | Wren 2016 | Before and after (one arm) | 1 | 9 | Life satisfaction & quality of life | Life review |
| 390 | Wylie 2017 | Pilot/feasibility trial | 6 | 43 | Intervention feasibility | Dedicated professional support |
| 391 | Yamamoto 2019 | RCT | 2 | 107 | Immunoglobulin levels | Pharmacological |
| 392 | Yap 2022 | Cluster RCT | 9 | 992 | Skincare/integrity | Care practice |
| 393 | Yasuda 2017 | Before and after (one arm) | 1 | 40 | Quality of life | Education/training |
| 394 | Yeşilyaprak 2016 | Before and after | 1 | 21 | Physical function | Exercise using technology |
| 395 | Yücel 2019 | RCT | 1 | 30 | Comfort & anxiety | Therapeutic touch & massage |
| 396 | Zimmerman 2020 | Cluster RCT | 14 | 2,152 | Oral health | Education/training |

Abbreviations: RCT – randomised controlled trial; NR – not reported

**Appendix 5**

**Supplementary Table 6: Summary of aim, intervention & control (if applicable) for each included dataset**

|  | **Study ID**  **(Author, Year(s))** | **Aim** | **Intervention** | **Control (if applicable)** |
| --- | --- | --- | --- | --- |
| 1 | Abraham 2019 | To evaluate the effectiveness of two versions of a guideline and theory-based multicomponent intervention to reduce physical restraints | Guideline-based multicomponent intervention | Usual care |
| 2 | Acaroz Candan 2019 | To determine the effect of two neuromuscular electrical stimulation periods on quadriceps strength and functional performance | LNMES – neuromuscular electrical stimulation with long stimulation period | SNMES – neuromuscular electrical stimulation with short stimulation period |
| 3 | Ailabouni 2019 | To examine the feasibility of implementing a deprescribing intervention that utilises a patient-centred pharmacist-led intervention model | Targeted deprescribing of anticholinergic and sedative medicines – doses reduced twice-weekly | N/A |
| 4 | Almeida 2022 | To determine if behavioural activation delivered by trained staff decreases prevalence of clinically significant symptoms of depression | Behavioural activation training | Usual care |
| 5 | Alp 2021 | To find out the effects of therapeutic touch on comfort and anxiety | Therapeutic touch - using touch for emotional comfort, 20 minutes of therapeutic touch over four days | Usual care |
| 6 | Álvarez Barbosa 2018 | To compare the responses to an exercise programme performed on a vibratory device and to the same exercise programme performed without vibration on lower limb performance, functional dependence and quality of life | Whole-body vibration, 3 sessions per week | Two control groups: non-vibration and usual care |
| 7 | Anderson 2018 | To examine the feasibility of providing group-based cognitive behavioural therapy for depression and anxiety | Cognitive Behavioural Therapy sessions 2-hours once weekly | Usual care |
| 8 | Arendts 2018 | To evaluate nurse practitioner care on hospital transfers and resident quality of life | Nurse Practitioners working with General Practitioners, using a best practice guide | Usual care |
| 9 | Arnold 2021 | To investigate whether a tailored intervention that improves knowledge about Urinary Tract Infection and communication skills in nursing home staff influences antibiotic prescriptions for Urinary Tract Infection | Nursing Home Staff attended 75 min sessions over 8 weeks of interactive educational sessions to learn how to distinguish asymptomatic bacteriuria from UTI and learn how to use a dialogue tool | Usual care |
| 10 | Arrieta 2018, 2019a, 2019b, 2020, 2022 | To evaluate the effect of multicomponent exercise on physical function, frailty and related adverse outcomes | Multicomponent physical exercise programme on anthropometry, physical function, and physical activity | Routine low-intensity activities |
| 11 | Ashcraft 2017 | To compare customised and standardised SBAR (situation, background, assessment, response) tools to prevent resident transfer | Customised SBAR tool, one hour teaching session for nurses on use of SBARc. Documentation of clinical communication on nurse provider log and electronic health record SBARs | Reminder to document clinician communication on nurse provider logs and on the electronic health record SBARs |
| 12 | Baandrup 2021 | To evaluate the effect and applicability of a dynamic light intervention to improve rest-activity patterns in cognitively impaired older adults | Specialized dynamic light intervention was installed in the private apartments of each resident in the intervention arm, no alteration to light systems in common rooms | Conventional lighting |
| 13 | Bae 2020 | To explore the associations between an ambient scent environment and residents’ wellness | Lavender reed diffuser placed at bedside | Non-scented reed diffuser placed bedside |
| 14 | Bagci 2020 | To investigate the effect of therapeutic touch on sleep quality | Two intervention groups: Therapeutic touch - 10 minute between 18:00-22:00 for 3 consecutive days; Placebo - Therapeutic touch without explaining what is happening to resident | Usual care |
| 15 | Bailey 2017 | To conduct a randomized controlled trial of an intervention to reduce depressive symptoms in residents with dementia | Question-asking- reading, reminiscence, and cognitive-behavioural therapy plus environmental supports and individualized behavioural activity programs | Ongoing nursing home activities (e.g., singing, bible study, and bingo) |
| 16 | Baker 2017 | To evaluate the outcomes of the Avondale Intergenerational Design Challenge | Avondale Intergenerational Design Challenge intervention for 13-15yr-old technology students to interact with residents, design and present a product for their use | N/A |
| 17 | Ballard 2016, Rajkumar 2016 | To evaluate the impact of antipsychotic review, social interaction, and exercise, in conjunction with person-centred care, on antipsychotic use, agitation, and depression in people with dementia | Person centred care intervention plus antipsychotic medication review, social interaction with pleasant activities intervention and/or exercise intervention | Person centred care intervention |
| 18 | Ballard 2018 | To evaluate the efficacy of a person-centred care and psychosocial intervention incorporating an antipsychotic review, on QoL, agitation, and antipsychotic use in people with dementia | All homes allocated to the intervention received staff training in person-centred care and social interaction and education regarding antipsychotic medications (antipsychotic review), followed by ongoing delivery through a care staff champion model | Usual care |
| 19 | Balsom 2020 | To assess the effectiveness of a collaborative pharmacist-led deprescribing programme | A pharmacist-led deprescribing intervention | Usual care |
| 20 | Barbe 2019 | To investigate the impact of professional brushing performed every 2 weeks by a dental nurse on the number of teeth, incidence of root caries, and further short‐term oral health parameters, compared with residents whose oral hygiene was performed or supervised by staff according to standards of care | Professional cleaning of teeth in resident rooms by dental nurse every 2 weeks | Usual care |
| 21 | Barbe 2020 | To investigate the efficacy of professional brushing using a specialized brush every three weeks and its impact on outcomes | Professional dental cleaning at baseline and then dental nurse cleaned teeth every 3 weeks over and above standard dental care | N/A |
| 22 | Baron 2015 | To evaluate the success of a programme of advance care planning (ACP) education for nursing home staff by examining its effect on staff knowledge, ACP practice within the home and end-of-life hospital admission rates from the nursing home | Programme of advance care planning education for nursing home staff | Usual care |
| 23 | Barrett 2019 | To evaluate the short-term effect of MARIO, a social robot, on quality of life, depression, and perceived social support in persons with dementia and evaluates their acceptability of MARIO | Sessions to engage with MARIO (social robot) three times per week for four weeks, facilitated by a researcher | N/A |
| 24 | Barthalos 2016 | To evaluate the changes in quality of life, attitude to aging, assertiveness, physical fitness and body composition through a 15-week organized resistance training based physical activity programme | Two intervention groups: age and skill appropriate resistance training twice weekly for 45 minutes and training activity plus weekly lectures and discussions on ageing and quality of life | Usual care |
| 25 | Bassi 2018 | To contribute to extant literature through the investigation of the quality of experience associated with horticultural versus occupational activities | Weekly horticultural and occupational sessions for two six-week cycles | N/A |
| 26 | Batchelor-Murphy 2015 | To test a web-based version of a dementia feeding skills educational intervention, and examine the efficacy of the approach | Web-based educational intervention on dementia feeding skills for nursing home staff with group coaching | Usual care |
| 27 | Bauer 2015 | To evaluate the impact of Snoezelen delivered in a dedicated room by an activity therapist compared to ‘common best practice’ interventions as provided by care staff (nurses and personal care assistants), in allaying the dementia related behaviours of wandering and restlessness | A qualified diversional therapist was solely responsible for the organization and delivery of the Snoezelen in this study, Snoezelen sessions were implemented on the basis of the diversional therapist’s knowledge of the resident and prior experience | Usual care |
| 28 | Bautrant 2019 | To determine whether environmental rearrangements of the home can affect disruptive behavioural and psychological symptoms of dementia in residents with dementia | Environmental modifications to enhance day/night orientation | N/A |
| 29 | Beaupre 2019 & 2020 | To compare functional outcomes at 3 months after hip fracture between residents who participated in a 10-week outreach rehabilitation programme and those who received usual post-fracture care | Three rehabilitation sessions per week after hip fracture surgery | Usual care |
| 30 | Beeckman 2019 & Anrys 2019 | To compare the effectiveness and cost of static air support surfaces versus alternating air pressure support surfaces in a population at high risk for pressure ulcers | Static air support surfaces: a Repose static air mattress, a seat cushion, and a heel wedge or a foot protector | Alternating air pressure support surfaces |
| 31 | Bellini 2015 | To compare the impact of standard precautions either alone (control) or combined with screening of residents and decolonization of carriers (intervention) to control MRSA | Screening of residents for MRSA and decolonization of carriers | Usual care |
| 32 | Benigni 2018 | To assess and compare the efficacy of three different devices in reducing leg oedema | Comparison of three devices for reducing stasis oedema (Veinoplus vs Circaid Juxtafit vs Rosidal K) | No device |
| 33 | Berkheimer 2017 | To compare the effects of a Snoezelen programme and an exercise programme on agitation in residents with dementia | Snoezelen therapy 3 x 30 minutes per week, Exercises 3 x 30 minutes per week | Usual care |
| 34 | Bertoncello 2021 | To assess the effectiveness of an individually tailored physical exercise intervention on mobility and functional decline | Tailored physical exercise intervention (45 minutes x3/week) | N/A |
| 35 | Bischoff 2021 | To design a multi-component training programme which improves physical functioning and psychosocial wellbeing and to evaluate the feasibility of this intervention | Multicomponent cognitive-motor training (including dual-task, dynamic balance, endurance and strength exercises), twice per week for 45–60 minutes | Usual care |
| 36 | Blaak 2015 | To assess whether acidic skin care generates positive clinical, biophysical, and microbiological effects in aged skin | Skin care with pH 4.0 | Skin care with pH 6.0 |
| 37 | Blair 2016 | To evaluate the clinical effectiveness and practical viability of groups loosely based on cognitive behaviour therapy principles for residents with anxiety and/or depressive symptoms | Weekly psychological therapy sessions | N/A |
| 38 | Blytt 2017 | To investigate the effects of pain treatment on sleep in residents with dementia and depression | Stepwise pain treatment: paracetamol, buprenorphine | Placebo |
| 39 | Boere 2021 | To evaluate whether C reactive protein point-of-care testing safely reduces antibiotic prescribing for lower respiratory tract infections | Point of care CRP test machines made available to responsible nursing home physicians | Usual care |
| 40 | Bökberg 2019 | To evaluate whether a palliative care intervention had any influence on the perceived quality of life of older persons | Knowledge-based palliative care intervention implemented with various staff members working in the care home and front-line leaders | Cross-over design |
| 41 | Boockvar 2016 | To undertake pilot-testing of a multicomponent delirium prevention intervention adapted from the Hospital Elder Life Program (“HELP-LTC”) | Multicomponent delirium risk reduction intervention delivered by certified nursing assistants targeting common risk factors | Usual care |
| 42 | Boockvar 2020 | To assess applicability of Hospital Elder Life Program adapted to long-term care and its outcomes on delirium and hospitalisation | A multicomponent intervention targeting delirium risk factors of cognitive impairment, immobility, dehydration, and malnutrition. Delivered by trained nurse (independent from nursing home nurses) twice daily for duration of illness, up to max 3 weeks | Usual care |
| 43 | Boogaard 2018 | To assess the effect of two feedback strategies on perceived quality of end-of-life care and comfort in dying residents with dementia | Generic feedback vs patient-specific feedback vs control | Usual care |
| 44 | Booth 2021 | To determine the clinical effectiveness of transcutaneous posterior tibial nerve stimulation to treat urinary incontinence in residents and to determine the associated costs of the treatment | Transcutaneous posterior tibial nerve stimulation | Sham stimulation |
| 45 | Boström 2016, Sondell 2018, 2019a, 2019b, Toots 2019 | To evaluate the effect of a high-intensity functional exercise programme on residents with dementia | High-intensity functional exercise programme on depressive symptoms in people with dementia | Non-exercise control activity |
| 46 | Bourdon 2021 | To determine whether environmental enrichment applied to gardens could produce beneficial effects on clinical markers of function for residents with dementia | Incentive to visit conventional sensory garden, incentive to visit enriched garden | No incentive to visit either garden |
| 47 | Brännström 2016 | To compare the effects of the Liverpool Care Pathway for the Dying Patient and usual care on residents’ symptom distress and well-being during the last days of life | Introduction of the Liverpool Care Pathway for the Dying Patient | Usual care |
| 48 | Bravo-Jose 2019 | To develop a clinical protocol for the use of antipsychotic drugs in dementia residents with behavioural disturbances that includes prescribing and deprescribing criteria | Prescribing then deprescribing antipsychotics in resident with dementia | N/A |
| 49 | Brazil 2018 | To evaluate the effectiveness of advance care planning with family carers in dementia care homes | A trained advance care planning facilitator, family education, family meetings, documentation of advance care plan decisions, and orientation of GPs and nursing home staff to the intervention | Usual care |
| 50 | Brett 2017, 2019 & 2021 | To evaluate the effects of an exercise intervention on physical performance and reported fall incidents among individuals living with dementia | Two intervention groups: Physical exercise for 45 minutes, weekly and Physical exercise for 15 minutes, three times a week | Usual care |
| 51 | Brimelow 2022 | To assess the feasibility of using group-based fully immersive virtual reality in reducing behavioural and psychological symptoms in cognitively diverse residents | Virtual reality videos | N/A |
| 52 | Brodaty 2018 | To evaluate the sustained reduction of antipsychotic use for Behavioural and Psychological Symptoms in Dementia through a deprescribing intervention and education of health care professionals | Antipsychotic deprescribing protocol established with GPs, pharmacists, care nurses | N/A |
| 53 | Brooker 2016 | To reduce the inappropriate prescription and use of anti-psychotic medications, by providing person-centred approaches and evidence-based psycho-social interventions to support people experiencing Behavioural and Psychological Symptoms in Dementia | Focused Intervention Training and Support programme delivered by Dementia Practice Development Coaches and local education and training | N/A |
| 54 | Brown 2019 | To determine whether the programme decreased urine culturing and antibiotic prescribing rates and whether specific strategies of the programme were more or less likely to be adopted | The Urinary Tract Infection programme was designed by Public Health Ontario in consultation with long-term care home stakeholders to improve or change five practices. Nine strategies were identified to support these practice changes. | N/A |
| 55 | Brustio 2015 | To investigate the direct and indirect effects of physical intervention based on neuromotor training in mobility, basic activities of daily living and fear of falling and the feasibility of this training | Twice weekly 60-minute sessions of Neuromotor training involving combinations of balance, coordination and gait exercises | Usual care |
| 56 | Bryant 2020 | To investigate the feasibility and efficacy of an 8-week, volunteer-led behavioural activation intervention designed for depressed residents | Behavioural activation given by trained volunteers | N/A |
| 57 | Butler 2020 & Castro-Herrera 2021 | To determine whether a daily oral probiotic combination of *Lactobacillus rhamnosus GG* and *Bifidobacterium animalis subsp lactis BB-12* compared with placebo reduces antibiotic administration | Daily oral probiotic combination of Lactobacillus rhamnosus GG and Bifidobacterium animalis subsp lactis BB-12 | Placebo |
| 58 | Callegari 2022 | To examine whether Quality of Life in residents could be influenced by exposing physicians to an educational programme about Norwegian General Practice Nursing Home (NorGeP–NH) criteria, after receiving a lecture on psychotropic drug use in older adults, and requesting them to perform a structured medication chart review with NorGeP–NH criteria | Polypharmacy stewardship by educational intervention on physician responsible for residents with emphasis on psychotropic medications | Usual care |
| 59 | Cantarella 2018 | To measure the impact of Doll Therapy on people with severe dementia with a reliable, commonly used scale for assessing their Behavioural and Psychological Symptoms in Dementia, and the related distress in formal caregivers | Doll therapy in 20 consecutive sessions (5 times per week in afternoon) | Hand warmer in 20 consecutive sessions (5 times per week in afternoon) |
| 60 | Cateau 2021a | To see if an Individual Deprescribing Intervention targeted at residents with polypharmacy would reduce the number of potentially inappropriate medications per resident | Educational intervention for nursing home pharmacist on medication rationalisation | Usual care |
| 61 | Cateau 2021b | To assess effect of an interprofessional Quality Circle-Deprescribing Module | Quality Circle Deprescribing Module, researchers provided educational sessions and agreed deprescribing protocols for pharmacists | Usual care |
| 62 | Catiker 2021 | To develop a protective and improving protocol for foot health of the older people and to assess the effect of this protocol on foot problems, foot care knowledge levels and foot care behaviour | Foot care protocol - nurses and residents trained in foot care techniques | Usual care |
| 63 | Cavallini 2015 | To investigate the efficacy of self-help training on memory tasks they practiced and any transfer to other tasks | Self-help memory training programme | General cognitive stimulation activities |
| 64 | Cavallini 2021 | To evaluate whether socio-cognitive abilities can be improved by means of a theory of mind training conducted by nursing home’s operators | Conversation-based Theory of Mind training | Cognitive activities based on newspaper reading, crossword puzzles, and text writing |
| 65 | Çetinkaya 2019 | To assess the cognitive activity and satisfaction with life of older adults before and after ceramic painting | Group ceramic painting twice weekly for 8 weeks | Usual care |
| 66 | Chalhoub 2016 | To conduct a feasibility study on investigating the effectiveness of an alcohol-free essential oil mouthwash to reduce plaque accumulation and oral pathogen levels | Alcohol-free essential oil mouthwash used twice daily | Twice daily rinsing with tap water |
| 67 | Chambers 2022 | To evaluate the use of virtual learning collaboratives to support homes in implementing a quality improvement programme focused on reducing unnecessary urine culturing and antibiotic overprescribing | Virtual learning collaborative sessions on 'UTI Programme' attended by a nominated lead from each home | Passively exposed to provincial dissemination of electronic programme materials |
| 68 | Chiesi 2021 | To assess health improvements after the Biodanza intervention, a nonpharmacological dance movement-based treatment. | Dance movement-based treatment | N/A |
| 69 | Chou 2016 | To reduce resistance-to-care and combative behaviours in residents with dementia by eliciting their positive affect | Images from the International Affective Pictures System were used to elicit positive emotion | N/A |
| 70 | Chu 2016 & 2020 | To evaluate the feasibility and acceptability of a person-centred multifaceted walking intervention (MWI) and, second, assess the efficacy of the MWI to maintain the functional mobility, ADL function, and quality of life of residents with dementia | Multifaceted walking intervention a complex intervention incorporating person centred care to meet residents' physical, social and psychological needs | N/A |
| 71 | Churcher Clarke 2017 | To develop a group-based adapted mindfulness programme for people with mild to moderate dementia and to determine its feasibility and potential benefits | Ten sessions of adapted mindfulness | Usual care |
| 72 | Cichocki 2015 | To explore the effectiveness of a multi-faceted, low-threshold physical activity programme | Weekly 60-minute sessions of low-threshold physical activity | Invited to three social animation events |
| 73 | Clarke-O'Neill 2015 | To determine whether the severity of incontinence-associated dermatitis (IAD) among incontinence pad users varies between pad designs | A day-time variant and a night-time variant of each of the 4 main disposable pad designs on the market for moderate/heavy incontinence were tested: (1) insert pads with stretch pants; (2) 1-piece all-in-one diapers; (3) pull-up pants; and (4) belted/T-shape diapers | Cross-over design |
| 74 | Cohen 2019 | To examine concordance between advance directives and proxy care preferences among residents with advanced dementia and to determine the impact of an advance care planning video on concordance | Advance care planning video decision tool for proxies of nursing home residents with advanced dementia | Usual care |
| 75 | Colón-Emeric 2017 | To test whether a complexity science–based staff training intervention promoting high-quality staff interactions improves the impact of an evidence-based falls quality improvement programme | CONNECT programme followed by falls prevention quality improvement intervention | Falls prevention programme |
| 76 | Connolly 2015 & 2016 | To assess the effect of a complex, multidisciplinary intervention aimed at reducing avoidable acute hospitalisation | Gerontology nurse specialist (GNS)-led staff education, facility benchmarking, GNS resident review and multidisciplinary (geriatrician, primary-care physician, pharmacist, GNS and facility nurse) discussion of residents selected using standard criteria | Usual care |
| 77 | Conway 2015 | To evaluate the impact of a nurse-led telephone support service on a range of measures relating to the transfer of acutely unwell residents to the Emergency Department | A nurse-led telephone support service provided by an advanced practice nurse: the Aged Care Emergency Advanced Practice Nurse with experience in the emergency department and care of older people | Usual care |
| 78 | Cool 2018 | To investigate whether a geriatric intervention on quality of care reduced potentially inappropriate drug prescription | Geriatric education for nursing home staff | Usual care |
| 79 | Cordato 2018 | To evaluate the impact and cost-effectiveness of the Regular Early Assessment Post-Discharge (REAP) protocol of coordinated specialist geriatrician and nurse practitioner visits on rates of rehospitalization, hospital length of stay, and emergency department presentations for residents recently discharged from hospital | Regular Early Assessment Post-Discharge intervention of monthly coordinated specialist geriatrician and nurse practitioner assessments within participants’ homes for 6 months following hospital discharge | Usual care |
| 80 | Cordes 2021 | To determine the effects of a multicomponent chair-based exercise intervention on motor functions, cognition and well-being for residents who are unable to walk | Multicomponent chair-based exercise, adapted to resident performance level | Usual care |
| 81 | Couderc 2021 | To assess the impact of a personalized and adapted care pathway for residents suffering from cancer, in terms of access to oncological and ambulatory management, toxicity of cancer treatments and quality of life | Suspected cancer in resident referred straight to combined geriatric oncology clinic for simultaneous cancer work-up and CGA assessment +/- referral onward for oncology treatment | Usual care |
| 82 | Courel-Ibanez 2022 | To determine whether the benefits of long (24 weeks) and short (4 weeks) training programs persisted after short (6 weeks) and long (14 weeks) periods of inactivity in residents with sarcopenia | Vivifrail multicomponent exercise programme individually prescribed to residents depending on their functional capacity. Intervention group received Long training (24 weeks) of Vivifrail followed by 6 weeks of detraining. | Short training (4 weeks) and long detraining (14 weeks) |
| 83 | Crespy 2016 | To evaluate whether residents receiving the Promoting Positive Well-Being programme would experience a reduction in depressive systems as compared with others in a waitlist control group and diverse homes would find the programme acceptable and feasible to implement | The Promoting Positive Well-Being programme is a quality improvement intervention that features tools and strategies to assist homes in early identification, assessment, treatment, and monitoring of residents with depressive symptoms | N/A |
| 84 | Croonquist 2020 | To describe the effects for residents of monthly professional cleaning and individual oral hygiene instruction provided by registered dental hygienists, in comparison with daily oral care as usual | Monthly professional cleaning, individual oral hygiene instructions and information given by an registered dental hygienist | Usual care |
| 85 | Daneman 2021 | To determine whether peer comparison audit and feedback reporting for physicians reduces antibiotic overprescribing among residents | MyPractice (audit/QI data) report distributed to physicians as dynamic, online dashboard | MyPractice report distributed to physicians as static pdf |
| 86 | Davidson 2019 | To assess the scalability of the On-Time Pressure Ulcer Prevention intervention strategy | Implementation of the On-Time program: An information technology intervention designed to reduce rates of pressure ulcers by generating 4 core reports via homes’ electronic health record systems to identify residents with increased risk and monitor weekly changes in risk. Relies on nursing staff documenting variables in computer system and handover of identified at risk patients | Usual care |
| 87 | Davison 2017 | To evaluate the efficacy and acceptability of a psychological intervention based on acceptance and commitment therapy to improve symptoms of depression and anxiety | Twelve sessions of Acceptance and Commitment Therapy from a psychologist | Wait-list control group |
| 88 | Davison 2021 | To determine the effectiveness of Program to Enhance Adjustment to Residential Living in reducing depressive symptoms in newly admitted residents, compared to standard care | Programme to Enhance Adjustment to Residential Living - 1:1 session with resident and clinician aimed at augmenting adjustment period for new residents | Usual care |
| 89 | de Bruin 2020 | To assess the effects of stochastic resonance with whole body vibration combined with exergame dance training on functional and cognitive performance of care-dependent older adults | Vibration plate and dance exercise | Vibration plate (but insignificant frequency) and aktiv tramp (a trampoline) |
| 90 | De Luca 2016a | To evaluate the combined effects of the standard cognitive training in addition to web-based rehabilitation in people with dementia | Web-based cognitive training plus standard neurorehabilitation | Usual care |
| 91 | De Luca 2016b | To demonstrate the effectiveness of a novel telehealth-care model allowing a better management | Vital signs transmitted to e-health telemonitoring system | Usual care |
| 92 | de Pooter-Stijnman 2018 | To investigate the effect of caffeine reduction on sleep and challenging behavioural symptoms in residents with dementia | Gradual reduction in residents' caffeine intake to eliminate afternoon and evening caffeine | N/A |
| 93 | de Souto Barreto 2017 | To compare the effects of exercise with those of a structured non-physical intervention on ability to perform activities of daily living and physical and cognitive function of persons with dementia | Twice-weekly 1-hour exercise intervention | Twice-weekly 1-hour structured social activity |
| 94 | del Campo Cervantes 2019 | To evaluate the effect of a resistance training programme on the sarcopenia and functionality of residents | Resistance exercise program, thrice weekly | N/A |
| 95 | Dellinger 2020 | To evaluate the effectiveness of an intervention, which targets the cooperation of professions involved in residents’ medications | A new electronic platform available to healthcare professionals responsible for residents’ medications, incorporated a planned medicines reconciliation as part of implementing the database | Usual care |
| 96 | Demir 2022 | To assess the effects of proprioceptive exercises on balance and sensory function in the geriatric adults with Type 2 diabetes mellitus | Exercise regimen focused on lower limb proprioception and education on foot health | Diabetic education |
| 97 | Desborough 2020 | To determine the effectiveness (falls reduction) and cost-effectiveness, of a multi-professional medication review service | Multi-professional medication review done at 0 and 6 months by clinical pharmacist, GP and care home member of staff responsible for medication, with preparation undertaken by a pharmacy technician | Usual care |
| 98 | Devereaux 2016 | To test whether using a situation, background, assessment, recommendation (SBAR) tool specific to changes in condition for nurses to collect and report pertinent information improves nurses’ perception of nurse/physician communication and decreases the number of acute care hospitalizations | Condition specific SBAR tools introduced through nursing education sessions to staff | N/A |
| 99 | Dharmarajan 2016 | To compare the efficacy of Sliding Scale Insulin (control) and Basal-Bolus Insulin (intervention) therapies in residents | Basal-bolus insulin therapy | Sliding scale insulin therapy |
| 100 | Diegelmann 2018 | To examine the effect of a whole-ecology physical activity intervention programme on residents’ depressive symptoms using generalized linear mixed-models | Physical activity training program | Usual care |
| 101 | Dimori 2018 | To estimate the prevalence of sarcopenia and to collect preliminary data on the efficacy of a nutritional intervention either associated or not with a physical exercise program—depending on the features of residents— on muscle mass and functional status | Nutritional supplement for muscle mass recovery combined with supervised physical exercise rehab (40 minutes X 3 weekly) | Nutritional supplement only |
| 102 | Dolu 2019 | To investigate the impact of a nurse‐led sleep programme on the sleep quality and depressive symptomatology in cognitively intact residents | Nurse‐led sleep programme individualised by addressing sleep experience and sleep management strategies integrated with motivational interviewing techniques, four sessions 1-day per week for 1-hour | Usual care |
| 103 | Dowson 2019 | To determine the feasibility of implementing nurse-initiated polymerase chain reaction testing of respiratory specimens and to compare antibiotic prescribing prior to and during the implementation | Nurse education and standing orders with General Practitioners to facilitate respiratory specimen testing in suspected respiratory tract infection | N/A |
| 104 | Dräger 2017 | To test an intervention to optimize pain management and evaluate its effects on pain intensity and pain interference with function | Complex intervention training nursing staff and general practitioners on a new interdisciplinary guidance tool for pain assessment and management | Control group were invited to attend a 45-minute information event, which provided an introduction to the topic of pain, pain prevalence, chronic pain and physiological characteristics of pain in older adults |
| 105 | Drotningsvik 2019 | To assess the feasibility of conducting a nutritional intervention study in residents to investigate the effects of fish protein supplementation on markers of glucose metabolism and inflammation | Fish protein supplement (5.2 g of blue whiting protein hydrolysate) | Placebo (soft drink) |
| 106 | Dugre 2021 | To investigate the impact of medication regimen simplification on medication incidents at 12-month follow-up | A clinical pharmacist applied the validated 5-step Medication Regimen Simplification Guide for Residential Aged CarE | Usual care |
| 107 | Duru Aşiret 2018 | To assess the effect of individual reminiscence therapy on sleep quality | Reminiscence therapy once a week for 12 weeks in the form of individual sessions lasting 25–30 minutes | Usual care |
| 108 | Düzgün 2017 | To determine the effect of light therapy on sleep problems and sleep quality | Exposure to natural, bright sunlight between 8-10 AM after breakfast for five days | Usual care |
| 109 | El Alili 2020 | To assess the societal cost-effectiveness of Namaste Care Family programme in comparison with usual care in residents with advanced dementia | Namaste Care is a multidimensional care programme with psychosocial, sensory and spiritual components that incorporates tailored and personalized care until death for people with advanced dementia | Usual care |
| 110 | El Haddad 2020 | To examine the effect of an intervention comprising professional support by a geriatrician engaged in a quality care indicators’ audit for nursing home staff on reducing polypharmacy | Audit and feedback intervention with quality report critically discussed during cooperative work meetings with a volunteer public hospital geriatrician from their health administration subarea and nursing home leadership staff | Received the audit and feedback intervention only |
| 111 | Ellis 2019 | To evaluate a pain management programme using nonpharmacological approaches | The Pain Management programme involved a physiotherapist implementing four sessions per week of treatments (massage therapy, TENS, exercises and stretching, or combinations of these) | N/A |
| 112 | Ercan-Sahin 2018 | To determine the effect of reminiscence therapy as a nursing intervention on the quality of life of residents | The 12-session, 60 minutes per week reminiscence therapy programme | N/A |
| 113 | Erdal 2018 | To assess the efficacy and safety of analgesic treatment for depression in residents with advanced dementia and clinically significant depressive symptoms | Active analgesic treatment (acetaminophen/buprenorphine) | Placebo |
| 114 | Ergin 2019 | To find out how music, in particular, Nihavend mode, affects psycho-spirituality | Listened to music for 30 minutes between 8pm-10pm | Usual care |
| 115 | Eriksson 2016 | To evaluate the effect of a communication partner training programme directed to enrolled nurses working with people with communication disorders, using an individualised approach | Supervised analysis of video recorded natural interaction in everyday nursing situations and the formulation of individual goals to change particular communicative strategies | N/A |
| 116 | Ersek 2016 | To evaluate the effectiveness of a pain management algorithm coupled with intense diffusion strategies as compared with pain education only, in decreasing surrogate- and self-reported pain among residents | Intensive training and support for the use of recommended pain assessment and management practices using algorithms including handbook and four classes plus additional resources | Four one-hour classes in pain education |
| 117 | Evrard 2020 | To evaluate appropriateness of use and to identify factors associated with Benzodiazepine Receptor Agonist use and deprescribing | The intervention encompassed (1) education and training of health care professionals, (2) local interdisciplinary meetings (LIMs), and (3) repeated interdisciplinary case conferences (Figure 1). The intervention was global and not focused on BZRAs or psychotropic drugs; a few examples of BZRA-related PIPs and instructions on BZRA deprescribing were provided during the training | Usual care |
| 118 | Farlow 2016 | To enhance the recognition and treatment of residents with Alzheimer’s disease or other dementias | Quality improvement continuing medical education activity to retrospectively assess their current clinical practice; select areas for improvement; implement interventions based on treatment guidelines and health care standards; and then re-evaluate their practices and procedures | N/A |
| 119 | Fjeld 2018 | To study if the effect sustained in a longer perspective when toothbrushes were used according to resident’s own preference | Electric toothbrush | Manual toothbrush |
| 120 | Folkerts 2018 | To examine the feasibility and potential effects of Cognitive Stimulation in Parkinson's Disease Dementia | Cognitive stimulation programme twice weekly for 60 minutes | Usual care |
| 121 | Forbat 2020 | To determine whether a model of care providing specialist palliative care in care homes, called Specialist Palliative Care Needs Rounds, could reduce length of stay in hospital | Direct support (clinical work with residents) and indirect support in the form of ‘Needs Rounds’ - including team discussion, case-based education and a checklist to guide practice | Usual care |
| 122 | Forster 2021 | To develop and preliminarily test strategies to enhance physical activity in the daily life routines of residents to improve their physical, psychological and social well-being | MoveMore: a whole home intervention involving all care home staff designed to encourage and support increase in movement of residents | Usual care |
| 123 | Frändin 2016 | To evaluate the long-term effects on ADL, balance function, physical activity level, physical performance, falls-related self-efficacy, well-being as well as cognitive function | Individually tailored physical and daily activities in different combinations depending on the goals and physical and cognitive function of each participant | Usual care |
| 124 | Froggatt 2020 | To establish the feasibility and acceptability to staff and family of conducting a cluster randomised controlled trial of the Namaste Care intervention for people with advanced dementia | Group intervention to improve quality of life for people with advanced dementia | Usual care |
| 125 | Gajewska 2022 | To assess the impacts of a dietary intervention on the nutritional status, clinical outcomes and selected nutrient and salicylate intakes among residents | Personalised 3-month dietary intervention including one serving of supplementary food | N/A |
| 126 | Galik 2021 | To test the effectiveness of the Function and Behaviour Focused Care for the Cognitively Impaired intervention on function, physical activity and behavioural symptoms among residents with dementia | Function and Behaviour Focused Care for the Cognitively Impaired Intervention including assessment of policies and the environment, education and training, resident goal setting, ongoing training and motivation of staff | Educational control: Function and Behaviour Focused Care Education |
| 127 | Garland 2021 | To assess a comprehensive, person-centred advance care planning approach | BABEL ACP intervention comprises: (i)orientation and training for all nursing home staff, (ii)a workbook to guide advance care planning discussions (online materials), (iii)training tools for its use and (iv)stakeholder knowledge tools | Usual care |
| 128 | Garland 2022 | To assess the impact of a new pharmaceutical care model on polypharmacy and potentially inappropriate medication use | Care homes were exposed to the PEPS model (French acronym for Project of assessment of personalized pharmaceutical care in long-term care facilities) | Usual care |
| 129 | Gattinger 2017 | To investigate the use of a mobility monitoring system accompanied with case conferences to improve sleep quality in residents with cognitive impairment | Mobility monitoring system to enhance sleep quality - used intensively then reduced with case conferences led by registered nurses | Usual care |
| 130 | Gemelli 2016 | To determine if pharmacists’ intervention making a recommendation to the prescriber could have a positive impact on decreasing use of inappropriate sedative/hypnotics | Pharmacists making recommendations to the prescriber around reducing medications for insomnia | N/A |
| 131 | Gencbas 2018 | To assess the effectiveness of the nursing care plan implemented using NNN (NANDA-I, NOC and NIC) linkages for elderly women with incontinence | Use of NNN (NANDA-I, NOC and NIC) linkages in nursing care plans to provide holistic nursing care for people with incontinence | Usual care |
| 132 | Gillis 2016 | To evaluate a new way for applying bed baths and reducing the risk for dry skin by comparing the effect of two washing methods on skin hydration | Use of disposable wash gloves to undertake bed baths | Usual care |
| 133 | Gillis 2019 | To test a non‐pharmacological intervention, applied by the entire team and based on the resident's underlying needs, to reduce agitation and aggression | Either a) therapeutic touch b) group music sessions or c) meaningful individual activity. Using the ABC method and Senses Framework | N/A |
| 134 | Gine-Garriga 2020 | To assess the feasibility, acceptability, safety, and preliminary effects of an intervention to reduce sedentary behaviour | Get Ready intervention delivered by a staff champion one-to-one with resident and a family member | Usual care |
| 135 | Gök Ugur 2017 | To explore the effect of music therapy on depression and physiological parameters of residents | Music therapy intervention | Usual care |
| 136 | Gordon 2016 | To determine the impact of the ECHO-AGE intervention on the quality of care delivered to residents with dementia | Bi-weekly online video case discussions and didactic sessions on managing dementia and behaviour disorders | Usual care |
| 137 | Goyal 2021 | To examine the effects of the Sonas programme on anxiety and depression in residents with dementia | Multisensory stimulation in the Sonas program | Reading group: Reading only with no other activities in the Sonas program. Control group: continued with their routine activities provided by the home |
| 138 | Graham 2020 | To assess the feasibility of undertaking a definitive evaluation of a posture and mobility training programme for care staff | Skillful care training package delivered by physiotherapists to care staff | Usual care |
| 139 | Gravenstein 2017 | To compare the effect of the more immunogenic high-dose trivalent influenza vaccine with a standard-dose vaccine to identify the effect on reducing hospital admissions | High-dose influenza vaccination | Standard-dose influenza vaccination |
| 140 | Gravenstein 2021 | To compare the effect of adjuvanted trivalent influenza vaccine versus trivalent influenza vaccine on facility-reported influenza outbreaks | Adjuvanted trivalent influenza vaccine | Trivalent influenza vaccine |
| 141 | Guion 2018 | To determine whether an intervention based on education and professional support to staff would decrease the number of residents with a pain complaint, and to determine whether the intervention would improve pain management | Strong intervention group consisting of audit, feedback and collaborative work on quality indicators with a hospital geriatrician | Light intervention group consisting of audit and feedback |
| 142 | Gulla 2018 | To investigate the effect of medication review on antihypertensive drug use and the association between cognition, blood pressure, and prescribing | Systematic medication review where the physician received support from peers | Usual care |
| 143 | Gunst 2022 | To evaluate the effect of playing exergames on general wellbeing, fun and on social interaction | The intervention group played exergames twice a week | Usual care |
| 144 | Gustavsson 2015 | To evaluate the effect of impact absorbing flooring on fall-related injury risk | Impact absorbing flooring | Vinyl, linoleum or ceramic tiles |
| 145 | Guzmán 2016 | To understand the effect of this approach on the mood and behaviour of individual people living with mild to moderate dementia | A Psychomotor DANCe Therapy INtervention using Latin Ballroom (Danzón) 30 minute sessions twice weekly | N/A |
| 146 | Haddad 2018 | To determine the evolution of Behavioural and Psychological Symptoms in Dementia in residents after an environmental change through a relocation to a more architecturally suitable facility, while conserving the same medical staff | Relocation to a more architecturally suitable facility | N/A |
| 147 | Hahnel 2017 | To investigate the effectiveness of two structured skin care regimens in comparison to routine skin care on xerosis cutis in residents | A structured skin care regimen consisting of a moisturizing body wash and a moisturizing leave-on product (‘body lotion’) applied daily. Products of group I were a moisturizing body wash containing Shea Butter and glycerine and a hydrophilic water-in-oil emulsion. Group II received a glycerine containing body wash and a water-inoil emulsion containing emollients and 4% urea | Usual care |
| 148 | Haines 2020 | To evaluate whether an alternative model of care in aged care facilities, including in-house general practitioners, influenced health outcomes for residents | In the intervention model of care, GPs were members of staff at residential aged care facilities, care staff roles were redefined to allow registered nurses greater involvement in care plan development | Usual care |
| 149 | Halek 2020 | To describe the effects of two dementia-specific case conference models on the prevalence of behaviour that challenges | The intervention components were training and CC (Case conferences) sessions. Both CC models started with in-service training (6 h) in the respective CC model for core care team members followed by four 3-h supported CCs and four CCs without any external support | The homes were moved from traditional care to case conference sessions gradualy |
| 150 | Hanlon 2021 | To determine the impact of an educational quality improvement initiative on the appropriateness of antibiotic prescribing restricted to uncomplicated cystitis in older non-catheterized residents | Low-intensity multifaceted antimicrobial stewardship intervention | Usual care |
| 151 | Hansen 2018 | To gain new knowledge about how different décor and dinner plate colours can positively influence residents with dementia's level of food intake and appetite | Dinner plates with different colour combinations | N/A |
| 152 | Harrison 2021 | To compare the effects on agitation in people with Alzheimer's Disease or relation dementia | Music-basic intervention | Audiobooks used as control |
| 153 | Hartshorn 2021 | To determine if the number of certified nursing assistants trained with the Mouth Care Matters oral health educational programme had an impact on oral health | Two intervention groups: All certified nursing assistants were offered the Mouth Care Matters (MCM) programme; Three certified nursing assistants were offered the MCM training to simulate an oral health care champion model | Usual care |
| 154 | Hashimoto 2017 | To examine the effects of the administration of docosahexaenoic acid-enriched meals on cognitive function in the oldest elderly with cognitive impairment, such as dementia | Family-style meals containing an additional 1720 mg of docosahexaenoic acid per day | Placebo |
| 155 | Hassan 2016 | To pilot an investigation into the impact of resistance training on sarcopenia status in residents | Twice weekly progressive resistance training | Usual care |
| 156 | Henskens 2018a & 2018b | To evaluate the effects of movement stimulation on activities of daily living performance and quality of life in residents with dementia | Three interventions: Activities of Daily Living (ADL) training, Multicomponent aerobic and strength exercise training, Combination of both | Usual care |
| 157 | Henwood 2015 | To assess the feasibility of a dementia-specific aquatic exercise programme for residents with a particular focus on the physical and functional benefits | Twice weekly aquatic exercise – 45 minutes approximately | N/A |
| 158 | Hermans 2018 | To evaluate whether using the interRAI Palliative Care instrument is associated with reduced needs and symptoms in residents nearing the end of their lives | Introduction of the interRAI Palliative Care Instrument with staff training followed by introduction into routine care | Usual care |
| 159 | Hewitt 2018 | To determine if combined high level balance and moderate intensity progressive resistance training is effective in reducing the rate of falls | The Sunbeam programme consisted of individually prescribed progressive resistance training plus balance exercise performed in a group setting | Usual care |
| 160 | Hirdes 2020 | To evaluate the impact of a multicentre intervention to reduce potentially inappropriate antipsychotic use at the individual and facility levels | Quality improvement teams in participating organizations were provided with education, training, and support to implement localized strategies intended to reduce antipsychotic medication use in residents without diagnosis of psychosis | Usual care |
| 161 | Hjetland 2021 | To assess whether Increasing daytime light exposure using bright light treatment may represent a feasible non-pharmacological treatment for sleep problems in residents with dementia | A LED ceiling-mounted bright light solution, delivered by Glamox AS (supplier of professional lighting solutions), was installed in the common rooms of the four intervention units | Control condition was created by replacing the light bulbs in the existing fittings with conventional 3000 K light bulbs in all common rooms |
| 162 | Hmwe 2022 | To evaluate the outcomes of an acupressure intervention to improve sleep quality and wellbeing | Acupressure and routine care | Usual care |
| 163 | Hockley 2016 | To implement the Gold Standards Framework in Care Homes Programme and audit outcomes within homes across five Clinical Commissioning Groups over a 7–year period using a research-based model of facilitation | High facilitation roll-out of the Gold Standards Framework for Care Homes, advance care planning and use of end-of-life care pathways | N/A |
| 164 | Hodl 2019 | To measure the effectiveness of 29 evidence-based nursing recommendations regarding the conservative management of urinary incontinence | Implementation of 29 evidence-based recommendations for the conservative management of urinary incontinence among female residents | Usual care |
| 165 | Hollaar 2017 | To examine if the daily application of 0.05% chlorhexidine oral rinse solution is effective in reducing the incidence of aspiration pneumonia in residents with dysphagia | Usual oral hygiene care with the addition of a 0.05% chlorhexidine oral rinse solution | Usual care |
| 166 | Hopkins 2017 | To investigate the effect of increasing indoor light levels with blue-enriched white lighting on objective (rest-activity rhythms, performance) and self-reported (mood, sleep, alertness) measures | Blue-enriched white lighting in communal areas | White lighting in communal areas |
| 167 | Hopper 2016 | To explore the relationship between hearing loss and cognitive-communication performance of individuals with dementia, and to determine if hearing loss is accurately identified by staff | Cognitive communication testing with amplification | Cross-over design |
| 168 | Huey-Ming 2021 | To assess if the personal impact of falls can be prevented by implementing injury prevention actions tailored to individual resident needs and engaging residents and families in fall prevention care | Fall Tailoring Interventions for Patient Safety programme in which nursing staff can modify fall prevention interventions based on daily assessments supported by programme toolkit | N/A |
| 169 | Hullick 2016 & 2022 | To determine if adding video-telehealth consultation to the established Aged Care Emergency programme further reduced Emergency Department visits and hospital admissions | Clinical support to nurses, allowing residents to be managed in place to avoid transfer to the ED | Usual care |
| 170 | Hurley 2020 | To evaluate whether Active Residents in Care Homes programme can be delivered, its effects on resident’s physical activity, wellbeing and costs | Occupational and physiotherapists implemented Active Residents in Homes programme designed to increase physical activity by facilitating whole-system change | N/A |
| 171 | Husebø 2019 & Gedde 2021 & Habiger 2021 | To investigate if the multicomponent intervention of the COSMOS trial (Communication, Systematic pain management, Medication reviews, Organization of activities, and Safety) improved quality of life in residents with complex needs | COSMOS: communication, systematic pain management, medication reviews, activities, and safety multicomponent intervention | Usual care |
| 172 | Inch 2019 | To test and refine the service specification and proposed study processes to inform the design and outcome measures of a definitive randomised controlled trial to examine the clinical and cost effectiveness of Pharmacist Independent Prescribers working in care homes compared to usual care. | Independent pharmacist prescribing on falls, medications, resident’s quality of life and activities of daily living, mental state and adverse events | N/A |
| 173 | Isaac & Parajuli 2021 | To evaluate the outcomes of a person- centred, non-pharmacological dementia care model, ‘Harmony in the Bush’, based on the Progressively Lowered Stress Threshold principles and person-centred music | Harmony in the Bush is a person-centred model of dementia care in nursing homes, based on the principles of Progressively Lowered Stress Threshold and person-centred music intervention. A person-centred care plan and personalized preferred music playlists were provided to participants | N/A |
| 174 | Iuliano 2021 | To assess the anti-fracture efficacy and safety of a nutritional intervention in older adults replete in vitamin D but with mean intakes of 600 mg/day calcium and <1 g/kg body weight protein/ day | Additional milk, yoghurt, and cheese that contained 562 (166) mg/day calcium and 12 (6) g/day protein achieving a total intake of 1142 (353) mg calcium/day and 69 (15) g/day protein (1.1 g/kg body weight) | Usual care |
| 175 | Jablonski 2018 | To test the efficacy of MOUTH (Managing Oral Hygiene Using Threat Reduction), a nonpharmacologic, relationship-based intervention | An evidence-based mouth-care protocol for older adults with natural dentition and dentures, recognition of care resistive behaviours and strategies to reduce threat perception during the provision of mouth care | Usual care |
| 176 | Jahanpeyma 2021 | To evaluate the effects of Otago exercises on falls, balance, and physical performance in older adults with high falls risk | Performed 45 minutes of Otago exercises 3 days/week for 12 weeks plus a walking programme on 3 other days of the week | Walking programme (minimum 30 minutes, 3 days per week) |
| 177 | Jansen 2018 | To determine whether a multicomponent, individually tailored intervention to promote physical activity enhances life-space utilization in residents and whether intervention effects can be sustained at follow-up after continuation of the programme as part of institutional daily routines | Multicomponent exercise: group sessions, individual exercise and serious games training tailored to residents’ individual functional capacity | Usual care |
| 178 | Jarboe 2015 | To evaluate the impact of a quality assurance performance improvement initiative implementing the INTERACT™ (interventions to reduce acute care transfers) SBAR (situation, background, assessment/appearance, recommendation) communication tool | INTERACT™ (interventions to reduce acute care transfers) SBAR (situation, background, assessment/appearance, recommendation) communication tool | N/A |
| 179 | Jeong 2022 | To develop and evaluate a multifaceted and evidence-based delirium educational programme for practitioners | Staff received the developed delirium educational programme for 3 weeks | Staff received a delirium handbook |
| 180 | Johansson 2020 | To improve oral health care‐related beliefs of nursing staff and the oral health of residents | An oral health coaching programme where two dental hygienists, employed at the Swedish Public Dental Service, were available four hours/week for three months to support the nursing staff in performing oral care to the residents | Usual care |
| 181 | Johnen 2018 | To examine the feasibility of a 3-month machine vs. free weight strength training programme in residents, and to determine the effects on physical performance | Free weight training twice per week (45-60 minutes) | Machine training twice per week (45-60 minutes) |
| 182 | Jøranson 2021 | To investigate effects from robot-assisted (Paro) group activity on sleep patterns in residents with dementia | Group activity with Paro (robotic seal) 30 minutes twice a week | Usual care |
| 183 | Jordan 2015 | To assess the number and nature of clinical problems identified and addressed and changes in prescribing following introduction of nurse-led medicines’ monitoring | Nurses completed the West Wales ADR Profile for Mental Health Medicines with each participant | N/A |
| 184 | Jordan 2017 | To identify the initial effect of a coaching training intervention using Level of Assistance (LoA) strategies compared with traditional lecture techniques on the appropriateness of LoA use by certified nursing assistants' and independence of dressing of residents with dementia | The coaching intervention involved three face-to-face sessions including teaching, an examination, role play, goal setting and feedback on recorded care delivered | Teaching session on level of assistance training followed by a short examination |
| 185 | Jung 2018 | To examine the effects of a client-centred leisure activity programme on satisfaction, upper limb function, self-esteem, and depression | Client-centred leisure activity program, three times per week, 30 minutes per session | Existing leisure activities provided by the nursing facility |
| 186 | Junius-Walker 2021 | To improve drug safety in residents, including a reduction of potentially inappropriate medications and/or neuroleptic use, by means of a complex interprofessional intervention | A drug review by trained pharmacists, educational sessions for general practitioners and nurses, a drug safety toolbox, and change management seminars for members of the three participating professions | Usual care |
| 187 | Jupiter 2016 | To evaluate the benefits obtained when residents were trained and fitted with Hearing Assistive Technology | Each individual was fitted with the Hearing Assistive Technology and trained to use it during an initial half-hour session | N/A |
| 188 | Juthani-Mehta 2016 | To test the effect of two oral cranberry capsules once a day on presence of bacteriuria plus pyuria among female residents | Two cranberry capsules | Placebo |
| 189 | Kalinowski 2015 | To analyse the impact of being pain-affected and fear of falling on functional performance | Extensive pain management training and guidance for staff | Staff given access to online course and one 45-minute event about general pain management |
| 190 | Kalinowski 2019 | To assess the non-pharmacologic pain management therapies (NPTs) provided and to enhance the application and prescription of NPTs in residents on an individual level | Education programme - online course for physicians and one-day seminar for nurses | Basic information on pain management |
| 191 | Kalu 2021 | To examine the effects of a walking intervention on gait velocity, and stride length, cadence and heel-to-heel base of support compared to those in an interpersonal interaction control group and a care-as-usual control group | Walking group 1:1 supervised, individualized, progressive, 30 minutes, five times a week | Two control groups: interpersonal interaction and usual care |
| 192 | Kane 2017 | To determine whether training and support for implementation of a quality improvement programme (Interventions to Reduce Acute Care Transfers [INTERACT]) reduced hospital admissions and emergency department visits | Training and support for implementing INTERACT, which included tools that help staff identify and evaluate acute changes in resident condition and document communication between physicians; care paths to avoid hospitalization when safe and feasible; and advance care planning and quality improvement tools. | Usual care |
| 193 | Kårefjärd 2019 | To investigate the effects of dog-assisted intervention on quality of life in residents with dementia | Dog-assisted intervention tailored to the resident prescription | N/A |
| 194 | Keating 2020 | To evaluate the effectiveness of group reminiscence arts sessions for people living with dementia | Received the Reminiscence, Arts and Dementia: Impact on Quality of Life intervention | Usual care |
| 195 | Kennedy 2015 | To examine the effectiveness of a multifaceted, interdisciplinary Knowledge Translation intervention for improving the prescribing of vitamin D, calcium and osteoporosis medications over 12-months | Multifaceted intervention including educational meetings, quality improvement work, audit and feedback | Usual care |
| 196 | Kim 2016 | To examine the long-term effects of foot-bathing therapy, using different water temperatures, on the sleep quality of residents | Two intervention groups: intervention group had footbath therapy with water temperature at 40 degrees Celsius, 'placebo group' had footbath therapy with water temperature at 36.5 degrees | Usual care |
| 197 | Kim 2021a | To evaluate the impact of an information and communication technologies-enhanced, multidisciplinary integrated care model, called Systems for Person-centred Elder Care | Systems for Person- centred Elder Care intervention guided by the chronic care model consists of 5 strategies: comprehensive geriatric assessment, care planning, optional interdisciplinary case conferences, care coordination, and a cloud-based ICT tool along with a free messaging application | Usual care |
| 198 | Kim 2021b | To determine the effect of a group music intervention with rhythmic exercises programme on cognitive function and mental health outcomes | Bi-weekly group music intervention with rhythmic exercises programme and a regular activities program | Received only a regular activities programme provided by the home |
| 199 | Kirkham 2019 | To evaluate a multicomponent approach to reduce inappropriate prescribing of antipsychotics | Optimising Prescribing of Antipsychotics in Long-term care programme consisted of an educational in-service provision of evidence-based tools to assess and monitor neuropsychiatric symptoms and monthly interdisciplinary team meetings to discuss individuals for whom an antipsychotic could be reduced or discontinued | N/A |
| 200 | Klotz 2018 | To evaluate the association between oral health and 1-year mortality among residents with or without oral health intervention | Oral health programme was implemented including dental education, instruction in standardized assessment of oral health for non-dentists, and introduction of ultrasonic baths for denture cleaning | Usual care |
| 201 | Kohler 2018 | To explore the effects of an educational programme and subsequent nursing case conferences on urinary incontinence and quality of life in residents with dementia and incontinence | Educational programme and case conferences for nursing teams | N/A |
| 202 | Kok 2018 | To examine the possible beneficial effects on quality of life of older people with moderate to severe dementia who moved from a large special care unit (SCU) to a small scaled homelike SCU | Move to small scaled special care unit facility with 7-8 residents. The entire nursing staff of the intervention group received a training focused on the new accommodation. | Remained in their largescale living environment and received care as usual with no special training for the personnel |
| 203 | Könner 2015 | To evaluate the effect of interventions for general practitioners and nursing home staff to improve pain severity and appropriateness of pain medication in residents | Two multifaceted education programs were developed by the interdisciplinary study team for the residents’ GPs and nursing home staff | Nursing home staff in each control group facility were offered a 45-minute presentation about general pain management |
| 204 | Kovach 2019 | To describe the use of warmed blankets and determine if use was associated with changes in pain, agitation, mood, or analgesic use | Warmed blankets were unfolded and placed over residents with pain, agitation or thermal discomfort | N/A |
| 205 | Kurt 2021 | To determine the effect of music therapy on the feeling of loneliness | Listened to instrumental music for 30 minutes in the Rast maqam with naked ear, in the sitting position, for 10 days, both in the morning and evening being 20 times in total, in the activity room of the nursing home | Usual care |
| 206 | Kuru 2017 | To evaluate the effect of Laughter therapy on the quality of life of residents | Laughter therapy was applied with nursing home residents of the experimental group two days per week (21 sessions in total) | Usual care |
| 207 | Kuru Alıcı 2018 | To evaluate the preliminary effect of laughter therapy on the level of loneliness and death anxiety | Laughter therapy twice a week for 5 weeks | Usual care |
| 208 | Kushiro 2019 | To examine the efficacy of fermented milk consumption in suppressing fever, alleviating constipation, improving peripheral blood and salivary secretory IgA parameters, and modulating faecal microbiota | Drank one bottle of fermented milk containing Lactobacillus casei strain Shirota daily | Placebo |
| 209 | Kütmeç Yilmaz 2021 | To examine the effect of progressive muscle relaxation exercises on adaptation to old age and the quality of life of older people | Progressive muscle-relaxation sessions twice weekly for 8 weeks | Usual care |
| 210 | Kutschar 2020 | To improve the pain situation of residents following a nursing-related educational intervention within a cluster-randomized controlled trial | Educational intervention in pain management, containing classroom (quality circles) and web-based training for nurses. | Usual care |
| 211 | Lamppu 2021 | To examine the effects of end-of-life training on residents’ health-related quality of life and use and costs of hospital services | Small-group 4-hour educational sessions on the principles of palliative and end-of-life care (advance care planning, adverse effects of hospitalizations, symptom management, communication, supporting proxies, challenging situations) provided to all members of staff | Usual care |
| 212 | Laurence 2019 | To investigate the effect of a checklist for oral care supplemented by random inspections by a charge nurse on improving oral hygiene | Daily use of a checklist for oral care by nursing staff supplemented by random inspections by a charge nurse | Usual care |
| 213 | Lavallée 2019 | To assess the feasibility of implementing a pressure ulcer prevention care bundle | Theory and evidence‐informed care bundle specifically for nursing homes, which consists of three prevention practices (skin inspection, support surfaces, repositioning) and a range of behaviour change techniques to promote these practices | N/A |
| 214 | Lavigne 2017 & 2018 | To investigate whether twice-daily use of a rotating–oscillating power toothbrush in residents would reduce systemic inflammation as measured by C-reactive protein levels | Twice-daily use of a rotating–oscillating power toothbrush (Oral-B Professional Care 1000™) | Usual care |
| 215 | Lee 2017 | To examine the effects of lactose-free milk intake and whole-body vibration exercises on bone density in residents who had difficulty exercising outdoors and did not regularly consume milk | Participants received lactose-free milk (190ml) per day and received whole body vibration exercise using a whole-body vibrator | Usual care |
| 216 | Lee 2022 | To examine the effects of auricular acupressure on sleep and pain in residents with osteoarthritis | Acupressure applied to selected auricular acupoints related to sleep improvement and pain reduction | Tapes applied to points which are irrelevant to sleep and joint pain |
| 217 | Leguelinel-Blache 2020 | To assess assessed the impact of multidisciplinary medication review on resident safety and cost | Prescriptions reviewed by pharmacist with modifications made to the patient’s medical team | N/A |
| 218 | Lexow 2022 | To examine the impact of a one-time, pharmacist-led medication review on medication changes | Structured medication review undertaken by a pharmacist | Usual care |
| 219 | Liao 2020 | To evaluate the impact of an antibiotic stewardship intervention that promotes the use of an Antibiotic time-out on antibiotic changes in nursing homes | Optimizing Antibiotic Stewardship in Skilled Nursing Facilities programme using antibiotic time-outs on antibiotic prescriptions | Usual care |
| 220 | Linander 2020 | To study the effect of indoor circadian- adjusted LED-lighting on sleeping patterns and systemic bio- markers associated with inflammation | Circadian- adjusted LED-lighting was installed on one floor of the care home | Static white lights installed on another floor of the care home |
| 221 | Little 2019 | To introduce an early warning tool, the Significant 7, to facilitate identification and management of deterioration in residents | Significant 7 early warning tool (seven signs of deterioration: confusion, mood, pain, hydration, skin, breathing and toilet or bowel habits) including guidance on how to manage these signs | N/A |
| 222 | Liu 2020 | To examine the impact of providing specialist palliative care on residents’ quality of death and dying | Palliative Care Needs Rounds are monthly hour-long staff-only triage meetings to discuss residents at risk of dying without a plan in place. They are chaired by a specialist palliative care clinician and attended by care home staff. A checklist is followed to guide discussions and outcomes, focused on anticipatory planning | Usual care |
| 223 | Livingston 2019 | To assess whether the Managing Agitation and Raising Quality of Life intervention reduced agitation in residents with dementia after 8 months compared with treatment as usual | Evidence-based Managing Agitation and Raising Quality of Life manualised intervention delivered by psychologists to care home staff in six sessions | Usual care |
| 224 | Logan 2021 | To determine the clinical and cost effectiveness of a multifactorial fall prevention programme | Guide to Action for Care Homes: a multifactorial fall prevention programme that involved awareness raising, education, screening, decision support, and implementation support | Usual care |
| 225 | Lok 2017 | To determine how a “Physical Activity Program” affected depressive symptoms and quality of life | The ten-week “Physical Activity Program” included 10-minute of warm-up activities as the initial segment, 20-minute of rhythmic exercises as the activity segment,10-minute of cool down exercises as the final segment and 30-minute of free walking time | Usual care |
| 226 | Low 2015 | To evaluate outcomes of Grandfriends, an intergenerational programme for people living in nursing homes as a result of their dementia symptoms and children attending a preschool co-located within the facility precinct | Grandfriends programme– interactions with preschoolers | Usual care |
| 227 | Low 2016 | To evaluate the feasibility of a dance programme for people with moderate to severe dementia with regards to recruitment and retention, assessment tools, intervention safety, attendance and engagement | Dance sessions thrice weekly for 45 minutes | Music appreciation and socialisation groups, thrice weekly for 45 minutes |
| 228 | Machacova 2017 | To determine whether dance-based intervention could reverse functional decline among residents | Weekly 1 hour dance-based exercise programme | Usual care |
| 229 | Mackey 2019 | To assess the clinical effectiveness of compliant flooring at preventing serious fall-related injuries | Compliant flooring (2.45cm SmartCells) | Rigid control flooring |
| 230 | Madden 2015 | To compare antibiotic susceptibility and proportions of non–E. coli *Enterobacteriaceae* among Gram-negative urinary isolates from participants randomized to cranberry capsules compared to placebo | Daily doses of 0, 1, 2, and 3 capsules containing 0, 36 mg, 72 mg, and 108 mg of proanthocyanidin respectively | Placebo |
| 231 | Mahlknecht 2019 | To improve interprofessional communication and medication safety using a combined intervention and thus, to improve medication appropriateness and health-related outcomes of the included residents | Systematic education of participating healthcare professionals plus structured interprofessional medication review | N/A |
| 232 | Maidment 2018 | To determine whether it is feasible to implement and measure the effectiveness of a dual-purpose pharmacy health psychology intervention incorporating medication review and staff training to limit the prescription of psychotropics to manage behavioural and psychological symptoms in dementia in residents | A specialist pharmacist reviewed medication. Care home staff received an educational behaviour change intervention in a three-hour session promoting person-centred care and primary healthcare staff received a modified version of the training | N/A |
| 233 | Mak 2022 | To undertake subgroup analysis of the Sunbeam trial, to determine whether the intervention was effective for reducing falls in residents with mild-moderate cognitive impairment/ dementia | Resistance and balance training programme | Usual care |
| 234 | Maltais 2018 & 2019a & 2019b | To evaluate the effect of exercise on residents with dementia | Structured multicomponent exercise (moderate), twice weekly for 60 minutes | Social activity (music, meditation arts and crafts) |
| 235 | Man 2020 | To assess the clinical and patient-centred effectiveness of a novel residential ocular care model | Residential ocular care a tailored and comprehensive within-site eye examination and care rehabilitation pathway | Usual care |
| 236 | Marmeleira 2018 | To examine the feasibility and the effects of a multimodal exercise intervention on the physical and cognitive functioning of residents | Twice weekly exercise programme | N/A |
| 237 | Martin 2019 | To determine whether Goals of Patient Care medical treatment orders were more effective than advance care planning alone in preventing emergency department (ED) visits (no hospitalization), ED visits (with hospitalization), and deaths outside the residential aged care facility | Completion of Goals of Patient Care process by a geriatrician, following a shared decision-making process, incorporating advance care planning documents or residents’ preferences | Usual care |
| 238 | Mbakile-Mahlanza 2020 | To evaluate the impact of the Montessori activities implemented by family members on visitation experiences with people who have dementia | During visits family members interacted with their relative through engaging in Montessori-based activities for four 30-minute sessions | During visits family members interacted with their relative by reading a newspaper for four 30-minute sessions |
| 239 | McCabe 2019 | To evaluate a consumer directed care training programme for staff | Staff training - six sessions focusing on communication skills, organizational change and transformational leadership in care homes Arm 1 training plus support; Arm 2 training only | Usual care |
| 240 | McCabe 2022 | To evaluate the six-session Resident at the Centre of Care staff training programme designed to equip staff to implement a Consumer-Directed Care model of care among residents | Staff training - six sessions focusing on communication skills, organizational change and transformational leadership in care homes Arm 1 training plus support; Arm 2 training only | Usual care |
| 241 | McConeghy 2021 | To test whether adjuvanted trivalent influenza vaccine is more immunogenic than similarly dosed nonadjuvanted trivalent influenza vaccine | Administered adjuvanted trivalent influenza vaccine | Adminstered non-adjuvanted trivalent influenza vaccine |
| 242 | McCord 2020 | To examine the change in executive functioning in healthy adults via video game intervention | Residents played Star Wars Battlefront for six supervised sessions of 30 minutes each | Usual care |
| 243 | McCreedy 2022 | To test effect of a personalized music intervention on agitated behaviours and medication use among residents with dementia | Personalised music played by nursing home staff to pre-empt or reduce agitation | Usual care |
| 244 | McDerby 2019 | To investigate the effect of a pharmacist on medication administration practices as a strategy for improving quality use of medicines | On-site clinical pharmacist | Usual care |
| 245 | McDermid 2022 | To evaluate a digital person-centred care training intervention | Digitally adapted version of the WHELD person-centred care home training programme with virtual coaching | The digital training programme alone |
| 246 | McGilton 2017 | To determine the effects of a communication intervention on residents’ quality of life and care, as well as care providers’ perceived knowledge, mood, and burden | Implementation of a resident centred communication intervention for people living with dementia | N/A |
| 247 | McMaughan 2016 | To test the effectiveness of a decision-making aid for urinary tract infection management on reducing antibiotic prescriptions for suspected bacteriuria in the urine without symptoms, known as asymptomatic bacteriuria | Two intervention groups: decision-making aid with high intensity training and decision-making aid with low intensity training | Usual care |
| 248 | Meeks 2015 | To report the primary outcomes of a cluster randomized clinical trial of Behavioural Activities Intervention, a behavioural intervention for depression | Weekly sessions with a mental health therapist | Usual care |
| 249 | Mestres 2015 | To establish a medication review programme in residents | Pharmacist review using an algorithm developed by the Pharmacy Service according to criteria of efficacy, safety, efficiency and standard in geriatrics | N/A |
| 250 | Middelstädt 2016 | To investigate the effects of cognitive stimulation on cognition, quality of life, behavioural symptoms, and activities of daily life in persons with dementia | Cognitive stimulation programme twice weekly | Usual care |
| 251 | Millett 2021 | To investigate effects of the Java Music Club, a manualized social support program, on cognition and psychosocial health among residents | Java Music Club was held for one hour, once a week, each session includes singing and discussion related to the theme of the week, selected by participants | N/A |
| 252 | Mitchell 2018 | To test whether an ACP video (vs usual care) has an effect on documented advance directives, level of care preferences, goals-of-care discussions, and burdensome treatments among residents with advanced dementia | A 12-minute advance care planning video for proxies with written communication of their preferred level of care (comfort, basic, or intensive) to the primary care team | Usual care |
| 253 | Mitchell 2020, Loomer 2021, Moyo 2022 | To test the effectiveness of a multicomponent intervention to improve the management of suspected urinary tract infections and lower respiratory infections for residents with advanced dementia | Nursing home practitioners given an in-person seminar, an online course, management algorithms (posters, pocket cards), communication tips (pocket cards), and feedback reports on prescribing of antimicrobials | Usual care |
| 254 | Mitchell 2021 | To evaluate the impact of an Advance Care Planning Video Intervention on Care of Short-Stay Nursing Home residents | Five 6- to 10-minute ACP videos offered 1) within 7 days of admission for short-stay or 2) every 6 months for long stay | Usual care |
| 255 | Mitolo 2017 | To assess the efficacy of a route-learning training | Detailed three-part training on attitudes to spatial navigation, routes reproduction and routes mental representation | Usual care |
| 256 | Miyazaki 2020 | To test whether a drumming communication programme would improve cognitive and physical function in residents with cognitive impairment | Drumming communication programme 30 minutes thrice a week | Usual care |
| 257 | Mody 2015 | To test whether a multimodal targeted infection programme reduces the prevalence of multidrug resistant organisms and incident device-related infections | Multimodal intervention, including pre-emptive barrier precautions, active surveillance for multidrug resistant organisms and infections, and nursing home staff education | Usual care |
| 258 | Mody 2021 | To determine whether a multicomponent infection prevention intervention can reduce multidrug resistant organism prevalence | Multi-component including enhanced barrier precautions, chlorhexidine bathing, multidrug resistant organism surveillance, environmental cleaning education and feedback, hand hygiene promotion, and health care worker education and feedback | Usual care |
| 259 | Moniz-Cook 2017 | To assess the effectiveness and cost-effectiveness of online training and decision support for staff to deliver functional analysis-based interventions for challenging behaviour in dementia | Online individualised intervention for case-specific action plans to reduce challenging behaviour in dementia consisting of e-learning and bespoke decision support care home and family e-tools | Usual care |
| 260 | Morales 2017 | To evaluate adherence to a resident-focused hand hygiene intervention based on the Health Belief Model | Hand hygiene intervention designed for residents including disposable wipes and structured education | N/A |
| 261 | Morgan 2020 | To evaluate whether contact precautions decreased MRSA (Methicillin-Resistant Staphylococcus aureus) acquisition | Contact precautions for infection prevention and control | Usual care |
| 262 | Mouton 2017 | To examine the effects of a giant exercising board game intervention on ambulatory physical activity and a broader array of physical and psychological outcomes among residents | Giant exercising board game that required participants to perform strength, flexibility, balance and endurance activities | Participants requested not to get involved in any new type of physical activity |
| 263 | Moyle 2019 | To compare a lifelike baby doll intervention for reducing anxiety, agitation, and aggression in older people with dementia, with usual facility care | Lifelike baby doll intervention consisting of three 30-minute individual non-facilitated sessions per week | Usual care |
| 264 | Mulasso 2015 | To assess the direct and indirect effects of a multicomponent exercise programme on mobility and balance | Range-of-motion, strength and balance exercises – 75 minutes, twice weekly | Usual care |
| 265 | Nace 2020 | To determine the association of a multifaceted antimicrobial intervention with the reduction in unnecessary antimicrobial use for unlikely cystitis among non-catheterised residents | Introductory webinar, pocket-sized educational cards, tools for system change, and educational clinical vignettes addressing the diagnosis and treatment of suspected uncomplicated cystitis | Usual care |
| 266 | Naczk 2020 | To evaluate the impact of inertial training on upper and lower extremity strength | Inertial training twice a week using a Cyklotren inertial device | Usual care |
| 267 | Nagata 2016 | To clarify the usefulness of *Lactobacillus casei* strain Shirota-fermented milk in the normalization of bowel movements and improvement of infection control | Lactobacillus casei strain Shirota-fermented milk daily | Placebo |
| 268 | Nagayama 2016 | To evaluate if interventions based on occupation-based goal setting using the Aid for Decision-making in Occupational Choice could focus on meaningful activities to improve quality of life and independent activities of daily living, with greater cost-effectiveness than an impairment-based approach as well as to evaluate the feasibility of conducting a large cluster, randomized controlled trial | Received occupational therapy based on occupation-based goal setting using the Aid for Decision-making in Occupation Choice, and the interventions were focused on meaningful occupations | Impairment-based occupational therapy approach focused on restoring capacities, without goal setting tools |
| 269 | Nam 2016 | To investigate the effects of using intra and extra circumoral exercise on subjective and objective parameters of pre- and post-oral health of residents | Intra and extra circumoral exercise, 25 min a day, six times per week for 4 weeks | Usual care |
| 270 | Namasivayam-MacDonald 2017 | To determine the feasibility and effectiveness of an 8-week tongue strengthening intervention protocol for seniors with mild to moderately severe cognitive impairment | Therapy sessions in which participants performed isometric strength exercises and tongue pressure accuracy tasks using the Iowa Oral Performance Instrument | N/A |
| 271 | Nawrat-Szoltysik 2018 & 2019 | To evaluate the effect of a programme of modified Sinaki exercises and Nordic Walking in osteoporotic and osteopenic females | Three intervention groups received pharmacological treatment for osteoporosis plus: Sinaki exercises, Nordic walking, Sinaki & Nordic walking | Pharmacological treatment for osteoporosis |
| 272 | Nishiura 2018 | To investigate the effects of a new technological intervention—a parametric speaker, creating a narrow personal acoustic environment, which may reduce the manifestation of behavioural and psychological symptoms of dementia symptoms | Participants sat in the communal areas of the home and listened to their favourite pieces of music, as selected by their families | N/A |
| 273 | Obayashi 2020 | To examine the impact of age, gender and dementia stage on an assistive technology intervention using communication robots | Participants given two robots: Robot A programmed to start talking to participants at certain times and Robot B who interacted during recreation | Usual care |
| 274 | Okan 2022 | To determine the effect of sunlight exposure to reach optimum 25- hydroxyvitamin D [25(OH)D] levels | Exposed to sunlight five days a week (max: 30 minutes) | No sunbathing |
| 275 | Okkels 2021 | To increase health-related quality of life in residents receiving meals-on-wheels using an intervention menu with a culinary twist | Intervention menu of popular meals-on-wheels with culinary improvements | The same meals without culinary improvements |
| 276 | Olsen 2016 | To examine the possible effects on depression, agitation and quality of life in residents with dementia or cognitive impairment, through an intervention with animal assisted activities and a follow-up study | Animal Assisted Activities twice weekly lasting 30 minutes | Usual care |
| 277 | Onieva-Zafra 2018 | To investigate the effect of an 8-week nursing intervention consisting of 2 weekly sessions of music and reminiscence therapy together with the application of reality orientation techniques | Music programme intervention using different musical experiences including listening and singing accompanied by reminiscence therapy and reality orientation | Usual care |
| 278 | O'Sullivan 2022 | To investigate global and momentary effects of a tablet-based non-pharmacological intervention for residents with dementia | Regular tablet-based intervention with stimulating activities developed to engage people with dementia | Usual care |
| 279 | Overgaard 2022 | To compare a designated shared oral care intervention with a standard programme, focusing on levels of plaque and inflammation | Intervention adjusted individually to the specific social interaction between the resident, nursing staff and dental staff | Usual care |
| 280 | Papadopoulos 2022 | To evaluate a culturally competent artificial intelligent system embedded into social robots to support older adult wellbeing | Pepper robot for up 18 h across 2 weeks. Two versions of the CARESSES artificial intelligence were tested: a fully culturally competent system (Experimental Group) and a more limited version (Control Group 1) | Usual care |
| 281 | Park 2015 | To examine the effect of the Evidence-Based Nursing Care Algorithm of Dysphagia (ENCAD) on risk of dysphagia, oral health, and dysphagia-specific quality of life among residents | Evidence Based Nursing Care Algorithm of Dysphagia | N/A |
| 282 | Park 2019 | To compare the effects of hand massage with the preferred aroma oil to those of hand massage with lavender oil on stress and sleep in residents | Hand massage with preferred aroma oil | Hand massage with lavender oil |
| 283 | Parsons 2017 & Elia 2018 | To compare the effects of two common forms of nutritional support, Dietary Advice and Oral Nutritional Supplements, on quality of life and other outcomes including weight, nutritional intake and appetite | Oral nutritional supplements | Dietary advice |
| 284 | Pasay 2019 | To measure the impact of an antimicrobial stewardship initiative on the rate of urine culture testing and antimicrobial prescribing for urinary tract infections | On-site staff education, physician academic detailing, and integrated clinical decision- making tools | Usual care |
| 285 | Pasina 2016 | To optimize the prescription of psychotropic drugs, according to the Beers recommendations, through a multicomponent intervention, education of general practitioners, and the use of INTERcheck | Three educational interventions (‘ex cathedra’ presentations) were organized by the researchers involved in the project, and a fourth training session was also held on the use of INTERCheck, a Computerized Prescription Support System (CPSS) developed to optimize drug prescription for older people with multimorbidity. | N/A |
| 286 | Pasquel 2015 | To compare the efficacy and safety of treatment with basal insulin, and oral antidiabetic drug therapy regimen in residents with type 2 diabetes | Basal insulin regime - titrated based on blood sugars | Oral antidiabetic therapy, continued unless contraindications or blood glucose >200mg/dL resulting in commencement of sliding-scale |
| 287 | Passmore 2018 | To evaluate use of resistant band exercise programmes and reported life-satisfaction | Chair resistance band exercise programme, three days per week | Usual care |
| 288 | Pedersen 2018 | To investigate the effect on readmissions of a follow-up visit in a population of residents admitted to an Emergency Department with one of nine medical diagnoses | Individualised follow-up visit by a nurse and a doctor from the Geriatric Team on the first weekday after discharge, nursing home staff could contact the team for at least one week after hospital discharge | Usual care |
| 289 | Pepera 2021 | To assess haemodynamic and physical function responses during a multicomponent group exercise program | A twice-weekly multicomponent exercise programme | Usual care |
| 290 | Pérez-Ros 2019 | To determine the impact of a preferred music listening group intervention upon the functional, cognitive, and emotional dimensions in residents | Musical stimulation sessions 5 x 60-minutes per week alongside usual occupational therapy | Usual care |
| 291 | Peterson 2016 | To demonstrate that a novel, minimally invasive programme not interfering with activities of daily living or socialization could lower methicillin-resistant Staphylococcus aureus (MRSA) disease. | Universal decolonization using intranasal mupirocin and a chlorhexidine bath performed twice (1 month apart) | Usual care |
| 292 | Pinazo-Clapes 2020 | To analyse the effectiveness of a non-pharmacological intervention on behavioural alterations and prescription of psychotropic drugs | Caregivers received 20 hours of a training program | Usual care |
| 293 | Rantz 2021 | To measure the impact of advanced practice nurses on quality measures scores of nursing homes in the Missouri Quality Initiative | Embedded full time advanced practice registered nurses in participating homes | N/A |
| 294 | Ray 2017 | To examine the use of music therapy for treatment of agitation and depressive symptoms for residents with dementia | Music therapy sessions three times a week | N/A |
| 295 | Rezola-Pardo 2019, 2020a, 2020b, 2022 | To determine whether the addition of simultaneous cognitive training to a multicomponent exercise programme offers further benefits to dual-task, physical and cognitive performance, psycho-affective status, quality of life and frailty | Multicomponent strength and balance exercises twice weekly | Dual-task training |
| 296 | Richter 2019 | To investigate a person-centred care approach, which has been successfully evaluated in homes in the UK, and adapted it to German conditions | Medication reviews, two hours of continuing medical education, educational interventions on person-centred care and a continuous supervision programme | Medication reviews and 2 hours of continuing medical education |
| 297 | Riggs 2020 | To explore whether direct access to dental hygienist instruments (for debridement/scaling) affected residents' perception of their oral health | The treatment group received a debridement by a hygienist and oral hygiene education | Received brushing, flossing and oral hygiene education |
| 298 | Roets-Merken 2018 | To evaluate the effectiveness of a nurse supported self-management programme to improve social participation of dual sensory impaired older adults | Nurse-supported self-management programme | Usual care |
| 299 | Rolland 2016 | To examine the effects of a global intervention comprising professional support and education for nursing home staff on quality indicators and functional decline and emergency department transfers of residents | IQUARE intervention to staff involving quality audit and feedback plus collaborative meetings between a hospital geriatrician and nursing home staff | Quality audit and feedback |
| 300 | Rolland 2020 | To assess whether systematic dementia screening of residents combined with multidisciplinary team meetings resulted in a lower rate of Emergency Department transfer at 12 months compared with usual care | Two multidisciplinary team meetings to identify residents with dementia and to discuss an appropriate care plan | Usual care |
| 301 | Romøren 2017 | To assess if a brief training programme in administrating intravenous fluids and antibiotics could reduce hospital transfers and ensure high quality care locally | A one-day educational programme for all health workers in the nursing homes including theory and practical training in intravenous treatment of dehydration and infection, run by two skilled nurses. After completing the training program, the nursing homes had competence to provide intravenous treatment locally | Usual care |
| 302 | Roos 2016 | To examine residents’ perceptions of empowerment, person-centred climate and life satisfaction before and after a caregiver intervention concerning the Swedish national fundamental values | Caregiver intervention was carried out using an interpretative approach with eight guided face-to-face seminars, where self-reflection and dialogue were used | Usual care |
| 303 | Rostad 2018 | To assess whether regular pain assessment using a pain assessment tool is associated with changes in pain scores and analgesic use in residents with severe dementia | Residents were assessed for pain twice weekly using Doloplus-2 pain scale | Usual care |
| 304 | Roughead 2022 | To assess the effectiveness of a pharmacist-led intervention using validated tools to reduce medicine-induced deterioration and adverse reactions | Pharmacist-led intervention using validated tools to detect signs and symptoms of medicine-induced deterioration which occurred every 8 weeks over 12 months | Usual care |
| 305 | Rutten 2022 | To investigate whether an electronic health record integrated decision tool, combined with supportive interventions, results in more appropriate antibiotic prescribing in nursing home residents with suspected urinary tract infection, without negative consequences for residents | Electronic Health Record-integrated decision tool and supportive interventions | Usual care |
| 306 | Ryuichi 2021 | To evaluate the impact of a computerised system on number of emergency transfers to and death in hospitals | New information and communication technology system which linked electronic health and care records between the nursing home and local clinic/primary care | Control data was from residents living in the home before implementation of the new information and communication technology system |
| 307 | Saal 2019 | To assess the feasibility of a new intervention to improve social participation in residents with acquired joint contractures | Participation Enabling Care in Nursing intervention uses a facilitation approach, which is a concerted, social process that focusses on evidence-informed practice change | Optimised standard care: in-house infromation session only, no mentoring or faciliation to support homes to overcome barriers to residents engaging in activities |
| 308 | Sackley 2015 & 2016 | To evaluate the clinical efficacy of an established programme of occupational therapy in maintaining functional activity and reducing further health risks from inactivity in residents living with stroke sequelae | Targeted three month programme of occupational therapy, goal setting, education of care home staff, and adaptations to the environment | Usual care |
| 309 | Sado 2020 | To evaluate the feasibility of conducting a definitive trial to assess the effectiveness of combined cognitive intervention | Learning therapy intervention (combination of cognitive training and stimulation) | Usual care |
| 310 | Saevareid 2019 | To improve resident participation in advance care planning in homes where most residents have some degree of cognitive impairment | The intervention included implementation support using a whole-ward approach where regular staff perform advance care planning and invite all patients and next of kin to participate | Usual care |
| 311 | Sahin 2022 | To assess the impact of Otago exercises on frailty and empowerment in residents | Strengthening, balancing exercises for 45 minutes, 3 days a week for 12 weeks, plus 30 minutes of walking the other 3 days of the week. 10 30-minute sessions of empowerment-based training | Usual care |
| 312 | Saint-Bryant 2020 | To explore the feasibility of a staff led intervention to help residents with dementia settle into a care home | Received SettleIn intervention a manualised staff-led programme of five modules designed to facilitate adjustment to care for new residents with dementia | Usual care |
| 313 | Samefors 2020 | To explore whether an intervention with active encouragement to spend time outdoors during summertime could increase the levels of vitamin D | Encouragement to go outside for 20-30 minutes between 11am and 3pm every day for two consecutive months | Usual care |
| 314 | Sampson 2020 | To develop and test feasibility of staff training intervention for agitation in people with severe dementia reaching end-of-life | Staff training intervention to improve care for agitation in people with severe dementia | N/A |
| 315 | Sampson 2021 | To indicate if definitive study of Better Health in Residents of Care Homes with Nursing intervention is warranted | Complex intervention to support healthcare and improve early detection and treatment of urinary tract and respiratory infections, chronic heart failure and dehydration, comprising: (1) ‘Stop and Watch (S&W)’ early warning tool for changes in physical health, (2) condition specific care pathway and (3) Situation, Background, Assessment and Recommendation tool to enhance communication with primary care. Implementation was supported by Practice Development Champions, a Practice Development Support Group and regular telephone coaching with external facilitators | Usual care |
| 316 | Sanchez 2021 | To evaluate the impact on medication consumption, of a booklet designed to aid physicians with prescriptions for residents | Booklet providing index of standard medication suitable for use in older nursing home residents and alternative drugs for specific situations | Usual care |
| 317 | Santagata 2021 | To evaluate the efficacy of doll therapy | Empathy doll given to person 2 hours am, 2 hours pm and PRN | Usual care |
| 318 | Santamaria 2018 | To investigate the clinical effectiveness of the application of the Mepilex Border Sacrum and Mepilex Heel dressings to prevent the development of facility acquired pressure injuries | Mepilex Border Sacrum dressing applied to their sacrum and a Mepilex Heel dressing, retained with Tubifast, applied to each heel. The interval between dressing changes was 3 days or as required if the dressing became soiled or dislodged | Usual care |
| 319 | Saredakis 2020 | To examine if Head Mounted Device for reminiscence therapy improved outcomes compared to use of laptop computer | Personalised videos in virtual reality viewed through head mounted device | Active control group - view personalised reminiscence content on a laptop. Passive control group - usual care. |
| 320 | Saredakis 2021 | To evaluate feasibility of using tailored reminiscence content through a virtual reality head mounted device | Tailored reminiscence virtual reality content administered via a head mounted device | N/A |
| 321 | Seemer 2021 & 2022 | To evaluate the effects of an individualized nutritional intervention on usual Protein Intake for residents at risk of malnutrition | Three intervention modules, a protein energy drink, a sweet and a savoury protein cream, in addition to usual nutritional care, offered at breakfast and lunch | N/A |
| 322 | Seleskog 2018 | To trial a new oral healthcare educational programme and to evaluate the effects on residents’ oral health | Weekly theoretical and hands-on guidance from dental hygienists on oral hygiene procedures and discussions on oral care routines | Usual care |
| 323 | Shaw 2018 | To evaluate change in antipsychotic medication rates in nursing homes receiving the communication education versus the corresponding state-wide average change | An educational programme to enhance communication in nursing home dementia care | Usual care |
| 324 | Shin 2015 | To examine the effects of doll therapy on residents' mood, behaviour, and social interactions | Doll therapy in the activity room | N/A |
| 325 | Sjögren 2022 | To evaluate effects of a person-centred and thriving intervention on residents´ experiences of thriving and person-centredness of the environment | Educational programme for nursing home staff | Two hour lecture on theory and framework of the intervention |
| 326 | Sloane 2020 | To determine if antibiotic prescribing can be reduced by a multicomponent antibiotic stewardship intervention implemented by medical providers and nursing staff and whether implementation is more effective if performed by a chain or a medical provider group | Pragmatic implementation trial: testing whether a multicomponent antibiotic stewardship intervention of known effectiveness could be implemented in practice and whether implementation by a nursing-home chain or medical-provider group was more effective | N/A |
| 327 | Sluggett 2020a & 2020b | To evaluate the impact of structured medication regimen simplification on medication administration times, falls, hospitalization, and mortality | Structured medication regimen simplification | Usual care |
| 328 | Smeets 2021 | To test the effectiveness of a multi-stage multidisciplinary intervention in reducing psychotropic medication prescription in residents with dementia | Structured and repeated multidisciplinary medication review supported by education and continuous evaluation | Usual care |
| 329 | Sprangers 2015 | To implement a brief, small-scale communication skills training programme to improve the quantity and quality of nursing aides’ communication with residents | Communication skills training for nursing aides | Usual care |
| 330 | Stacpoole 2015 | To evaluate the effects of the Namaste Care programme on the behavioural symptoms of residents with advanced dementia and their pain management | Namaste Care is a multidimensional intervention seeking to engage people with advanced dementia and enrich their quality of life through sensory stimulation, shared activity and increased social interaction. Comfort is a primary aim of the care programme, which includes formal pain assessment, as well as increasing care staff’s awareness and responsiveness to distress. The programme takes place 7 days a week with 2-hour sessions, morning and afternoon | N/A |
| 331 | Stensvik 2022 | To investigate the short-term effect of implementing a modified comprehensive geriatric assessment and regularly case conferencing in nursing homes on neuropsychiatric symptoms | Modified comprehensive geriatric assessment and monthly case conferencing | Usual care |
| 332 | Stephens 2020 | To assess the impact of night-time positioning equipment for adults with complex health conditions and postural asymmetry on their activities of daily living, posture, and quality of life | Prescription and provision of appropriate night-time postural-support equipment such as soft fibre wedges, neck support pillows, foot supports, and supine stabilisers | N/A |
| 333 | Stow 2015 | To define outcomes and optimise the design for an adequately powered definitive trial to compare the efficacy of established nutritional interventions in this setting | Two intervention groups: food-based intervention or oral nutritional supplement intervention | Usual care |
| 334 | Strauven 2019 | To investigate the impact of a complex multifaceted intervention on the appropriateness of prescribing for residents | The complex intervention consisted of repeated interdisciplinary case conferences (ICCs) involving the general practitioner, pharmacist, and nurse, aimed at performing a medication review for each resident included. The ICCs were supported by a blended training programme and local interdisciplinary meetings | Usual care |
| 335 | Sullivan 2017 | To determine the effects of story sharing on the health outcomes of depression and well-being of adults transitioning to a long-term care facility | Story sharing intervention | Usual care |
| 336 | Sultana 2021 | To evaluate the feasibility of Virtual Reality experience | Virtual reality experience for 30 minutes, five days per week for two weeks | N/A |
| 337 | Surr 2020 & 2021 | To investigate the clinical effectiveness and cost-effectiveness of Dementia Care Mapping™ for reducing agitation and improving care outcomes for people living with dementia | Dementia care mapping - a whole-home, practice development intervention to support the embedding of person-centred care | Usual care |
| 338 | Suzuki 2019 | To determine whether ultrasound-assisted prompted voiding care is more efficacious than conventional prompted voiding care for managing urinary incontinence | Ultrasound-assisted prompted voiding - caregivers regularly monitored bladder urine volume by an ultrasound device and prompted the resident to void when the volume reached close to the individually optimized bladder capacity | Conventional prompted voiding - caregivers asked resident every 2-3 hours if they had a desire to void and prompted them to void if they answered yes |
| 339 | Swales 2022 | To assess the feasibility of a definitive, randomised controlled trial using a resistance training intervention | Machine-based resistance training protocol three times per week | Wait-list control group |
| 340 | Tadrous 2020 | To evaluate the effectiveness of academic detailing in nursing homes targeting appropriate prescribing of antipsychotics | Academic detailing delivered by health professionals (eg, nurses or pharmacists) who arranged meetings (with administrators, physicians, pharmacists, nurses, and support workers), presentations, group visits (with 2-6 clinicians), and 1-on-1 visits (traditional academic detailing visits) | Usual care |
| 341 | Tappen 2020 | To evaluate a person-centred decision aid for residents and families about hospital transfers | Person-centred decision aid containing information on hospital transfers | Usual care |
| 342 | Taylor 2015 | To examine whether a group-based exergame programme could improve mobility in residents, including those with and without cognitive impairment | X-Box Kinect exergames, twice weekly | Usual care |
| 343 | Taylor 2018 | To evaluate the feasibility of a multi-faceted training intervention focused on person centred mobility care | Collaborative training programme in the use of person-centred approaches and mobility enhancing strategies during care including how to approach, interact and communicate with residents and how to promote the correct biomechanics for independent transfers | N/A |
| 344 | Teesing 2021 | To evaluate a hand hygiene intervention | A multimodal intervention to improve hand hygiene, including review of policies, e-learning, staff training, poster competition | Usual care |
| 345 | Telenius 2015a & 2015b | To investigate the effect of a high intensity functional exercise programme on the performance of balance in residents with dementia | High intensity functional exercises led by physiotherapists in small groups twice a week | Control participants met in groups at each nursing home twice a week for 50–60 minutes and were led by occupational therapists, nursing staff, volunteers or activity-leader. Control activities were light physical activity, reading, playing games, listening to music and conversations |
| 346 | Temkin-Greener 2017a & 2017b | To test the impact of palliative care teams on end-of-life outcomes | Improving Palliative Care Through Teamwork was a facility-level intervention involving a multicomponent strategy that included implementing facility based palliative care teams and providing staff with palliative and end-of-life geriatric training. Team development and staff training were followed by a 2-month long active intervention phase during which a gero-palliative care nurse practitioner interventionist rounded with the teams as they saw and/or discussed residents’ care. A passive phase of 8 months immediately followed, during which the nurse interventionist was available to further coach the team on as needed/requested basis | 11 homes randomised to control received usual care. A further 209 non-randomized facilities also provided control data |
| 347 | Ter Wee 2016 | To evaluate the effects of 8-week consumption of high protein oral nutritional supplements on renal function of nursing home residents in need of supplementation | Usual diet supplemented with personally prescribed amounts of oral nutritional supplements | High protein standard oral nutritional supplement |
| 348 | Testad 2016 | To evaluate the effectiveness of a tailored 7-month training intervention “Trust Before Restraint,” in reducing use of restraint, agitation, and antipsychotic medications in residents with dementia | Trust before Restraint intervention for care staff delivered using training manual and small group education | Usual care |
| 349 | Thodberg 2016a & 2016b | To evaluate the effects of biweekly dog visits on sleep patterns and the psychiatric wellbeing of residents | Bi-weekly 10-minute visits from a person accompanied by either a dog, robot seal (PARO), or soft toy cat on behaviour and interaction | N/A |
| 350 | Travers 2017 | To assess the feasibility and acceptability of a behavioural activities intervention | Behavioural activities intervention - mental health therapist worked with residents to identify activities they wished to engage with and develop a care plan. Care staff were trained and supported to use care plans to facilitate increased participation in activities | Walking and Talking intervention |
| 351 | Treusch 2015 | To evaluate an interdisciplinary occupational and sport therapy intervention for dementia residents suffering from apathy | Biographically orientated mobilization intervention once a week for 15 minutes provided by an occupational therapist and a sport therapist in privacy of residents' rooms | Usual care |
| 352 | Tropea 2022 | To test whether a specialist palliative/end of life training intervention would reduce unplanned transfers to hospital for residents with dementia | Online training modules for nursing and care staff, based around case studies with videos | Usual care |
| 353 | Tsai 2018 | To examine the initial effects and estimated effect size of a computer-based simulation education programme on certified nursing assistants’ level of assistance when dressing residents with dementia, and on residents’ dressing performance | One hour education module delivered by a research assistant followed by an additional two-hour intervention using a video simulator that enabled nursing assistants to practice level of assistance skills | One hour education module delivered by a research assistant |
| 354 | Tsugawa 2020 | To test the cognitive and physical effects of an exercise intervention | Group exercise, 40 minutes twice per week | Usual care |
| 355 | Tylner 2016 | To evaluate the effects of an energy-dense oral supplement on nutritional status, food intake, and physical function in residents | Energy-dense formula – oleic and linoleic acid emulsion enriched with protein and micro-nutrients (Calogen Extra, Nutricia) 30mls distributed three times daily | Cross-over design |
| 356 | Valiani 2017 | To explore the feasibility and acceptability of a new home-based exercise technology among older adults and to evaluate its efficacy on physical performance measures | Light intensity exercise (aerobic, strength and balance) 30 minutes twice weekly, using Jintronix technology | N/A |
| 357 | Van Bogaert 2016 | To investigate the effect of a standardized individualized intervention based on the transformational reminiscence model on depressive symptoms, cognition and behaviour for older people with mild to moderate dementia | Standardized individual reminiscence intervention based on the SolCos model delivered by trained volunteers | Usual care |
| 358 | van Dam 2020 | To test if paracetamol improves quality of life, discomfort, pain, and neuropsychiatric symptoms | Two periods of treatment for six weeks (paracetamol / placebo), with a one-week wash out period. | Placebo |
| 359 | Van den Block 2019, 2020 & Miranda 2021 | To evaluate the effect of the Palliative Care for Older People Steps to Success Program on resident and staff outcomes | Programme of palliative Care for Older People Steps , using a train-the-trainer approach, an external trainer supports staff in nursing homes to introduce a palliative care approach over the course of 1 year following a 6-steps programme | Usual care |
| 360 | Van Haitsma 2015 | To test the effectiveness of individualized activities, led by certified nursing assistants, to increase positive and reduce negative affect and behaviour among residents with dementia | Two intervention groups: 1) Attention Control group – standardized one-to-one activities with certified nursing assistants (CNAs). 2) Individualized Positive Psychosocial Intervention group – a CNA-led activity matched to their interests and ability | Usual care |
| 361 | van Leen 2018 | To test the pressure ulcer preventive effect of a pressure-relieving, shear stress diminishing, and microclimate-controlling skin interface multilayer support system (Bedcare; Sense Textile‘s-Hertogenbosch, the Netherlands) compared with a viscoelastic foam mattress alone | High-quality viscoelastic foam mattress with newly developed multilayer system | High-quality viscoelastic foam mattress covered with a cotton sheet |
| 362 | van Welie 2016 | To evaluate the effect of warning symbols in combination with education on the frequency of erroneously crushing medication | A set of warning symbols printed on each patient’s unit dose packaging indicating whether or not a medication could be crushed as well as education of staff (lectures, newsletter and poster) | N/A |
| 363 | van Wietmarschen 2020 | To evaluate the effect of probiotics on antibiotic-associated diarrhoea | Administration of multispecies probiotics between January and September 2018 | N/A |
| 364 | Van Wymelbeke 2016 | To compare the effects of a 12-week nutritional intervention, in which an innovative protein-and-energy-enriched brioche, an oral nutritional supplement or a usual breakfast were eaten, on food intake and nutritional status | Two intervention groups: enriched-brioche (brioche group), ONS (supplement group) daily | Usual care |
| 365 | Van Wymelbeke 2020 | To evaluate an intervention to increase sensory quality and variety on food intake and meal enjoyment of residents | Three intervention groups: Quality+ recipes were improved according to sensory preference, Variety+ residents were offered a variety of main dishes and several condiments throughout the mean and Quality&Variety combining the two other interventions | N/A |
| 366 | Veleva 2020 | To evaluate the effect of UVB treatment (vs standard, vitamin B) on resident wellbeing | Half-body UVB light irradiation twice weekly with 1 standard erythema dose | 5600 international units cholecalciferol supplementation once a week |
| 367 | Verreault 2018 | To evaluate the impact of a multidimensional intervention to improve quality of care and quality of dying in advanced dementia | The intervention had five components: (1) training programme to physicians and nursing staff, (2) clinical monitoring of pain using an observational pain scale, (3) implementation of a regular mouth care routine, (4) early and systematic communication with families about end-of-life care issues with provision of an information booklet, and (5) involvement of a nurse facilitator to implement and monitor the intervention | Usual care |
| 368 | Viscogliosi 2017 | To assess efficacy and safety of citalopram compared to quetiapine and olanzapine for the treatment of agitation in residents with Alzheimer disease | Three intervention groups: Citalopram (25) or Quetiapine (25) or Olanzapine (25) | N/A |
| 369 | Visscher 2020 | To evaluate if offering fruit and vegetable finger food is feasible | Daily provision of fruit and vegetable finger food at 4pm | N/A |
| 370 | Visser 2021 | To determine the effect of a deprescribing algorithm undertaken by trained nursing home physicians | Deprescribing of statins and proton pump inhibitors for nursing home residents | N/A |
| 371 | Walker 2016 | To explore the feasibility of implementing and evaluating the Guide to Action Care Home fall prevention intervention | Guide to Action Care Home fall prevention intervention training and support | Usual care |
| 372 | Walker 2020 | To determine effectiveness of an intervention to increase prevalence of adequate vitamin D supplement use | A multifaceted, interdisciplinary knowledge translation intervention (a local champion to drive implementation, the use of an expert opinion leader, dissemination of educational material, educational outreach visits, feedback on medication chart audits and locally facilitated quality improvement) | Stepped-wedge design |
| 373 | Wallace 2016 | To determine whether a qualified dental hygienist could improve oral health outcomes for residents | Senior Smiles programme: a dental hygienist undertook an intervention with residents including oral health risk assessments, oral healthcare plans and referrals for treatment where needed | N/A |
| 374 | Wang 2018 | To assess the feasibility of conducting a large clinical trial to evaluate the effectiveness of probiotics to reduce influenza and other respiratory virus infections | Probiotics—2 capsules of Lactobacillus rhamnosus GG (estimated 10 billion colony forming units of L. rhamnosus GG per capsule) | Placebo (calcium carbonate) |
| 375 | Watson 2019 | To evaluate and compare effectiveness of Lavender and Lemon Balm essential oils on the agitated behaviour of residents with and without dementia | Test of treatment with Lavender or Lemon Balm or Placebo (sunflower oil) once daily for two weeks | Cross-over design |
| 376 | Wauters 2021 | To explore the efficacy, feasibility and acceptability of a complex multifaced intervention supporting multidisciplinary medication reviews | The OptiMEDs intervention is a multifaceted intervention with the aim of holding a multidisciplinary medication review, with input of nurses, pharmacists and GP, supported by the OptiMEDs tool | Usual care |
| 377 | Weatherall 2019 | To determine whether assigning a dedicated general practitioner to a nursing home reduces hospitalizations and readmissions | Seven nursing homes received an intervention introducing a single General Practitioner to be assigned to the home. The programme paid a GP to meet weekly with the home staff to discuss the clinical needs of each of the GP's patients, and to provide training that could be applied to all residents. The meetings ranged from 1.5 to 3 hours per week and were in addition to the time spent treating patients | Usual care |
| 378 | Weintraub 2018 | To determine if recommended mouth care practices provided by staff could improve residents’ oral hygiene and denture outcomes | The intervention consisted of training nursing home staff in the Mouth Care Without a Battle protocol, and providing support in its use for two years | Usual care |
| 379 | Werner 2017 | To compare the effects of interactive music therapy and recreational group singing on depression levels of residents | Biweekly 40-minute interactive music therapy, free setting | Weekly 1.5 hour recreational group singing |
| 380 | Wesenberg 2019 | To investigate if the inclusion of an animal adds value to psychosocial interventions for people with dementia | Animal assisted intervention therapy programme conducted by trained dog owners and involving their dogs in weekly group sessions following a standardised manual | Control exercise intervention in weekly sessions without the dog, delivered by volunteers |
| 381 | Westerhof 2018 | To assess the effects of an autobiographical memory intervention on the prevention and reduction of depressive symptoms | Precious Memories – autobiographical intervention – 8 X 45 min sessions | Unstructured contacts with a volunteer for 45 minutes per week |
| 382 | Whitney 2017 | To evaluate the safety, acceptability, and feasibility of a multi-factorial intervention to prevent falls in older adults with cognitive impairment and provide an estimate of its efficacy | Assessment of falls risk factors followed by tailored intervention – dementia care mapping, geriatric assessment, occupational therapy input, twice-weekly exercise | Usual care |
| 383 | Wijnen 2015 | To compare the efficacy of an individualized cholecalciferol loading dose regimen and a daily dose regimen of cholecalciferol 800 IU in reaching 25-OH vitamin D (25-OHD) levels [75 nmol/l] | Individualized dose of Vitamin D | Standard daily dose of Vitamin D |
| 384 | Wilchesky 2018 | To test the feasibility of an interdisciplinary knowledge exchange intervention using a medication review guidance tool categorizing medications as either “generally”, “sometimes” or “exceptionally” appropriate for residents with severe dementia | Information leaflet for residents’ families, a 90-minute knowledge exchange (KE) session for general practitioners , pharmacists and nurses focusing on the medication review guidance tool, a medication review by the pharmacists for participating residents with ensuing team discussion on medication changes, and a post-intervention KE session to obtain feedback from team staff | N/A |
| 385 | Williams 2016 | To describe the use of video game technology, specifically the Nintendo Wii, to determine if there is a relationship among personal factors, perceived barriers, perceived benefits, perceived self-efficacy, and physical activity; and to examine the effects of this video game technology perceived barriers, perceived benefits, and perceived self-efficacy for physical activity using a 6-week intervention | Educational component followed by 30 minutes of Nintendo Wii game play | N/A |
| 386 | Wilson 2019 | To optimise hydration care for frail older people in a care home setting | Quality improvement methods to develop and test interventions to extend drinking opportunities and choice, changes were made and evaluated using Plan-Do-Study-Act (PDSA) cycles | N/A |
| 387 | Wogamon 2016 | To explore the effect of a 1-hour Certified Nursing Assistant education programme about early identification, treatment, and prevention of Pressure Ulcers (PU) on PU knowledge, PU incidence, and PU prevention interventions, including skin checks | Education programme - 1hour evidence-based educational intervention for Certified Nursing Assistants | N/A |
| 388 | Woloszyn 2021 | To assess the influence of physical exercises with dance movement therapy elements on strength and other fitness components of the upper limbs and the overall functional performance of residents in wheelchairs compared to standard exercise programmes and usual care | Dance movement therapy 30-minute session, two times a week | Usual care |
| 389 | Wren 2016 | To examine the effects of life review on daily activities, social participation, outlook on life, and perceptions of living in a nursing home measured by life satisfaction and quality of life in older adults | Group intervention with a weekly topic, memory prompt and relation questions to provide organisation, facilitation and structure to the life review group. Topics covered were childhood, adolescence, family and home, adulthood, remembering the past through song and summary of life | N/A |
| 390 | Wylie 2017 | To establish the feasibility of a future phase III multicentre randomised controlled trial designed to establish the clinical effectiveness of the remodelled multifaceted podiatry intervention | Podiatry intervention: core podiatry care, foot and ankle exercises, orthoses, and footwear provision | Core podiatry only |
| 391 | Yamamoto 2019 | To clarify the influence of consuming yogurt fermented with *Lactobacillus delbrueckii ssp. bulgaricus* OLL1073R-1 (1073R-1-yogurt) on influenza virus-bound salivary immunoglobulin A levels | Yogurt fermented with Lactobacillus delbrueckii ssp. bulgaricus OLL1073R-1 (1073R-1-yogurt) | Control yoghurt fermented with another Lactobacillus |
| 392 | Yap 2022 | To investigate the clinical effectiveness of three nursing-home-wide repositioning intervals (2-, 3-, or 4-hour) without compromising pressure injury incidence | Repositioning interval every 2 hours | 3-hour or 4-hour repositioning interval |
| 393 | Yasuda 2017 | To assess the effects of care staff training based on person-centred care and dementia care mapping on the quality of life of residents with dementia | Staff training based on person centred care and dementia care mapping | N/A |
| 394 | Yeşilyaprak 2016 | To investigate the effects of virtual-reality-based balance exercises on balance and fall risk in comparison to conventional balance exercises in residents | Virtual Reality exercise 3 X per week (5 minutes warm-up, 35–45 minutes training, 5 minutes cool-down) | Conventional exercise 3 X per week (5 minutes warm-up, 35–45 minutes training, 5 minutes cool-down) |
| 395 | Yücel 2019 | To investigate the effects of hand massage and therapeutic touch on comfort and anxiety in residents | Two intervention groups: hand massage and therapeutic touch | Usual care |
| 396 | Zimmerman 2020 | To evaluate the effectiveness of Mouth Care Without a Battle, a programme that increases staff knowledge and attitudes regarding oral hygiene, changes mouth care, and improves oral hygiene, in reducing the incidence of pneumonia | Mouth Care Without a Battle is a standardized programme that teaches that mouth care is health care, provides instruction on individualized techniques and products for mouth care, and trains caregivers to provide care to residents | Usual care |

Abbreviations: RCT – randomised controlled trial; NR – not reported

**Appendix 6**

**Supplementary Table 7: Complete list of scale based outcome measures by frequency of use**

| **Outcome measure** | **Number of uses** |
| --- | --- |
| Neuropsychiatric Inventory (NPI)  [Neuropsychiatric Inventory Nursing Home Version] | 53  [20] |
| EuroQoL (EQ-5D) | 43 |
| Mini Mental State Examination (MMSE) | 41 |
| Cohen Mansfield Agitation Inventory (CMAI) | 37 |
| Geriatric Depression Scale (GDS) | 30 |
| Cornell Scale for Depression in Dementia (CSDD) | 29 |
| Barthel Index (BI) | 27 |
| Timed-up-and-go-Test (TUGT) | 26 |
| Quality of Life in Late Stage Dementia (QUALID) | 21 |
| Quality of Life in Alzheimer’s Disease (QOL-AD) | 19 |
| Short Physical Performance Battery (SPPB) | 18 |
| Berg Balance Scale (BBS) | 12 |
| Quality of Life for People with Dementia (QUALIDEM) | 12 |
| Dementia Quality of Life (DEMQOL) | 11 |
| Short Form Health Survey score | 11 |
| Pittsburgh Sleep Quality Index (PSQI) | 10 |
| Palliative care Outcome Scale (POS) | 9 |
| Tinetti Performance Oriented Mobility Test | 9 |
| Dementia Care Mapping (DCM) | 8 |
| Montreal Cognitive Assessment (MoCA) | 8 |
| Trail making test | 8 |
| Hospital Anxiety and Depression Scale (HADS) | 7 |
| Mini Nutritional Assessment (MNA) | 7 |
| Activities of Daily Living (ADL) score | 6 |
| Apathy Evaluation Scale (AES) | 6 |
| Falls Efficacy Scale International (FES-I) | 6 |
| Observed Emotion Rating Scale (OERS) | 6 |
| WHO International Classification of Functioning, Disability and Health (ICF) | 6 |
| Brief Pain Inventory (BPI) | 5 |
| Confusion Assessment Method (CAM) | 5 |
| Functional Independence Measure [FIM] | 5 |
| Geriatric Anxiety Inventory (GAI) | 5 |
| Medication Appropriateness Index (MAI) | 5 |
| Mobilization-Observation-Behaviour-Intensity-Dementia Pain Scale (MOBID) | 5 |
| Pain Assessment in Advanced Dementia (PAINAD) | 5 |
| Addenbrooke's Cognitive Evaluation | 4 |
| Charlson Comorbidity Index (CCI) | 4 |
| Client Services Receipt Inventory (CSRI) | 4 |
| Clock Drawing Test (CDT) | 4 |
| Comfort Assessment in Dying with Dementia (CAD-EOLD) | 4 |
| De Jong Gierveld Loneliness Scale (DJGLS) | 4 |
| Functional Linguistic Communication Inventory (FLCI) | 4 |
| Instrumental Activities of Daily Living (IADL) score | 4 |
| Mucosal Plaque Index (MPS) | 4 |
| Physical Activity and Mobility in Residential Care scale (PAM-RC) | 4 |
| Plaque Index (PI) | 4 |
| Senior Fitness Test (SFT) | 4 |
| World Health Organization Quality of Life Scale for Older People (WHOQOL-OLD) | 4 |
| Abbey Pain Scale | 3 |
| Beck Depression Inventory | 3 |
| Beers’ Criteria 2015 | 3 |
| Center for Epidemiologic Studies Depression Scale (CES-D) | 3 |
| Clinical Dementia Rating (CDR) scale | 3 |
| Frontal Assessment Battery (FAB) | 3 |
| General Comfort Questionnaire (GCQ) | 3 |
| Impact of Vision Impairment questionnaire | 3 |
| Interpersonal Support Evaluation List (ISEL) | 3 |
| Katz index of Activities of Daily Living | 3 |
| Montgomery and Asberg Depression Rating Scale (MADRS) | 3 |
| Perceived Stress Scale | 3 |
| Physical Self-Maintenance Scale (PSMS) | 3 |
| Quality of Interactions Schedule (QUIS) | 3 |
| Satisfaction with Life Scale (SWLS) | 3 |
| Silness & Löe index | 3 |
| STOPP-START v2 criteria and AGS 2015 Beers’ Criteria | 3 |
| Visual Analogue Scale (VAS) for pain | 3 |
| Alzheimer’s Disease Cooperative Study Activities of Daily Living Inventory | 2 |
| Alzheimer’s Disease-Related Quality of Life (ADRQL) Scale | 2 |
| Beck Dressing Performance Scale | 2 |
| Bedford Alzheimer Nursing Severity Scale (BANSS) | 2 |
| Brief Agitation Rating Scale (BARS) | 2 |
| Brief Interview of Mental Status (BIMS) | 2 |
| Brief Psychiatric Rating Scale (BPRS) | 2 |
| California Verbal Learning Tests | 2 |
| Camberwell Assessment of Need for the Elderly | 2 |
| Canadian Occupational Performance Measure (COPM) | 2 |
| Clinical Global Impression of Change (CGIC) | 2 |
| Cognitive Performance Scale (CPS) | 2 |
| Dartmouth COOP Scales of Functioning | 2 |
| De Morton Mobility Index (DEMMI) | 2 |
| Dementia Screening Scale (DSS) | 2 |
| Depression Anxiety Stress Scale (DASS 21) | 2 |
| Doloplus-2 behavioural pain assessment scale | 2 |
| Domus Medica guideline sleep disorders | 2 |
| Drug Burden Index (DBI) | 2 |
| Edmonton Frailty Scale | 2 |
| Elderly Inspection Form | 2 |
| End-of-Life in Dementia Scale Comfort Assessment while dying (EOLD-CAD) | 2 |
| End-of-Life in Dementia–Satisfaction With Care scale (EOLD-SWC) | 2 |
| European Organization for the Research and Treatment of Cancer EORTC score | 2 |
| Functional Ambulation Classification (FAC) | 2 |
| Generalized Anxiety Disorder scale | 2 |
| Gingivitis Index (GI) | 2 |
| Goldberg Anxiety and Depression Scale | 2 |
| Investigating Choice Experiences Capability instrument (ICECAP) | 2 |
| Iowa Pain Thermometer | 2 |
| Lawton Instrumental activities of daily living scale | 2 |
| Life Satisfaction Scale | 2 |
| Numerical Rating Scale (NRS) | 2 |
| Older People’s Quality of Life (OPQOL-Brief) questionnaire | 2 |
| Papillary Bleeding Index | 2 |
| Patient Health Questionnaire (PHQ-9) | 2 |
| Pearlin Mastery Scale (PMS) | 2 |
| Person-centered Climate Questionnaire | 2 |
| Quality of Dying in Long Term Care (QOD-LTC) | 2 |
| Queen Elizabeth Behavioural Assessment Graphical Scale (QEBAGS) – adapted | 2 |
| Questions from validated instruments: Bel Rai | 2 |
| Questions from validated instruments: Bel Rai; OPQOL-35; RAND 36 | 2 |
| Rapid assessment of physical activity (RAPA) | 2 |
| Rating Anxiety in Dementia Scale (RAID) | 2 |
| Resistiveness to Care Scale (RTC-r) | 2 |
| Revised Oral Assessment Guide (ROAG) | 2 |
| Rey Auditory Verbal Learning Test (RAVLT) | 2 |
| Root Caries Index (RCI) | 2 |
| Satisfaction with Care at End-of-Life with Dementia (SWC-EOL D) | 2 |
| Screening Tool for Older Persons’ Potentially Inappropriate Prescriptions (STOPP criteria) | 2 |
| Severe Impairment Battery (SIB) | 2 |
| Six-item cognitive impairment test: (6-CIT) | 2 |
| Sleep Disorder Inventory (SDI) | 2 |
| Stroop colour-word test | 2 |
| Symptom Management for End-of-Life Care in Dementia (SM-EOLD) | 2 |
| Visual Analogue Scale (VAS) for quality of life | 2 |
| A.Di.CO scale derived from DISCO scale | 1 |
| Activities of Daily Living - 28-point scale | 1 |
| Activities of Daily Living (ADL) questionnaire | 1 |
| Activities of daily living dependencies | 1 |
| Activities of Daily Living Short Form (ADLSF) | 1 |
| Activity behaviour checklist | 1 |
| Activity Card Sort (ACS) | 1 |
| Adverse Drug Event Geriatric Risk Score | 1 |
| Agitated Behavior Scale (ABS) | 1 |
| Alabama Life Space Assessment (UAB LSA) | 1 |
| Algase Wandering Scale (AWS) | 1 |
| Alzheimer’s Disease Assessment Scale, cognitive subsection (ADAS-cog) | 1 |
| Anatomical Therapeutic Chemical codes | 1 |
| Anxiety and Depression Goldberg Scale | 1 |
| Asian working group for Sarcopenia criteria | 1 |
| Assessment Scale of Adaptation Difficulty for the Elderly | 1 |
| Associative learning | 1 |
| Attentive matrices | 1 |
| Attitude to Environmental Tasks Questionnaire (AETQ) | 1 |
| Autobiographic Memory Test | 1 |
| Autonomie Gérontologie Group Iso Resources | 1 |
| Axis 3: Past Memory | 1 |
| Backwards Corsi Blocks Test (BCBT) | 1 |
| Balance Outcome Measure for Elder Rehabilitation (BOOMER) | 1 |
| Beck Anxiety Inventory | 1 |
| Beck Hopelessness Scale (BHS) | 1 |
| Beers’ list (2015 edition) and the Norwegian General Practice – Nursing Home criteria (NORGEP-NH) | 1 |
| Behavioral Observation Scale for Intramural Geriatric Psychiatry | 1 |
| Behavioral Regulation in Exercise Questionnaire-2 (BREQ-2) | 1 |
| Block test (BBT) | 1 |
| Brief Cognitive Rating Scale | 1 |
| Brief Measure for Assessing Generalised Anxiety Disorder (GAD-7) | 1 |
| Brief Sense of Community Scale (BSCS) | 1 |
| Bristol Activities of Daily Living (ADL) scale | 1 |
| Bristol Stool Form Scale (BSF) | 1 |
| Care Dependency Scale (CDS) | 1 |
| Care Need Scales (CNS) | 1 |
| Category Verbal Fluency | 1 |
| Centres for Medicare and Medicaid Services Quality Measures for long-stay nursing home residents | 1 |
| Challenging Behaviour Scale | 1 |
| Change in Health and End Stage Disease, Signs and Symptoms (CHESS) | 1 |
| Choice reaction time | 1 |
| Choke risk score | 1 |
| Client Satisfaction Questionnaire (CSQ-8) | 1 |
| Clinical Frailty Scale (CFS) | 1 |
| Coding and Symbol Search test on the Wechsler Adult Intelligence Scale, Fourth Edition (WAIS-IV) | 1 |
| Communication Outcome After Stroke Scale (COAST) | 1 |
| Consortium to Establish a Registry for Alzheimer's Disease (CERAD) Plus test battery | 1 |
| Constructive Apraxia test | 1 |
| Continuing Care Activity Measure (CCAM) | 1 |
| Criterion Time for Certification of Needed Long-Term Care (CT for CNLTC) | 1 |
| d2 Test of attention | 1 |
| Dartmouth COOP Quality of Life chart | 1 |
| Decisional Conflict Scale | 1 |
| Degree of Daily Life Independence Score for People with Dementia (DDLIS-PD) | 1 |
| Delirium Index | 1 |
| Delirium Observation Screening Scale (DOSS) | 1 |
| Dementia Mood Assessment Scale (DMAS)-17 item | 1 |
| Denture Plaque Index (DPI) | 1 |
| Depression Inventory 8b | 1 |
| Depression Rating Scale (DRS) | 1 |
| Digit Span (DS) | 1 |
| Digit Span Task Backward (DSF) | 1 |
| Digit Span Task Forward (DSF) | 1 |
| Disability Assessment for Dementia (DAD) | 1 |
| Discomfort scale - Dementia of Alzheimer type (DS-DAT) | 1 |
| Dressing Assessment Guide (DAG) | 1 |
| Dry skin area and severity index (DASI) | 1 |
| Dry Skin Score five point scale | 1 |
| DSM-5 - Diagnostic and Statistical Manual of Mental Disorders, Fifth Edition | 1 |
| DSM-IV criteria- structured clinical interview | 1 |
| DSM-V Disorders – Clinician Version (SCID-5-CV) - structured clinical interview | 1 |
| Dutch Loneliness Scale | 1 |
| Dutch Mental Health Continuum-Short Form (MHC-SF) | 1 |
| e Index of Relocation Adjustment Scale (IRA) | 1 |
| Eagerness scale | 1 |
| Eating Behavior Scale (EBS) | 1 |
| Eating Validation Scheme (EVS) | 1 |
| Edinburgh Feeding Evaluation in Dementia (EdFED) | 1 |
| Edmonton Symptom Assessment System (ESAS) | 1 |
| Elderly Empowerment Scale | 1 |
| Elderly Mobility Scale (EMS) | 1 |
| End-of-Life in Dementia–Comfort Assessment in Dying scale (EOLD-CAD) | 1 |
| Epworth Sleepiness Scale (ESS) | 1 |
| EQ-VAS (European QoL–visual analogue scale) | 1 |
| Erlangen test of Activities of Daily Living | 1 |
| Essen questionnaire on age and sleepiness (EFAS) | 1 |
| European Working Group on Sarcopenia in Older People criteria | 1 |
| Everyday Problem Test (EPT) | 1 |
| Exercise Benefits and Barriers Scale | 1 |
| Experience Sampling Method (ESM) | 1 |
| Face–name learning | 1 |
| FACES Pain Scale | 1 |
| Facial Action Coding System | 1 |
| Facial pain score | 1 |
| Falls related impulsive behaviour scale | 1 |
| Family Perception of Care Scale (FPCS) | 1 |
| Family Perception of Physician-Family Communication (FPPFC) | 1 |
| Fastest Reaction Time (FRT) | 1 |
| Fear of Falling (FOF) | 1 |
| Figure–word pairing | 1 |
| Flourishing Scale | 1 |
| Foot Care Behavior Scale (FCBS) | 1 |
| Foot Care Knowledge Level Form (FCKLF) | 1 |
| Forward Corsi Blocks Test (FCBT) | 1 |
| Frailty and Injuries: Cooperative Studies of Intervention Techniques-4 scale (FICSIT-4) | 1 |
| Frailty Index scores change | 1 |
| French Clinical Pharmacy Society guidelines | 1 |
| Fried Frailty Total Score (0-5) | 1 |
| Fried MLTAQ(kcal·wk-1): Minnesota Leisure Time Activity Questionnaire Shortened Version | 1 |
| Fried's Frailty Phenotype (FFP) | 1 |
| Fullerton Functional Fitness Test | 1 |
| Functional Assessment Staging (FAST) | 1 |
| Functional Reach Test (FRT) | 1 |
| FunDepScore | 1 |
| G8 score | 1 |
| General Medical Health Rating (GMHR) scale | 1 |
| Geriatric Oral Health Assessment Index (GOHAI) | 1 |
| Gingival bleeding index (GBI) | 1 |
| Gingival Index for Long-Term Care (GI-LTC) | 1 |
| Global Deterioration Scale | 1 |
| Global self-assessment of pain single question | 1 |
| Goal Attainment Scaling (GAS) | 1 |
| Gottfries cognitive scale | 1 |
| Gottfries-Bråne-Steen scale (GBS) | 1 |
| Grocery list learning | 1 |
| Gugging Swallowing Screen (GUSS) | 1 |
| Hasegawa’s Dementia Scale-Revised (HDS-R) | 1 |
| Health Utilities Index Mark 2/3 (HUI 2/3) | 1 |
| Health-Related Quality-of-Life Instrument (15-dimensions) | 1 |
| Hearing Handicap Questionnaire (HHQ) | 1 |
| Hill step test | 1 |
| Hoad-Reddick classification | 1 |
| Homecare Measure of Engagement (Staff report) | 1 |
| Horikawa Scale (2009) | 1 |
| Incontinence Quality of Life Scale | 1 |
| Incontinence Severity Index (ISI) | 1 |
| Index of Social Engagement (ISE) | 1 |
| International Guidelines Severity Scoring | 1 |
| Iowa Oral Performance Instrument (IOPI) | 1 |
| Itaki Fall Risk Scale | 1 |
| Jigsaw Puzzle Test | 1 |
| Karolinska Sleepiness Scale | 1 |
| Laroche criteria | 1 |
| Lee Scale | 1 |
| Leg-o-Meter | 1 |
| Letter Verbal fluency (LVF) | 1 |
| Letter-Number Sequencing (LNS) | 1 |
| Level of Assistance Use Tool (LoA) | 1 |
| Life Satisfaction Index-Z (LSI-Z) | 1 |
| Life Satisfaction Questionnaire (LSQ) | 1 |
| Löe & Silness index | 1 |
| Long Term Care Quality of Life Assessment (LTC-QoL) | 1 |
| M.D. Anderson Dysphagia Inventory (MDADI) | 1 |
| Major Depression Inventory (MDI) | 1 |
| Malnutrition Universal Screening Tool (MUST) records | 1 |
| Manchester and Oxford Universities Scale for the Psychopathological Assessment of Dementia (MOUSEPAD) | 1 |
| Medical Outcomes Study- Social Support Survey (MOS-SSS) | 1 |
| Meditation Breath Attention Scores (MBAS) – adapted version | 1 |
| Menorah Park Engagement Scale | 1 |
| Minnesota Toileting Skills Questionnaire (MTSQ) | 1 |
| MobDepScore | 1 |
| Modified Alzheimer Disease Cooperative Study - Clinical Global Impression of Change (mADCS-CGIC) scale score | 1 |
| Modified Client Service Receipt Inventory | 1 |
| Modified Falls Efficacy Scale (MFES) | 1 |
| Modified Functional Reach Test | 1 |
| Modified Gingival Index | 1 |
| Modified Katz Index of Independence in Activities of Daily Living (Katz PADL-index) | 1 |
| Modified sulcus bleeding index (MSB) | 1 |
| Movie for the Assessment of Social Cognition | 1 |
| MRC Database of Instruments for Resource Use Measurement’ (DIRUM) | 1 |
| Multidimensional Observation Scale for Elderly Subjects | 1 |
| Multidimensional Scale of Perceived Social Support (MSPSS) | 1 |
| Mutuality Scale of the Family Caregiving Inventory | 1 |
| National Healthcare Safety Network criteria applied to clinical symptoms | 1 |
| National Institute for Health and Clinical excellence dementia guidelines and Alzheimer's society antipsychotic guidance | 1 |
| Nottingham Health Profile | 1 |
| NPUAP/EPUAP/PPPIA scale | 1 |
| Nursing home activities of daily living scale | 1 |
| Nursing Home Falls Self-Efficacy Scale (NHFSS) | 1 |
| Nursing Home Hearing Handicap Index (NHHI) | 1 |
| Nursing Home Life Space Diameter | 1 |
| Nursing Outcomes Classification (NSO) scale | 1 |
| O’Leary plaque index | 1 |
| Object list learning | 1 |
| Observational Measurement of Engagement Tool (OME) | 1 |
| Observed Emotions Rating Scale (OERS) | 1 |
| Oral Assessment Guide (OAG) | 1 |
| Oral Health Assessment Tool (OHAT) | 1 |
| Oral Health Improvement Profile (OHIP-5) | 1 |
| Oral Hygiene Index (OHI) | 1 |
| Pain Assessment Checklist for Seniors with Limited Ability to Communicate (PACSLAC-F) | 1 |
| Pain in at least two measurements of Brief Pain Inventory/Numerical Rating Scale | 1 |
| Pain Medication Appropriateness Scale (PMAS) | 1 |
| Pain numerical rating scale | 1 |
| Pain scale (PAIN) | 1 |
| Pain thermometer | 1 |
| Particular Scales assessment tool (participation subscale) | 1 |
| Pathway Span Test (PST) | 1 |
| Patient Autonomy Questionnaire (PAQ) | 1 |
| Patient Empowerment Scale | 1 |
| Perceived Stress Questionnaire (PSQ) | 1 |
| Perception of Bladder Condition (PBC) - single question global PROM | 1 |
| Person-Centred Care Assessment Tool | 1 |
| Person-Environment Apathy Rating (PEAR) apathy subscale | 1 |
| Personal Beliefs about Memory (PBMI) | 1 |
| Personalised outcomes via modified Goal Attainment Scaling (GAS) | 1 |
| Pharmaceutical Care Network Europe classification scheme | 1 |
| Philadelphia Geriatric Center Affect Rating Scale | 1 |
| Philadelphia Geriatric Center Morale Scale (PGCMS) | 1 |
| Physical Activity Survey for Long Term Care (PAS-LTC) | 1 |
| Physical Mobility Scale (PMS) | 1 |
| Physiotherapy Clinical Outcome Variables | 1 |
| Pittsburgh Agitation Scale (PAS) | 1 |
| Plaque control record (PCR) | 1 |
| Plaque Index for Long-Term Care (PI-LTC) | 1 |
| Pleasant Events Schedule - Nursing Home Version | 1 |
| Podiatry Objective Clinical Score (POCS) | 1 |
| Pool Activity Level Checklist (PAL) | 1 |
| Power platform of Nintendo Wii fit software | 1 |
| Preparation for Decision-Making scale | 1 |
| Presence of pain scale and pain medication if resident reported pain | 1 |
| Presenting Distance Visual Acuity (PDVA) | 1 |
| Presenting Near Visual Acuity (PNVA) | 1 |
| Prospective-Retrospective Memory Questionnaire (PRMQ) | 1 |
| Psychogeriatric Assessment Cognitive Impairment Scale (PAS-CIS) | 1 |
| Psychological Wellbeing Scale (PWB) | 1 |
| Purdue Pegboard Test | 1 |
| QUALEFFO-41 questionnaire | 1 |
| Quality of Death and Dying Inventory short form (QODD) | 1 |
| Quality of vision bother domain | 1 |
| Quality of vision frequency domain | 1 |
| Quality of vision severity domain | 1 |
| Questions from validated instruments: IQCODE-N; NHG guidelines dementia | 1 |
| Quigley-Hein index (QHI) | 1 |
| Rathus Assertiveness Questionnaire | 1 |
| Rating of enjoyment on 7-pt scale | 1 |
| Rating of feeling of hunger on 7-pt scale | 1 |
| Reciprocal Reaction Time (RRT) | 1 |
| Revised UCLA Loneliness Scale (UCLA-R) | 1 |
| Risk for Healthcare-Associated Infections | 1 |
| Rivermead mobility index | 1 |
| Rosenberg self-esteem scale (RSES) | 1 |
| Route-Learning Test (RLT) | 1 |
| Rowland Universal Dementia Assessment Scale (RUDAS) | 1 |
| SARC-F scale (Strength, Ambulation, Rising from a chair, Stair climbing and history of Falling) | 1 |
| Satisfaction with food-related life (SWFL) | 1 |
| Satisfaction With Life Scale (SWLS) | 1 |
| Scale of Positive and Negative Experience (SPANE) | 1 |
| Sedentary Behavior Questionnaire | 1 |
| Self-Efficacy for Exercise Scale | 1 |
| Sense of Direction and Spatial Representation Questionnaire (SDSRQ) | 1 |
| Serial subtraction test (SST) | 1 |
| Short Assessment of Patient Satisfaction (SAPS) scale | 1 |
| Short Falls Efficacy Scale | 1 |
| Short Form Yesavage | 1 |
| Simple reaction time (SRT) | 1 |
| Simplified Oral Hygiene Index (OHI-S) | 1 |
| Situational Anxiety Inventory (STAI) | 1 |
| Slater-Usoh-Steed Presence Questionnaire | 1 |
| Smileometer (visual 5 point scale) | 1 |
| Spatial Anxiety Scale | 1 |
| Spatial Self-Efficacy Questionnaire (SSEQ) | 1 |
| Staff Nursing Home Hearing Handicap Index (NHHI) | 1 |
| Standing balance rating scale | 1 |
| State-Trait Anxiety Inventory (STAI) | 1 |
| STOPP/START criteria | 1 |
| Story retelling task | 1 |
| Strange Stories task (SST) | 1 |
| Study of Osteopathic Fractures Frailty Index (SOF) | 1 |
| Tandem stance (TS) | 1 |
| Text learning | 1 |
| Three-Item Loneliness Scale | 1 |
| Thriving of Older people Assessment Scale (TOPAS) | 1 |
| Tilburg Frailty Indicator (TFI) | 1 |
| Timed chair test | 1 |
| Timed static pedalling (TSP) | 1 |
| Timed word listing task | 1 |
| Turkish Death Anxiety Scale (TDAS) | 1 |
| UCLA Loneliness Scale | 1 |
| UKU (UKU-SERS)-Side Effects Rating Scale | 1 |
| ULS-8 Loneliness Scale | 1 |
| University of Alabama Birmingham- Life Space Assessment (UAB-LAS) | 1 |
| UpToDate - severe drug-drug-interactions categorisation | 1 |
| Urinary Distress Inventory-6 | 1 |
| Verran and Snyder-Halpern Sleep Scale (VSH) | 1 |
| Views of Informal Carers – Evaluation of Services (VOICES) | 1 |
| Visual Analogue Scale (VAS) for appetite | 1 |
| Visual Analogue Scale (VAS) for appetite & dietary satisfaction | 1 |
| Vitality index | 1 |
| Volpe-Manhold Index (VMI) | 1 |
| Waterlow Score | 1 |
| Word list learning | 1 |
| Word recognition testing | 1 |
| World Health Organization Attitudes to Aging (WHO-AAQ) | 1 |
| World Health Organization Quality of Life Scale (WHOQOL‐BREF) | 1 |
| Zung Self-Rating Depression Scale | 1 |
